# MTN-001: Randomized Pharmacokinetic Cross-Over Study Comparing Tenofovir Vaginal Gel and Oral Tablets in Vaginal Tissue and Other Compartments

**DOI:** 10.1371/journal.pone.0055013

**Published:** 2013-01-30

**Authors:** Craig W. Hendrix, Beatrice A. Chen, Vijayanand Guddera, Craig Hoesley, Jessica Justman, Clemensia Nakabiito, Robert Salata, Lydia Soto-Torres, Karen Patterson, Alexandra M. Minnis, Sharavi Gandham, Kailazarid Gomez, Barbra A. Richardson, Namandje N. Bumpus

**Affiliations:** 1 Department of Medicine, Johns Hopkins University, Baltimore, Maryland, United States of America; 2 Center for Family Planning Research and Magee-Womens Research Institute, University of Pittsburgh, Pittsburgh, Pennsylvania, United States of America; 3 South African Medical Research Council, Durban, South Africa; 4 Department of Medicine, University of Alabama at Birmingham, Birmingham, Alabama, United States of America; 5 ICAP, Mailman School of Public Health, Columbia University, New York, New York, United States of America; 6 MU-JHU Research Collaboration, Kampala, Uganda; 7 Department of Medicine, Case Western Reserve University, Cleveland, Ohio, United States of America; 8 Division of AIDS, National Institute of Allergy and Infectious Diseases, National Institutes of Health, Bethesda, Maryland, United States of America; 9 Department of Biostatistics, University of Washington, Seattle, Washington, United States of America; 10 Women’s Global Health Imperative, RTI International, San Francisco, California, United States of America; 11 School of Public Health, University of California, Berkeley, California, United States of America; 12 SCHARP, Seattle, Washington, United States of America; 13 FHI 360, Research Triangle Park, North Carolina, United States of America; Burnet Institute, Australia

## Abstract

**Background:**

Oral and vaginal preparations of tenofovir as pre-exposure prophylaxis (PrEP) for human immunodeficiency virus (HIV) infection have demonstrated variable efficacy in men and women prompting assessment of variation in drug concentration as an explanation. Knowledge of tenofovir concentration and its active form, tenofovir diphosphate, at the putative vaginal and rectal site of action and its relationship to concentrations at multiple other anatomic locations may provide key information for both interpreting PrEP study outcomes and planning future PrEP drug development.

**Objective:**

MTN-001 was designed to directly compare oral to vaginal steady-state tenofovir pharmacokinetics in blood, vaginal tissue, and vaginal and rectal fluid in a paired cross-over design.

**Methods and Findings:**

We enrolled 144 HIV-uninfected women at 4 US and 3 African clinical research sites in an open label, 3-period crossover study of three different daily tenofovir regimens, each for 6 weeks (oral 300 mg tenofovir disoproxil fumarate, vaginal 1% tenofovir gel [40 mg], or both). Serum concentrations after vaginal dosing were 56-fold lower than after oral dosing (p<0.001). Vaginal tissue tenofovir diphosphate was quantifiable in ≥90% of women with vaginal dosing and only 19% of women with oral dosing. Vaginal tissue tenofovir diphosphate was ≥130-fold higher with vaginal compared to oral dosing (p<0.001). Rectal fluid tenofovir concentrations in vaginal dosing periods were higher than concentrations measured in the oral only dosing period (p<0.03).

**Conclusions:**

Compared to oral dosing, vaginal dosing achieved much lower serum concentrations and much higher vaginal tissue concentrations. Even allowing for 100-fold concentration differences due to poor adherence or less frequent prescribed dosing, vaginal dosing of tenofovir should provide higher active site concentrations and theoretically greater PrEP efficacy than oral dosing; randomized topical dosing PrEP trials to the contrary indicates that factors beyond tenofovir’s antiviral effect substantially influence PrEP efficacy.

**Trial Registration:**

ClinicalTrials.gov NCT00592124

## Introduction

Four recently completed clinical trials demonstrated the effectiveness of both vaginal and oral tenofovir (TFV)-containing regimens as pre-exposure prophylaxis (PrEP) to prevent HIV infection in susceptible men, women, and partners of HIV-infected individuals [1], [2], [3], [4]. Relative risk reduction varied widely: 39% in women using vaginal TFV gel before and after sex (CAPRISA 004) [1], 44% in men who have sex with men (MSM) using daily oral TFV disoproxil fumarate (TDF)/emtricitabine (FTC) (iPrEX) [2], 62% or 75% with daily use of TDF/FTC in men and women whose heterosexual partner(s)’ HIV-1 serostatus was either unknown (CDC TDF2) [4] or known positive (Partner’s PrEP) [3], respectively. In contrast, all or part of two other studies of women using daily TDF/FTC tablets (FEM-PrEP) [5], daily TDF tablets (VOICE) [6], or daily TFV vaginal gel (VOICE) [7] were stopped early for futility.

Knowledge of active drug at the site of action, arguably the vaginal tissue in women, linked with seroconversion events in these clinical trials could provide critical information for interpreting outcomes and guiding dose and frequency decisions for future clinical trials by indicating the critical concentration needed to prevent infection. To date, none of these clinical trials have reported the concentration of active drug in the site of action due largely to logistical constraints in these large clinical studies.

Drug concentration at anatomic sites more distant from the rectal or vaginal mucosal tissue, however, was associated with HIV seroconversion events in all trials where pharmacokinetic (PK) results have been reported. Detecting drug in plasma or peripheral blood mononuclear cells (PBMCs) was associated with significantly higher relative risk reduction when compared to the primary modified intent-to-treat analysis in iPrEX (92% vs. 44%) and Partner’s PrEP (86% vs. 67% for TDF, 90% vs. 75% for TDF/FTC)(all p values <0.05) [2], [8]. In CAPRISA 004, tenofovir concentration in cervicovaginal fluid greater than 1,000 ng/mL was associated with increased efficacy [9]. Bridging data that connects drug concentrations in these more distant sites – blood and cervicovaginal fluid - to vaginal and rectal tissue in association with seroconversion events is needed to identify target drug concentrations in the mucosal tissue sites associated with protection of HIV infection. This knowledge would be of substantial benefit to future PrEP drug development.

We report the results of such a PK bridging study, MTN-001, which directly compares vaginal TFV gel and oral tenofovir disoproxil fumarate (TDF) tablets in a cross-over design thus removing inter-individual variation to provide more precise paired comparisons. We measured steady-state pharmacokinetics of TFV and its active form, TFV diphosphate (TFV-DP), in blood, vaginal tissue, vaginal lumen, and rectal lumen to better understand concentrations in a wide range of anatomic locations after different routes of dosing. The substantially different TFV concentrations in blood and tissue after oral compared to vaginal dosing was successfully described.

## Materials and Methods

The protocol for this trial and supporting CONSORT checklist are available as supporting information; see Checklist S1 and Protocol S1.

### Ethics Statement

As this research involved human subjects, written informed consent was obtained from all research participants and the clinical investigation was conducted according to the principles expressed in the Declaration of Helsinki. The study was conducted following US National Institutes of Health and local IRB approval at seven clinical sites: Umkomaas and Botha’s Hill, Durban, South Africa; Makerere University-Johns Hopkins University Research Collaboration, Kampala, Uganda; Case Western Reserve University in Cleveland, OH, USA; University of Pittsburgh, Pittsburgh, PA, USA; University of Alabama at Birmingham, Birmingham, AL, USA; Bronx-Lebanon Hospital Center, New York City, NY, USA. The study is registered at ClinicalTrials.gov (Identifier NCT00592124). The full protocol is available online: http://www.mtnstopshiv.org.

### Study Schema

MTN-001 was a 21-week, phase 2, open-label, crossover study in which all participants were assigned to a randomized sequence of daily tenofovir orally, vaginally, or both orally and vaginally in three 6-week study periods, separated by one-week washouts between study periods (Table 1). Adherence to and acceptability of various routes of TFV administration was also assessed, but is reported in detail elsewhere along with supplemental PK data used for adherence assessment [10]. Microbicide Trial Network (MTN) laboratories at The Johns Hopkins University and the University of Pittsburgh performed drug assays and flow cytometric analyses, respectively. The clinical phase of the study began in June 2008 and finished in July 2010 when the final enrolled participant completed all study visits.

**Table 1:** MTN-001 Study Schema.

	N	Period 1	Washout	Period 2	Washout	Period 3
		6 weeks	1 week	6 weeks	1 week	6 week
Sequence A	24	Oral	–	Vaginal	–	Oral+Vaginal
Sequence B	24	Vaginal	–	Oral	–	Oral+Vaginal
Sequence C	24	Oral+Vaginal	–	Oral	–	Vaginal
Sequence D	24	Oral+Vaginal	–	Vaginal	–	Oral
Sequence E	24	Oral	–	Oral+Vaginal	–	Vaginal
Sequence F	24	Vaginal	–	Oral+Vaginal	–	Oral

Formulations: Oral, 300 mg tenofovir disoproxil fumarate; Vaginal, 1% tenofovir gel.

Sampling occurs at the 3-week mid-point (blood only) and 6-week end of period visit (blood, PBMC, vaginal biopsy [intensive sites], vaginal fluid, and rectal fluid [Bronx-Lebanon site]).

Number of samples at each visit varied between intensive (US) and non-intensive (African) clinical sites.

### Study Participants

Women were recruited through clinical research sites and community outreach. After the nature and possible consequences of the study was explained, all participants provided written informed consent prior to screening and study enrollment. Eligible participants were women aged 18–45 years, HIV-uninfected, sexually active, non-pregnant, and using an effective method of contraception. Participant self-identified race/ethnicity was recorded. Major exclusion criteria included: pregnancy, significant blood chemistry or hematology laboratory abnormalities, hepatitis B surface antigen positivity, clinically apparent gynecological abnormality, sexually transmitted or urinary tract infections requiring treatment, recent use or intended use of specific forms of contraception (diaphragm, vaginal ring, spermicide) or use of non-study vaginal products.

Participants evaluable for the final analysis included women who were dispensed study product and returned to report on product use at least once in each of the three study periods.

### Study Procedures

Eligible research participants were randomized equally to one of 6 sequences of oral, vaginal, and combination dosing of study drug for each of the three 6-week periods. Randomization, conducted by the data coordinating center, was stratified by site. Blocks of size 6 and 12 were chosen randomly to distribute the six treatment sequences. The uniform distribution was used to generate the random assignments. Sites received sealed, numbered randomization envelopes that were assigned in sequential order to each participant at the time of enrollment. The randomized dosing sequence was revealed to the study team and research participant after receipt of randomization envelope. No drug was taken during a one week washout between study periods (Table 1). Tenofovir disoproxil fumarate tablets (VireadTM), 300 mg, were provided by Gilead Sciences (Foster City, CA). Tenofovir gel was provided by CONRAD (Arlington, VA). Participants were instructed to take study product(s) once daily before bedtime or the longest period of rest.

Study visits took place at enrollment, at the 3-week midpoint, and again at the end of each six-week study period, and after the final one-week washout period for a total duration of 21 weeks. The PK sampling took place at the 3-week mid-period and 6-week end-of-period visits. Blood for serum and PBMCs was collected at the mid-period visits. More intensive end-of-period sampling differed by clinical research site. At the African sites, blood (serum, PBMCs) was collected prior to a dose in clinic and during one specified randomized time interval (1–3, 3–5, or 5–7 hours post dose) and repeated at the same time in each study period. Cervicovaginal lavage (CVL) fluid was collected in the same specified time interval as the blood collection. At the US sites, more intensive sampling was performed, with blood (serum, PBMCs) collected pre-dose and 1, 2, 4, 6, and 8 hours after dosing in clinic for each study period. In addition, CVL, endocervical cytobrush sampling (ECC), and 2 vaginal tissue biopsies were collected from each participant in each study period at one randomly assigned time (pre-dose, 2, 4, and 6 hours after dosing). US sites collected PBMCs for flow cytometry assessment at end-of-period visits. At the Bronx-Lebanon site, rectal sponges were collected to coincide with scheduled vaginal sampling.

#### Blood collection

Blood was collected in clot tubes and PBMCs were isolated from whole blood using cell preparation tubes (CPTs). Serum was processed by centrifugation and aliquoting into cryovials. Cells were isolated from the buffy coat after centrifugation of the CPTs, washed twice in phosphate buffered saline (PBS), counted, and then lysed in 70% methanol before storage in cryovials. All samples were frozen at −80°C until analysis.

#### Vaginal sample collection

Lavage fluid from a 10 mL saline CVL was collected into a syringe, transferred into a 15 mL conical tube, centrifuged, and the supernatant was aliquoted into cryovials. For the ECC sample, a cervical cytobrush was inserted into the cervical os, rotated twice through 360 degrees and removed. The brushes were immersed in PBS with gentle agitation to remove cells. Cells were washed twice in PBS, counted, lysed using 70% cold methanol and aliquoted into cryovials. Two vaginal wall biopsies were taken using 3×5 mm vaginal biopsy forceps and placed in a cryovial. All vaginal samples were flash frozen and stored in a −80°C freezer. At the time of sample analysis, the biopsies were weighed and homogenized with an electric mortar and pestle in a 1.5 mL cryovial with 500 µL ice-cold 70% methanol. Cervicovaginal lavage results were corrected for estimated average 20× dilution of 0.5 mL cervicovaginal fluid in 10 mL lavage fluid [11].

#### Rectal fluid collection

Sponges (Ultracell® Medical Technologies, North Stonington, CT) pre-wetted with PBS were applied to the rectal mucosa through an anoscope for 5 minutes to adsorb rectal fluid. Sponges were centrifuged and the rectal fluid removed was stored at −80°C until analysis. TFV concentrations were not corrected for adsorbed volume, due to variable estimated sample weights due to evaporative differences across samples.

### Drug Analysis

TFV and TFV diphosphate (TFV-DP) concentrations were determined by previously described LC-MS/MS methods [12], [13] validated for all matrices by the Johns Hopkins Clinical Pharmacology Analytical Laboratory. TFV and TFV-DP assays meet the FDA bioanalysis guidance values of ≤ ±15% for precision and accuracy. (See Table S1 for assay performance.).

#### Tenofovir

Thawed aliquots of serum, and tissue homogenate, with ^13^C_5_-TFV internal standard, were protein precipitated with methanol. CVL and rectal fluid aliquots with^13^C_5_-TFV underwent solid phase extraction using HLB oasis cartridges. The supernatants and eluants were collected and dried and reconstituted in 0.5% acetic acid for analysis. Samples underwent chromatographic separation using gradient elution with a Zorbax Eclipse XDB-C18 column, with positive electrospray ionization (ESI), and detection via multiple reaction monitoring using an LC-MS/MS system (Waters Acquity UPLC and Agilent 1100 HPLC Applied Biosystem API4000 mass spectrometer). Calibration standards for assay ranged from 0.31–1280 ng/ml. (0.25–50 ng/sample for tissue).

#### Tenofovir diphosphate (TFV-DP)

Cell lysates and tissue homogenates were analyzed using an indirect assay, which measures TFV in the sample after isolation of TFV-DP and enzymatic conversion to TFV essentially. TFV-DP was isolated from cell lysates on a Waters QMA cartridge (Waters Corporation, Milford MA) over a salt (KCl) gradient. TFV and TFV monophosphate (TFV-MP) were eluted from the cartridge under lower salt concentrations followed by elution of TFV-DP with application of 1 M KCl to the cartridge. Isolated TFV-DP was then enzymatically dephosphorylated to TFV via phosphatase digestion with sweet potato phosphatase with ^13^C_5_-TFV internal standard. Samples were desalted using trifluoroacetic acid and eluted with 1 mL methanol. The effluent was dried and reconstituted in 0.5% acetic acid for analysis. Processed samples were then chromatographically separated using a reverse phase Waters BEH C18 column and TFV and IS were ionized using negative ion mode in electrospray ionization and detected via multiple reaction monitoring (Waters Acquity UPLC, Applied Biosystem API5000 mass spectrometer) The assay is linear over the range of 50.0–1,500 fmol TFV-DP/sample.

Conversions of concentrations to common molar units assume equivalent density for one gram tissue and one milliliter fluid with the following cell volume estimates: PBMC, 0.28 pL per cell, ECC cells, 2.8 pL, which assumed an arbitrary 50∶50 mix of stratified squamous, 4.8 pL, and columnar, 0.8 pL, epithelial cells [14], [15], [16], [17].

### Flow Cytometry

Flow cytometry was performed at the US sites using either FACSCalibur or Canto flow cytometers (Becton-Dickinson, Franklin Lakes, NJ) and the following reagents (Becton-Dickinson): CD38 PE (347687), HLA-DR FITC (347363 [L243]), CD3 PerCP (347334 [SK7]), CD4 APC (340443), IgG2a FITC (349051), and IgG1 PE (349043). Results were expressed as percent (using blood total lymphocyte count) and absolute counts for CD3, CD4 (CD3/CD4), CD38 (CD3/CD4/CD38), HLA-DR (CD3/CD4/HLA-DR), and CD38/HLA-DR. Mean fluorescence intensity (MFI) was assessed for CD38 and HLA-DR.

### Data Analysis

Primary TFV PK outcome measures were descriptive concentration-time curves for analytes, peak concentration (C_max_), time to peak concentration (T_max_), pre-dose concentration (C_pre-dose_) based on individual data for intensive sites and composite concentration data from research participants at more sparsely sampled non-intensive sites. Sample size was based on adherence and acceptability outcomes, not PK outcomes, and is described elsewhere [18]. For PK outcomes, 72 intensive sampling participants provide 90% power with 5%, 2-sided alpha error to detect a 0.38 standard deviation unit difference between regimens assuming intra-individual correlation. This represents a 14% difference in C_max_ and AUC based on variation in prior reports. The statistical power of paired comparisons using all 144 participants would be greater. Serum and PBMC concentrations from US sites were used to estimate Cmax and Tmax using WinNonlin software (Version 5.0, Pharsight, Inc., Cary, North Carolina). Concentration prior to an observed clinic dose, C_pre-dose_, was not termed C_tau_ because participants were instructed to take their doses before sleep and these samples were collected during the day at the research sites, prior to the end of the prescribed dosing interval. Nearly all drug concentrations in all matrices were skewed upward, thus, log_10_ transformations of drug concentrations were used in all models comparing across treatment regimens. To provide the relative magnitude of paired differences between routes of dosing, we estimated concentration ratios. In some cases, these ratios used values between the limit of detection (LOD, 5 times background) and the LOQ, but only if the higher of the concentration pair was above the LOQ. We chose this approach, cognizant of some loss of precision, based on sensitivity analyses in which exclusion of LOQ values (due to incalculable ratios with imputed “0” denominator values) or imputation of an arbitrary non-zero value (LOQ, LOQ/2) for values below the LOQ resulted in more extreme values or significantly increased variability. In nearly all cases, using the actual values between the LOD and LOQ provided ratios between the values obtained using value imputation.

In order to provide a rough estimate of very recent adherence, we estimated the time elapsed between the dose prior to the research clinic visit and the TFV serum sample collected during that upcoming visit. We used the observed peak serum TFV concentration as a crude estimate of the peak TFV concentration associated with the prior dose and estimated the time it would take for this peak concentration to fall to the observed pre-dose concentration in the research clinic. With only 8 hours of post-dose concentration data in our design, we were not confident of our half-life estimates using traditional PK analyses; so, we used a range of population-based half-life estimates, 12–17 hours [19], [20]. While this approach assumes steady-state conditions, there were only minor differences in estimated time – less than 10% if we inflated the peak concentrations to simulate steady-state peak concentration with 100% daily adherence (slightly longer elapsed time) and no difference if we assumed no dose accumulation at the time of the prior dose similar which is comparable to the single dose peak estimates which can be reasonably estimated by peak-trough differences with TFV’s rapid absorption. Sparse sampling PK models are in development to provide more precise model based estimates, but these estimates bracket timing of recent doses using population-based data.

Adverse events were compared across treatment regimens using conditional logistic regression controlling for study period and randomization sequence. Drug concentrations and T_max_ were compared across drug regimens using linear mixed effects models controlling for study period and sequence of randomization. Treatment regimen by period interactions were assessed and were not statistically significant for any of the outcomes. In addition, there were no statistically significant period or sequence effects for any of the outcomes. Correlations between different measures were assessed using Spearman rank correlation coefficient. Friedman’s test was used to test for differences in drug concentrations over time. All statistical tests were performed using IBM SPSS Statistics (v. 19, IBM Corporation, Somers, NY) with p value ≤0.05 indicating statistical significance.

## Results

### Study Design and Research Participants

**Figure 1:**
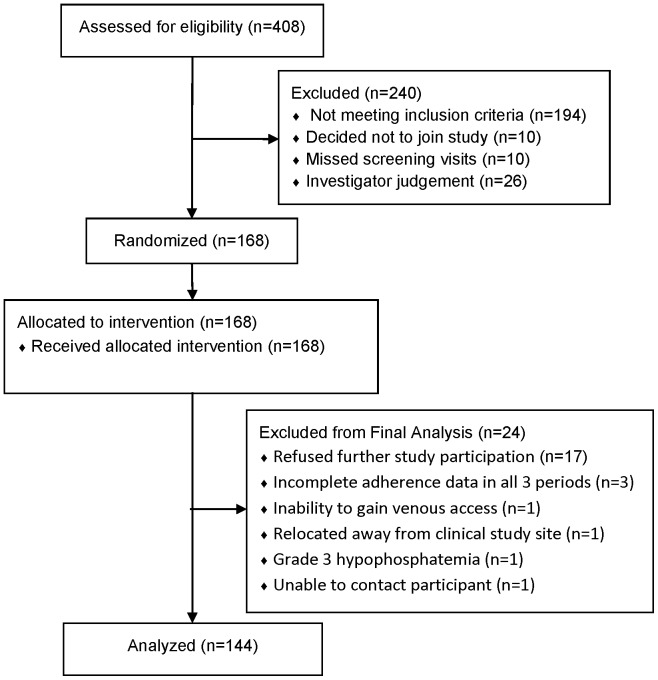
Accounting of research participants from screening to data analysis.

In MTN-001, each research participant was prescribed open label daily TFV in a series of 3 different regimens, each for a 6-week period followed by a one-week washout between periods (Table 1). A different TFV formulation or combination of formulations was prescribed for each of these periods: oral 300 mg TFV disoproxil fumarate (Viread™, TDF), vaginal 1% TFV gel, and a combination of both oral and vaginal formulations. The sequence in which the women were prescribed each regimen, before crossing over to the next of 3 regimens, was determined by randomization**.** At the end of each 6 week period, blood, peripheral blood mononuclear cells (PBMC), vaginal tissue, vaginal fluid, and rectal fluid were collected with sample type and number of samples depending on site capacity. Across the 7 participating clinical sites (3 in Africa and 4 in the US), 408 women were screened, 168 were enrolled and randomized to study drug, 24 of whom did not complete enough of the study to be evaluable (adherence data and drug dispensing in all 3 periods) and were replaced to yield the planned total participation of 144 women (Figure 1). Of these 24 participants who were replaced, 17 refused further participation, 3 completed the study with incomplete adherence data or drug dispensing in all 3 study periods, 1 was relocated from study site, 1 was lost to follow-up, 1 had venous access problems, 1 stopped for grade 3 hypophosphatemia. There were no socio-demographic differences between the 144 evaluable participants and the 24 who were replaced. Half of research participants (N = 72) were enrolled in US sites and half at the African sites. Research participant characteristics are summarized in Table 2. Each scheduled study visit following enrollment was attended by 97% to 100% of the participants. Overall, specimens for PK analysis were collected at 99% of planned study visits. Study product was put on hold by the study team for protocol directed reasons (adverse event or treatment of sexually transmitted infection) for 11 (8%) evaluable research participants for a median (IQR) duration of 7 (3, 8) days.

**Table 2:** Subject Characteristics.

Characteristic	median (IQR)	# (%)
Age (years)	29 (25–37)	
Weight (kg)	73 (65–88)	
CrCl (mL/min)	122 (105–146)	
Self-identified Race/Ethnicity		
Black		97 (67)
White		32 (22)
Asian		8 (6)
Multi-racial		6 (4)
Hispanic		1 (1)
Contraceptive method		
Injectable		62 (43)
Oral		42 (29)
Surgical sterilization		22 (15)
IUD		14 (10)
Male partner sterilization		4 (3)

### Adverse Events

All 3 regimens were well tolerated with mild, transient symptoms with minimal differences among regimens. The adverse event profile among the 24 who did not complete the study did not differ from the study population as a whole. Transient and mild nausea was more frequent in the oral (15%) and combination periods (14%) when compared to the vaginal period (3%) (p<0.001). Headache was also more frequent during the combination dosing period (8%) compared to vaginal dosing periods (2%) (p<0.01) with intermediate frequency with oral only dosing (5%). Hypophosphatemia was the most commonly reported adverse event, but did not differ in frequency among regimens: 11% vaginal, 15% oral, 15% combination (p>0.05). Between screening and enrollment, prior to TFV dosing, phosphate was variable and differed as much as 2 mg/dL.

### Drug Time Course

**Figure 2:**
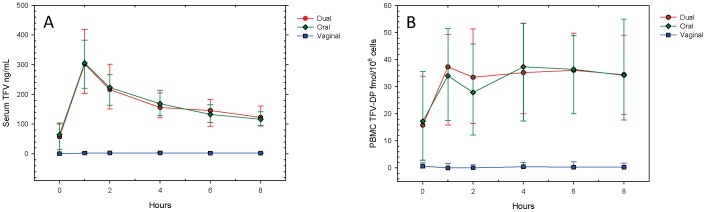
Serum TFV and TFV-DP concentration versus time. Serum TFV (panel A) and PBMC TFV-DP (panel B) concentration versus time plots are shown for the observed 8 hour interval following a dose in clinic according to dosing regimen. Median with asymmetric upper and lower quartiles is shown. Values are only for the 70 US participants where all 6 PK samples were collected.

Serum TFV concentrations following observed dosing in clinic were quantifiable in all participants in all study periods (Table 3). Pre-dose concentrations, however, were not quantifiable in 20–38% of participants depending on dosing regimen and were statistically different when comparing vaginal to either oral or combination dosing regimens ((p<0.001). Intensive pharmacokinetic sampling (vaginal biopsies and more frequent blood collection) was performed only at the US sites. At intensively sampled sites during oral and combination dosing study periods, median (IQR) serum TFV concentrations peaked at 1.0 hour (1.0–1.2) and 1.0 hour (1.0–1.4), respectively, followed by a log-linear decline over the next 8 hours (Figure 2A). In the vaginal only study period, the serum concentration peaked later, at 2.1 hours (1.9–4.6), followed by a slight decline at the last 8 hour sampling time.

**Table 3:** Summary of TFV and TFV-DP concentrations at all sampled anatomic sites.

		Oral		Combination		Vaginal	
Matrix/Moiety/Parameter	Units	median (IQR)	%>LLOQ	median (IQR)	%>LLOQ	median (IQR)	%>LLOQ
Serum TFV C_max_	ng/mL	332 (257–406)	100	337 (257–447)	100	3.9 (2.2–7.9)	100
Serum TFV C_pre-dose_	ng/mL	65 (14–103)	80	57 (2–101)	77	0.67 (<0.3–2.09)	62
Serum TFV T_max_	hours	1.0 (1.0–1.2)	–	1.0 (1.0–1.4)	–	2.1 (1.9–4.6)	–
PBMC TFV-DP C_max_	fmol/10^6^ cells	51 (28–74)	99	49 (31–70)	100	<6 (<6–<6)	17
PBMC TFV-DP C_pre-dose_	fmol/10^6^ cells	17 (3–36)	66	16 (3–34)	63	<6 (<6–<6)	7
PBMC TFV-DP T_max_	hours	4.0 (2.0–6.1)	–	4.0 (1.0–6.0)	–	3.9 (1.1–6.1)	–
Tissue TFV	ng/mg	0.15 (<0.15–0.27)	50	104 (40–268)	96	113 (27–265)	94
Tissue TFV-DP	fmol/mg	<25 (<25–<25)	19	2,464 (917–6,112)	96	1,807 (591–5,860)	90
Cervicovaginal lavage	ng/mL	5,380 (<6–201,560)	59	1.6×10^6^ (0.5×10^–^6.5×10^6^)	98	3.1×10^6^ (0.6×10^6^–8.1×10^6^)	97
Endocervical Cytobrush	fmol/10^6^ cells	<130 (<130–<130)	18	903 (159–4,283)	62	1,181 (147–5,418)	68
Rectal Sponge	ng/sponge	20 (7–404)	83	576 (140–2887)	100	119 (53–2150)	100

Data from end-of-period visit showing median (interquartile range) by dosing regimen in common concentration units. Serum TFV C_pre-dose_, PBMC TFV-DP C_pre-dose_, and cervicovaginal lavage include African clinical sites; other parameters are calculable only for US clinical sites. Rectal sponges are only from one US site. For values below the LLOQ,<[median LLOQ] is shown. Cervicovaginal lavage results were corrected for estimated average 20× dilution of 0.5 mL cervicovaginal fluid in 10 mL lavage fluid [11].

In contrast, the median intracellular PBMC TFV-DP concentrations changed only between the pre-dose and 1-hour sample without further change through 8 hours (Figure 2B). TFV-DP concentrations were quantifiable in all participants during oral and combination dosing study periods, but pre-dose TFV-DP concentrations were not quantifiable in 34% and 37% of participants in oral and combination dosing study periods, respectively. The median concentration-time profile of PBMC TFV-DP in the vaginal period could not be described since it was quantifiable in only 7% of participants pre-dose and 17% of participants following dosing.

For drug concentration in vaginal tissue homogenate, endocervical cytobrush cell lysate, and cervicovaginal lavage fluid, median concentration at each time was not different among the times from pre-dose to 8 hours post-dose (p>0.05). By contrast with blood, oral dosing achieved the lowest drug concentrations in vaginal tissue, endocervical cytobrush cells, and cervicovaginal lavage samples where the lower quartile was not quantifiable in any of these matrices. With vaginal or combination dosing, however, TFV and TFV-DP was quantifiable in 90 to 95% of tissue homogenates, 97 to 98% of CVL samples, and 62 to 68% of endocervical cytobrush cell samples.

**Figure 3:**
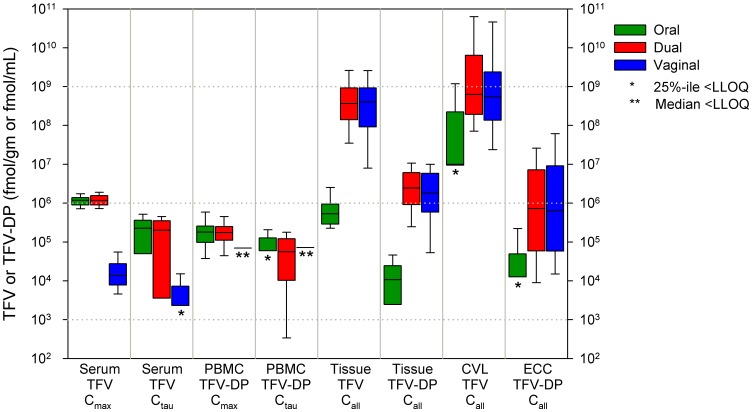
Boxplots of TFV and TFV-DP concentrations by anatomic site. Side-by-side boxplots of end-of-period visit data for all participants by anatomic site and dosing regimen are shown. Each box indicates the interquartile range with center bar as median and whiskers 1.5 times the quartile. *Lower quartile (LQ) is below the limit of quantitation (LOQ), only median and above are shown. **Median is below LOQ, so the median of values above the LOQ are shown as a single bar. X-axis key: *anatomic location*, PBMC peripheral blood mononuclear cells, CVL cervicovaginal lavage, ECC endocervical cytobrush; *drug moiety*, TFV tenofovir, TFV-DP tenofovir diphosphate; *sample timing*, C_max_ peak concentration following dose at clinic visit, C_pre-dose_ concentration prior to dose at clinic visit, C_all_ pools values from all participants regardless of scheduled time relative to dose.

Because there were no temporal trends in locations other than blood and each subject provided only one sample from these locations at each end-of-period visit, results from tissue and cervicovaginal lavage fluid are summarized as median of values from all times in Table 3. Drug concentrations for each study period are compared across all anatomic sites using common molar units to allow comparison of TFV to TFV-DP in Figure 3.

Because each daily dose was to be taken at the hour of sleep and clinic visits usually occurred in the morning, C_pre-dose_ is not a true trough (C_tau_), but is a sample between C_max_ and C_tau_. The C_pre-dose_ sample occurs 13 (10–14) hours (median [IQR]) following the prior dose according to subject self-report – an interval consistent with the protocol. Similarly, C_max_ is also potentially different than a true steady-state C_max_ since it follows a short dosing interval. Assuming excellent adherence and steady-state, both C_pre-dose_ and C_max_ will be overestimates. Poor adherence, however, would have an opposite, depressing effect on concentrations. Pre-dose concentrations are influenced both by individual pharmacokinetics and by the timing of prior doses with progressively greater influence of the more recent doses. We estimate that the median interval of time between the oral dose taken prior to the research visit blood collection was 1.2 to 1.7 days; the interval for the prior vaginal dose was between 1.3 to 1.8 days.

### Rectal Fluid

Rectal fluid was collected at only one US site. Rectal sponge TFV concentrations were quantifiable in 10 of 12 participants (83%) in the oral dosing study period and in all participants in the combination and vaginal dosing study periods. Median concentration between combination and oral dosing study periods were statistically significantly different (p = 0.008), as were mean concentrations between the vaginal and oral study periods (p≤0.03) (Table 3).

### Dosing Route Comparisons

Using linear mixed effect models controlling for study period and randomization sequence after log_10_ transformations of the end-of-period concentration data, vaginal only dosing was different than oral only dosing for all matrices tested (all p<0.001, except rectal sponge p = 0.03). Combination dosing was only different compared to vaginal only dosing for serum and PBMC’s (p<0.001) and compared to oral only dosing for tissue, CVL, endocervical cytobrush, and rectal (all p<0.001, except rectal p = 0.008). The relative magnitude of the dose regimen relationships across matrices via paired individual ratios are provided in Table 4. The paired individual oral:combination concentration ratios for serum TFV and PBMC TFV-DP were unity while the oral and combination regimens achieved 58 and 56 fold greater TFV serum concentrations, respectively, compared to vaginal dosing. The PBMC ratio for oral and combination compared to vaginal study periods was lower, 17 and 15, respectively, and far fewer pairs were available for these ratios given the low frequency of quantifiable vaginal dosing PBMC concentrations. In vaginal tissue samples, the picture was reversed with vaginal:combination dosing ratios near unity for TFV and TFV-DP. The median combination and vaginal dosing concentrations were 635–797 times greater for TFV and 130–173 times greater for TFV-DP when compared to oral-only dosing (Table 4). The vaginal:oral dosing ratios for CVL were not as large. We corrected CVL results for an estimated 20-fold dilution by the 10 mL lavage fluid added to 0.5 mL native cervicovaginal fluid [11], but this overestimates dilutional effects when there is up to 3.5 mL residual TFV gel for which a smaller dilution correction is needed. Complete pairs were available for only 58 of 144 (40%) participants to calculate vaginal:oral endocervical cytobrush cell ratios.

**Table 4:** Concentration ratios between dosing period by anatomic location.

		Vaginal:Oral		Vaginal:Combination		Combination:Oral	
PK Parameter	Maximum N	N	Median (IQR)	N	Median (IQR)	N	Median (IQR)
Serum TFV	560	450	0.017 (0.008–0.034)	449	0.018 (0.007–0.035)	462	1.0 (0.8–1.3)
PBMC TFV-DP	560	260	0.06 (0.02–0.21)	251	0.07 (0.02–0.20)	452	1.0 (0.6–1.7)
Tissue TFV	70	63	797 (162–1623)	63	1.1 (0.3–3.3)	65	635 (179–1,474)
Tissue TFV-DP	70	49	130 (19–425)	64	0.8 (0.3–2.1)	50	173 (37–424)
CVL TFV	140	52	51 (3–365)	93	1.1 (0.6–2.8)	54	79 (5–391)
CB TFV-DP	70	28	2.2 (0.8–16.6)	54	0.9 (0.4–4.2)	33	3.6 (0.4–11.8)
Rectal TFV	12	10	6.1 (0.4–15.9)	12	0.3 (0.2–4.0)	10	3.1 (1.9–52.8)

Pairs are included if at least one is above the limit of assay quantitation and the other is above the limit of assay detection. Other pairs are excluded. Serum and PBMC ratios are based on pooled values for data pairs from all times with research participants contributing more than one pair.

### Phosphorylation Ratios

The ratio of TFV to TFV-DP in blood and tissue sources were all greater than unity with the lowest ratio seen in the pre-dose serum TFV to PBMC TFV-DP ratio, with median values of 2.5 (oral only) and 2.9 (combination) (Table 5). Blood ratios were sensitive to timing given the unsynchronized concentration-time pattern of serum TFV and PBMC TFV-DP. Also, it should be noted that these PBMC assessments do not address the TFV monophosphate moiety which is present, but unmeasured here. Higher TFV:TFV-DP ratios were observed in tissue. Presumably, intracellular TFV-DP was diluted by extracellular constituents and cell volume after tissue homogenization whereas assessments in PBMC were adjusted only for lymphocyte cell volume. Because CVL and ECC concentration values were subject to differential lavage dilution based on dosing regimen and uncertain epithelial cell volume, respectively, the CVL:ECC TFV:TFV-DP ratio was not estimated.

**Table 5:** Ratio of unphosphorylated TFV to phosphorylated TFV-DP by anatomic sampling sites.

		Oral		Combination		Vaginal	
Molar Ratio	Maximum N	N	Median (IQR)	N	Median (IQR)	N	Median (IQR)
Serum TFV/PBMC TFV-DP C_max_	70	65	5.9 (3.6, 10.1)	69	6.2 (4.1, 9)	*10	NA
Serum TFV/PBMC TFV-DP C_pre-dose_	144	89	2.5 (1.6, 4.5)	104	2.9 (1.7, 5.3)	*6	NA
Serum TFV_all_/PBMC TFV-DP_all_	560	475	4.8 (2.6, 9.5)	479	4.2 (2.6, 9)	245	1.2 (0.4, 4.6)
Tissue TFV/Tissue TFV-DP	70	27	48 (22, 130)	67	170 (63, 418)	64	207 (84, 485)

Pairs are included if at least one value in the pair is above the limit of assay quantitation and the other is above the limit of assay detection. “N” is the number of pairs available meeting the inclusion criteria above. “Maximum N” is the number of possible pairs if all subjects at all visits provided a sample. C_max_ only includes US participants where multiple serum samples were available; the pair is defined by serum C_max_ matched with the corresponding PBMC TFV-DP concentration which is not necessarily peak TFV-DP after the same dose. Subscript “all” indicates that all PBMC-Serum pairs from each sample times are included, with participants contributing multiple pairs. All p<0.001 Wilcoxon signed rank test for TFV fmol/gm vs. TFV-DP fmol/gm. *Insufficient samples above the limit of assay quantitation and detection to estimate reliable medians given the inevaluable excluded pairs.

### Flow Cytometry

Initial median (IQR) values for PBMC surface markers were absolute lymphocyte count 2209 (1827, 2722), %CD3 77 (74, 81), %CD4 49 (44, 54), %CD38 66 (51, 78), %HLA-DR 5 (3, 7), %CD38/HLA-DR 3 (2, 4). There were no differences among the 3 study periods for any cell surface marker (p>0.1). When assessed within each dosing regimen, mean fluorescence intensity values for both CD38 and HLA-DR on PBMCs were negatively correlated with TFV-DP tissue concentrations (all r_s_ >0.34, all p<0.001), but these surface markers did not correlate with TFV-DP concentration in PBMC or endocervical cytobrush cells. There was no correlation of percent or absolute number of either CD4 or CD8 with TFV-DP in any anatomic site by any route of dosing.

## Discussion

Our cross-over design allows for the direct paired comparisons of TFV PK within individuals in multiple anatomic sites, avoiding inter-individual variability. The most significant finding is the greater than 100-fold higher concentration of the active TFV moiety, TFV-DP, in vaginal tissue homogenates with vaginal dosing when compared to an oral regimen alone. Conversely, systemic exposure (serum TFV) after vaginal dosing was at least 56-fold lower than after oral dosing regimens. This difference in systemic exposure based on route of dosing may have been the cause of the greater frequency of nausea, diarrhea, and headache with oral regimens, though the gastrointestinal symptoms may have been due to local gastrointestinal effects in oral dosing regimens. The large systemic exposure differences, yet with similar rates of hypophosphatemia with both oral and vaginal regimens, argues against a significant dose-dependent relationship between TFV and hypophosphatemia.

The temporal patterns of drug concentration we observed are consistent with expectations based on the relatively shorter 12–17 hour serum half-life of TFV [19], [20], resulting in large concentration undulations over 8 hours, and a far longer half-life of several days for TFV-DP [21], [22], resulting in essentially flat concentrations over an 8 hour interval. One methodological concern in topical dosing studies is contamination of tissue biopsies by luminal drug retained on the biopsy despite washing. Our results suggest that this effect is minor, at best, given the relatively high TFV-DP concentration (which requires cellular uptake for phosphorylation) in vaginal biopsies.

The amount of TFV extracted from rectal sponges was 3 times higher with combination dosing when compared to oral dosing, though vaginal dosing was not statistically different from oral or combination dosing. This may result from an additive effect of oral and vaginal dosing not seen in any other anatomic site, but may also have resulted from assay variability due to the collection method and should be cautiously interpreted. Use of an internal standard in the rectal sponge would greatly improve the accuracy of this sampling method. Without this, heterogeneity of both fluid volume adsorbed onto the sponge and subsequent evaporative losses prior to sample freezing may introduce significant and unmeasured variation which prevents accurate estimates of concentration. Regardless, the finding of greater or similar rectal fluid concentrations after vaginal dosing alone compared to oral dosing suggests that vaginal dosing may also provide some level of protection from receptive anal intercourse. A similar vaginal to rectal drug migration has been reported by Nuttall, *et al*., in macaques where rectal fluid TFV concentrations were 1 to 2 log_10_ lower than vaginal fluid TFV concentrations following vaginal dosing [23].

The most notable result of the study is the 100-fold greater TFV-DP concentrations in vaginal tissue associated with vaginal dosing. This more precise estimate from our cross-over design confirms what can be inferred from combining results of separate oral dosing studies [24] with vaginal dosing studies [25]. Related to this, combination dosing conferred no concentration benefits in vaginal tissues. Considered in isolation, this finding of higher vaginal tissue concentrations with vaginal dosing anticipates both an increased level of protection from acquisition of HIV infection and a regimen more tolerant of missed doses or planned intermittent dosing with vaginal dosing compared to oral dosing. This assumes: (1) drug concentration in vaginal tissue is relevant to HIV protection; (2) tissue drug concentrations with oral dosing are not 100 times greater than needed for HIV protection (beyond the plateau on the concentration-response curve); and (3) no negative effect on HIV transmission at higher drug concentrations or associated with the vaginal gel vehicle.

The modest (CAPRISA 004) or absent (VOICE) efficacy seen in the TFV gel studies, despite vaginal tissue levels expected (based on MTN-001) to be far higher than in the very successful Partner’s PrEP and TDF2 studies, suggests that efficacy is more complex than would be predicted by vaginal tissue concentration alone [1], [3], [4], [7]. The disparate oral v. topical trial results, despite vaginal tissue concentration expectations, may be evidence that vaginal tissue concentrations are not, in fact, the critical site of ARV action in PrEP. Systemic PBMC or draining cervicovaginal lymph nodes, each of which would have higher TFV-DP concentrations with oral dosing, may be more important. This alone, however, cannot explain the higher efficacy in CAPRISA 004 compared to VOICE.

Adherence is a powerful explanatory variable among oral studies given the enhanced relative risk reduction when drug can be measured in the blood (Partner’s PrEP, iPrEX) and absence of efficacy when concentrations are lower (FEM-PrEP) [5], [8], [26]. If vaginal tissue TFV-DP concentration is the critical site of action for the preventive efficacy of TFV, then adherence alone is very unlikely to explain low (or no) vaginal gel efficacy in light of the 100-fold greater vaginal tissue concentration advantage of vaginal compared to oral dosing. For example, if poor adherence is to explain the lower topical dosing efficacy (VOICE and CAPRISA 004) when compared to Partner’s PrEP and TDF2, adherence would have to be sufficiently worse in the topical dosing studies that vaginal tissue concentrations fall sufficiently far to negate the greater than 100-fold vaginal tissue TFV-DP advantage associated with vaginal dosing and then fall even further to lose most or all of beneficial effects of oral dosing seen in Partners PrEP and TDF2.

To put this adherence difference in temporal terms, consider the half-life of elimination of TFV-DP from vaginal tissue. The terminal half-life of TFV-DP after a single oral dose as measured in vaginal tissue homogenates over two weeks of sampling is estimated at 53 hours [27]. We also estimated a 90 hour half-life using mean vaginal tissue mean TFV-DP data reported in a single oral TDF/FTC dose study [28]. Using these half-life estimates of 53 or 90 hours, the women receiving TFV gel in our study could stop all dosing for more than 2 weeks before their vaginal tissue TFV-DP concentration falls to the concentrations achieved in the oral only dosing arm in the same women [27], [28]. Yet, we see nothing like this kind of low adherence with vaginal dosing – our crude estimates are between 3.2 and 5.2 doses per week.

Therefore, there must be unmeasured variables at work with topical TFV that negate the vaginal concentration-dependent protection of tenofovir demonstrated among oral TFV PrEP studies. Plausible explanations for this protection-negating, possibly HIV-enhancing, effect include tissue toxicity from either concentration-dependent TFV or TFV-DP effects or dose frequency-dependent gel vehicle effects. Although TFV 1% gel has been found to be safe and well tolerated in women, our hypothesized negative effects could be either beneath the level of sensitivity or beyond the scope of the safety measures employed in large clinical trials. To impute non-drug related HIV enhancing effects as the cause of lower than expected efficacy in topical studies – increased frequency of anal intercourse, elevated partner viral load, noxious environmental or para-sexual behaviors, increased prevalence of sexually transmitted infections, higher systemic innate immune activation [29] – requires three conditions. First, the factor(s) must be more prevalent in the poor efficacy topical studies than the high efficacy oral studies. Second, the factor(s) must have a disproportionately high impact on narrowing relative risk reduction in poor efficacy topical PrEP studies compared to high efficacy oral studies. Third, these factors must combine for a greater magnitude of effect than the greater than 100-fold TFV-DP tissue concentration advantage with topical dosing. The tissue concentration differences between oral and vaginal dosing can only partially be mitigated by poor adherence as we argue above. Cross-study analyses of measured variables and smaller hypothesis testing studies to assess unmeasured, but biologically plausible, variables are needed to understand the interaction of pharmacologic with non-pharmacologic variables. Identification of these variables is essential to improving future PrEP development.

Poor adherence in our study could reduce observed concentrations and increase variability. We had clear evidence of poor adherence in many subjects which we detailed in another report (Minnis, *et al*) [10]. However, greater than 60% of subjects had evidence of consistent daily dosing based on comparison of individual drug concentrations at study visits to expected concentrations based on observations in observed dosing studies. Therefore, since we reported medians to summarize our data, these point estimates were largely resistant to the downward adherence bias. In addition, the magnitude of the differences between regimens was so large that the skewed distribution due to adherence bias in the lower tail had little effect.

It is unclear why we detected a correlation between PBMC surface marker with TFV-DP concentration in vaginal tissue, but not PBMC on whose surface the increased activation markers were detected. It is possible that (unmeasured) vaginal tissue activation markers had an even greater correlation with tissue TFV-DP concentration and a modest correlation with PBMC markers, but we did not extract cells from vaginal tissue to test these correlations directly. Three separate *in vitro* studies indicate complex intracellular TFV-DP effects of PHA (+ IL-2) stimulation: no TFV-DP concentration difference in resting versus PHA/IL-2 stimulated PBMCs [30]; 2-fold higher TFV-DP concentration in resting compared to PHA/IL-2 stimulated CEM cells after 6 hours in culture, but no different after 24 hours in culture [31]; 3-fold higher TFV-DP concentration in resting compared to PHA stimulated PBMCs which persisted over 24 hours [32]. In contrast, dATP increases from 3-fold [30] to 10-fold [33] with PHA±IL-2 stimulation which suggests that increased cellular activation would shift the dATP/TFV-DP ratio by modifying the numerator and denominator in directions unfavorable to antiviral effect. In general, the direction of the correlation between cell activation (indicated by CD38 and HLA-DR expression) with decreasing tissue TFV-DP concentration in our clinical study is consistent with 2 of 3 *in vitro* studies.

Limitations to our study were several and point out the difficulties in sampling and comparing findings in these complex and varied anatomic spaces. Due to the destructive sampling (CVL, endocervical cytobrush) or logistical complexity (biopsies), we used a sparse sampling approach for some anatomic and geographic sites. Population PK modeling is underway to best incorporate this data to understand the temporal and spatial relationships of drug in multiple anatomic compartments. The short, variable, and uncertain (self-reported) dosing interval before the clinic dose introduced additional noise in drug concentration assessments, but the impact was minimal for all but serum concentration data due to the relatively long half-life and flat concentration-time profile in other sites. Our CVL samples were based on dilution-adjusted TFV concentrations which bring all of the concentration values closer to their original state. However, this crude adjustment doesn’t account for variations in native cervicovaginal fluid volume and results in significant underestimates in subjects sampled post-gel dose in vaginal dosing periods due to residual TFV gel volume. An internal standard, like lithium [11], [34], [35], to correct for lavage dilution or a validated direct sampling method (syringe, sponge, other adsorbent material) would improve these estimates. Our conversion of intracellular TFV-DP per cell concentrations to molar per volume concentrations helpfully enables comparisons of parent to phosphorylated drug moieties in adjacent spaces or to TFV-DP in different spaces. However, these conversions are not based on our direct assessment of cell volume or type (PBMC subsets, columnar or squamous epithelial cells), but on reasoned assumptions applying previous reports and best guesses. Finally, assessment of TFV-DP in CD4+ T cells extracted from vaginal tissue biopsies would have been preferred over tissue homogenates to provide both a more relevant cell-specific intracellular TFV-DP concentration and to avoid the dilutional effects of extracellular components in tissue homogenates. Nonetheless, if tissue homogenate concentrations are shown to have a systematic relationship to tissue CD4+ T cell TFV-DP, tissue homogenate TFV-DP data will be useful. These limitations warrant cautious application of our comparisons of parent to phosphorylated moiety within a compartment, oral to topical dosing in some compartments (CVL), and comparison between compartments all of which require assumptions based on limited data which leave ample room for future progress.

### Conclusion

In healthy women, daily dosing of tenofovir 1% gel (40 mg) achieves substantially lower (56-fold) systemic exposure of tenofovir compared to daily oral dosing of tenofovir disoproxil fumarate (Viread™). Rectal fluid concentrations of tenofovir were greater after vaginal dosing than oral dosing raising the potential that a vaginal dosing route might provide some level of protection from receptive anal intercourse, though this remains to be studied. When compared to daily oral dosing, vaginal dosing achieved more than 130 times greater vaginal tissue concentrations of active drug (tenofovir diphosphate), leading to the expectation that vaginal TFV dosing should achieve greater protective efficacy than oral TFV dosing, even allowing for very poor adherence. Because this concentration advantage has not been seen in the seroconversion outcomes of two randomized clinical trials (CAPRISA 004 and VOICE), it raises concern that factors beyond TFV’s antiviral effect may substantially reduce PrEP efficacy when dosed topically. This discordance between drug concentration and expected outcome warrants further investigation to better understand TFV and other antiretroviral approaches to topical HIV prevention.

## Supporting Information

Table S1 **Summary of TFV and TFV-DP assay performance characteristics by biological matrix.**
(DOCX)

Protocol S1 **Trial protocol.**
(PDF)

Checklist S1 **CONSORT checklist.**
(DOCX)
