# Supplementary material for: MTN-001: Randomized Pharmacokinetic Cross-Over Study Comparing Tenofovir Vaginal Gel and Oral Tablets in Vaginal Tissue and Other Compartments
Source: PLoS One. 2013 Jan 30;8(1):e55013. doi: 10.1371/journal.pone.0055013 (PMC3559346; doi:10.1371/journal.pone.0055013)
Supplement: Table S1 — Summary of TFV and TFV-DP assay performance characteristics by biological matrix. (DOCX) [file pone.0055013.s001.docx]

Table S1. Summary of TFV and TFV-DP assay performance characteristics by biological matrix

| Analyte | Matrix | Range | LLOQ | | Inter-day | | Intra-day | |
| --- | --- | --- | --- | --- | --- | --- | --- | --- |
|  |  |  | Precision | Accuracy | Precision | Accuracy | Precision | Accuracy |
| TFV | Low Plasma/Serum | 0.31 – 10 ng/mL | 2.8% to 10.5% | ‑6.1% to 5.1% | 4.4% to 8.3% | 5.7% to 8.2% | 2.1% to 10.6% | ‑0.4% to 13.6% |
| TFV | Plasma/Serum | 5 – 1000 ng/mL | 1.7% to 6.9% | ‑1.1% to 9.4% | 4.7% to 5.5% | ‑2.0% to 2.5% | 1.0% to 5.4% | ‑7.6% to 6.4% |
| TFV | Rectal Fluid | 5 – 1280 ng/mL | 3.7% to 10.6% | ‑6.8% to 9.1% | 3.4% to 6.5% | ‑11.7% to 1.6% | 1.1% to 8.8% | ‑13.8% to 4.4% |
| TFV | Tissue | 0.25– 50 ng/sample | 4.3% to 6.6% | ‑6.0% to ‑1.1% | 3.4% to 3.5% | 0.2% to 2.8% | 1.8% to 5.0% | ‑2.9% to 4.6% |
| TFV | Cervicovaginal lavage | 5– 1000 ng/mL | 4.6% to 9.2% | ‑4.2% to 9.4% | 3.5% to 5.2% | 5.2% to 9.8% | 1.7% to 5.9% | 1.0% to 13.0% |
| TFV-DP | Lysate/Tissue | 50– 1500 ng/sample | 2.6% to 7.8% | ‑9.4% to 1.8% | 4.1% to 6.5% | ‑2.1% to 6.8% | 1.7% to 7.3% | ‑8.33% to 10.0% |
