# Supplementary material for: MTN-001: Randomized Pharmacokinetic Cross-Over Study Comparing Tenofovir Vaginal Gel and Oral Tablets in Vaginal Tissue and Other Compartments
Source: PLoS One. 2013 Jan 30;8(1):e55013. doi: 10.1371/journal.pone.0055013 (PMC3559346; doi:10.1371/journal.pone.0055013)
Supplement: Protocol S1 — Trial protocol. (PDF) [file pone.0055013.s002.pdf]

MTN-001

Phase 2 Adherence and Pharmacokinetics Study of Oral and Vaginal Preparations of Tenofovir

A Study of the Microbicide Trials Network

Sponsored by:
Division of AIDS, US National Institute of Allergy and Infectious Diseases
US National Institutes of Health

Grant #:
5-U01-AI068633-03

DAIDS Protocol #10617

Co-Sponsored by:
CONRAD
Gilead Sciences, Inc.

IND# 55,690

Protocol Chair:
Craig W. Hendrix, MD

Version 2.0

03 September 2008

MTN-001

Phase 2 Adherence and Pharmacokinetics Study of Oral and Vaginal Preparations of Tenofovir

TABLE OF CONTENTS

LIST OF ABBREVIATIONS AND ACRONYMS v
PROTOCOL TEAM ROSTER vii
INVESTIGATOR SIGNATURE FORM xiv
PROTOCOL SUMMARY xv
1 KEY ROLES 18
1.1 Protocol Identification 18
1.2 Sponsor and Monitor Identification 18
1.3 Medical Officer 18
1.4 Site Investigators 18
1.5 Clinical Laboratories 19
1.6 Data Center 19
1.7 Study Operations 20
2 INTRODUCTION 20
2.1 Oral Pre-Exposure Prophylaxis and Microbicides in HIV/AIDS Prevention 20
2.2 Tenofovir Disoproxil Fumarate (TDF) 21
2.3 Tenofovir 1% Gel (Tenofovir Gel) 22
2.4 *In Vitro* Studies 23
2.5 Animal Studies 23
2.6 Clinical Studies 26
2.7 Study Hypothesis and Rationale 30
2.8 Justification of Dosing 32
3 OBJECTIVES 33
3.1 Primary Objectives 33
3.2 Secondary Objectives 33
3.3 Exploratory Objectives 33
4 STUDY DESIGN 34
4.1 Identification of Study Design 34
4.2 Summary of Major Endpoints 34
4.3 Description of Study Population 34
4.4 Time to Complete Enrollment 34
4.5 Study Groups 34
4.6 Sequence and Duration of Trial Periods 35
4.7 Expected Duration of Participation 35
4.8 Sites 35
5 STUDY POPULATION 36
5.1 Selection of the Study Population 36
5.2 Inclusion Criteria 36
5.3 Exclusion Criteria 37

5.4 Selection of Participants for In-Depth Interviews ... 39
6 STUDY PRODUCT ... 40
6.1 Regimen ... 40
6.2 Administration ... 40
6.3 Study Product Formulation ... 41
6.4 Study Product Supply and Accountability ... 42
6.5 Participant Counseling ... 44
6.6 Assessment of Participant Adherence ... 44
6.7 Concomitant Medications ... 44
7 STUDY PROCEDURES ... 45
7.1 Screening Visit ... 46
7.2 Enrollment Visit ... 48
7.3 Follow-up Visits ... 49
7.4 Follow up Procedures for Participants who Discontinue Study Product ... 54
7.5 Interim Contacts and Visits ... 55
7.6 Clinical Evaluations and Procedures ... 57
7.7 Behavioral Measures ... 57
7.8 Pharmacokinetic Procedures ... 58
7.9 Laboratory Evaluations ... 62
7.10 Specimen Collection and Processing ... 63
7.11 Specimen Handling ... 63
7.12 Biohazard Containment ... 63
7.13 Final Contact ... 64
8 ASSESSMENT OF SAFETY ... 64
8.1 Safety Monitoring ... 64
8.2 Clinical Data Safety Review ... 64
8.3 Adverse Events Definitions and Reporting Requirements ... 65
8.4 Expedited Adverse Event Reporting Requirements ... 67
8.5 Local Regulatory Requirements ... 69
8.6 Social Harms Reporting ... 69
9 CLINICAL MANAGEMENT ... 69
9.1 Grading System ... 69
9.2 Dose Modification Instructions ... 69
9.3 Discontinuation of Study Product(s) in the Presence of Toxicity ... 70
9.4 General Criteria for Discontinuation of Study Product ... 70
9.5 Management of Specific Toxicities ... 72
9.6 HIV and Hepatitis B ... 75
9.7 Clinical Management of Pregnancy ... 75
9.8 Criteria for Early Termination of Study Participation ... 76
10 STATISTICAL CONSIDERATIONS ... 76
10.1 Overview and General Design ... 76
10.2 Study Endpoints ... 77
10.3 Study Hypotheses ... 77
10.4 Sample Size ... 78
10.5 Randomization Procedures ... 81
10.6 Participant Accrual, Follow-up, Retention, and Replacement ... 81

10.7 Data Monitoring and Analysis ... 82
11 DATA HANDLING AND RECORDKEEPING ... 83
11.1 Data Management Responsibilities ... 83
11.2 Source Documents and Access to Source Data/Documents ... 83
11.3 Quality Control and Quality Assurance ... 83
11.4 Study Coordination ... 83
12 CLINICAL SITE MONITORING ... 83
13 HUMAN SUBJECTS PROTECTIONS ... 84
13.1 Institutional Review Boards ... 84
13.2 Protocol Registration ... 84
13.3 Risk Benefit Statement ... 85
13.4 Informed Consent Process ... 86
13.5 Participant Confidentiality ... 87
13.6 Special Populations ... 88
13.7 Incentives ... 89
13.8 Communicable Disease Reporting ... 89
13.9 Access to HIV-related Care ... 89
13.10 Study Discontinuation ... 90
14 PUBLICATION POLICY ... 90
15 APPENDICES ... 91
APPENDIX I: SCHEDULE OF STUDY VISITS AND EVALUATIONS ... 92
APPENDIX II: HIV ANTIBODY TESTING ALGORITHMS ... 93
APPENDIX III: COMPONENTS OF EXAMINATIONS ... 95
APPENDIX IV: TOXICITY TABLES ... 96
APPENDIX V: MANUAL FOR EXPEDITED REPORTING OF ADVERSE EVENTS TO DAIDS ... 109
APPENDIX VI: SAMPLE INFORMED CONSENT FORM (SCREENING) ... 110
APPENDIX VII: SAMPLE INFORMED CONSENT DOCUMENT (ENROLLMENT) ... 117
APPENDIX VIII: SAMPLE INFORMED CONSENT (STORAGE AND FUTURE TESTING OF SPECIMENS) ... 134
REFERENCES ... 138

TABLE OF FIGURES

Table 1: Study Regimen xvi
Table 2: Tenofovir Bioassay Data 25
Table 3: Study Regimen 40
Table 4: Screening Visit 47
Table 5: Enrollment Visit 48
Table 6: Mid-Study-Period Visit 49
Table 7: End of Study Period Visit 50
Table 8: 7-Week and 14-Week Study Visit 52
Table 9: 21-Week Visit 53
Table 10: Interim Visits and Contacts 56
Table 11: Non-Intensive PK Participants Only 59
Table 12: Intensive PK Participants Only (US site participants only) 61
Table 13: Minimum Detectable Difference in Adherence 79
Table 14: Minimum Detectable Difference in Condom Use 80
Table 15: Study Regimen 81

MTN-001

Phase 2 Adherence and Pharmacokinetics Study of Oral and Vaginal Preparations of Tenofovir

LIST OF ABBREVIATIONS AND ACRONYMS

AE	adverse event
ALT	alanine transaminase
ARV	antiretroviral
AST	aspartate aminotransferase
AUC	area under the curve
BMD	bone mineral density
BV	bacterial vaginosis
CBC	complete blood count
CDC	Centers for Disease Control
CFR	Code of Federal Regulations
cGMP	current Good Manufacturing Practices
C_{max}	maximum serum concentrations
C_{min}	minimum serum concentrations
CONRAD	Contraceptive Research and Development Organization
CRPMC	Clinical Research Products Management Center
CT	Chlamydia trachomatis
CTA	Clinical Trial Agreement
CVL	cervicovaginal lavage
CWG	community working group
DAIDS	Division of AIDS
DNA	deoxyribonucleic acid
EAE	expedited adverse event
EC	Ethics Committee
EC_{50}	50% effective concentration
ELISA	Enzyme Linked Immunosorbent Assay
FDA	(United States) Food and Drug Administration
GCP	Good Clinical Practices
HBsAg	Hepatitis B surface antigen
HCG	human chorionic gonadotropin
HIV	Human Immunodeficiency Virus
HIV-1	Human Immunodeficiency Virus—Type 1
HPTN	HIV Prevention Trials Network
HSV	Herpes Simplex Virus
HSV-2	Herpes Simplex Virus—Type 2
IATA	International Air Transport Association
IND	investigational new drug
IoR	Investigator of Record
IRB	Institutional Review Board
IUD	intrauterine device
LDMS	Laboratory Data Management System
MSM	men who have sex with men
MTN	Microbicide Trials Network
NIAID	National Institute of Allergy and Infectious Disease

NIH	National Institutes of Health
PBMC	peripheral blood mononuclear cell(s)
PK	pharmacokinetic(s)
PMPA	9-[2-(Phosphonomethoxy)propyl]adenine
PoR	Pharmacist of Record
PPD	Pharmaceutical Product Development, Inc
ppts	participants
PrEP	pre-exposure prophylaxis
PSRT	Protocol Safety Review Team
RCC	Regulatory Compliance Center
RNA	ribonucleic acid
RT	reverse transcriptase
RTI	reproductive tract infection
SAE	serious adverse event
SCHARP	Statistical Center for HIV/AIDS Research & Prevention
SD	standard deviation
SDA	Strand Displacement Assay
SDMC	Statistical Data Management Center
SMC	Study Monitoring Committee
SOP	standard operating procedure(s)
SSP	study specific procedure(s)
STI	sexually transmitted infection
TDF	tenofovir disoproxil fumarate
ULN	upper limits of normal
US	United States
VOICE	Vaginal and Oral Interventions to Control the Epidemic

MTN-001

Phase 2 Adherence and Pharmacokinetics Study of Oral and Vaginal Preparations of Tenofovir

PROTOCOL TEAM ROSTER

Protocol Chair

Craig Hendrix, MD
Protocol Chair
John Hopkins University
600 North Wolfe Street
Harvey 502
Baltimore, MD 21287 USA
T: 410-955-9707
F: 410-955-9708
cwhendrix@jhmi.edu

Co-Sponsor Representatives

Henry L. Gabelnick, PhD
Co-Sponsor Representative
CONRAD
Eastern Virginia Medical School
1611 North Kent Street
Arlington, VA 22209 USA
T: 703-524-4744
F: 703-524-4770
hgabelnick@conrad.org

James Rooney, MD
Co-Sponsor Representative
Gilead Sciences, Inc.
333 Lakeside Drive
Foster City, CA 94404 USA
T: 650-522-5708
F: 650-522-5854
Jim_Rooney@gilead.com

Jill Schwartz, MD
Co-Sponsor Representative
CONRAD
1611 North Kent Street, Suite 806
Arlington, VA 22209 USA
T: 703-276-3913
F: 703-524-4770
jschwartz@conrad.org

Division of AIDS (DAIDS)

Roberta Black, PhD
Microbicides Research Branch Chief
Division of AIDS, NIAID
6700 B Rockledge Drive, Room 5135
Bethesda, MD 20817 USA
T: 301-496-8199
F: 301-402-3684
rblack@niaid.nih.gov

Debra S. Mérès, PharmD
DAIDS Protocol Pharmacist
Pharmaceutical Affairs Branch
Division of AIDS, NIAID
6700-B Rockledge Dr., Ste 4222
Bethesda, MD 20892 USA
T: 301-451-2775
F: 301-402-1506
depayne@niaid.nih.gov

Jeanna Piper, MD
DAIDS Senior Medical Officer
Division of AIDS, NIAID
National Institutes of Health
6700 B Rockledge Drive, Room 5248
Bethesda, MD 20892 USA
T: 301-451-2778
F: 301-402-3684
piperj@niaid.nih.gov

Lydia E. Soto-Torres, MD, MPH
DAIDS Medical Officer
NIAID/DAIDS
6700 B Rockledge Drive, Room 5138
Bethesda, MD 20892-7628 USA
T: 301-594-9705
F: 301-402-3684
lsoto-torres@niaid.nih.gov

Family Health International (FHI)

Kailazarid Gomez, MPM
Clinical Research Manager
Family Health International
2224 E NC Highway 54
Durham, NC 27713 USA
T: 919-544-7040 Ext. 11282
F: 919-544-0207
kgomez@fhi.org

Sherri Johnson
Prevention Research Specialist
Family Health International
4401 Wilson Boulevard
Suite 700
Arlington, VA 22203 USA
T: 703 516-9779 Ext. 12127
F: 703 516-0295
sjohnson@fhi.org

Lisa Levy, MPH
Prevention Research Specialist
Family Health International
PO Box 13950
Research Triangle Park, NC 27709 USA
T: 919-544-7040 Ext. 11260
F: 919-544-0207
llevy@fhi.org

Rhonda White, R.H. Ed
Community Program Manager
Family Health International
P.O. Box 13950
Research Triangle Park, NC 27709 USA
T: 919-544-7040 Ext. 11515
F: 919-544-0207
rwhite@fhi.org

Investigators

Mitchell D. Creinin, MD
Co-Investigator
University of Pittsburgh
Dept. of OB/GYN/RS
Magee-Womens Hospital
300 Halket Street
Pittsburgh, PA 15213 USA
T: 412-641-1441
F: 412-641-1133
mcreinin@mail.magee.edu

Roshini Govinden, PhD
Co-Investigator
South African Medical Research Council
123 Jan Hofmeyer Road
Westville, Durban, KwaZulu-Natal 3630 South Africa
T: 27-31-242-3632
F: 27-31-242-3800
Roshini.Govinden@mrc.ac.za

Craig Hoesley, MD
Co-Investigator
University of Alabama at Birmingham
1530 3rd Avenue South
BDB 467 Box 20
Birmingham, AL 35294 USA
T: 205-934-7090
F: 205-975-2563
choesley@uab.edu

Jessica Justman, MD
Co-Investigator
Center for Infectious Disease Epidemiologic Research,
Mailman School of Public Health
Columbia University
722 West 168th Street, Room 714
New York, NY 10032 USA
T: 212-342-0537
F: 212-342-1824
jj2158@columbia.edu

Michael Lederman, MD
CTU Principal Investigator
Case Western Reserve University
2061 Cornel
Foley Building
Cleveland, OH 44106 USA
T: 216-844-8786
F: 216-844-5523
lederman.michael@clevelandactu.org

Clemensia Nakabiito, MBChB, MMed
Co-Investigator
MU-JHU Research Collaboration
PO Box 23491
Kampala, Uganda
T: 256-41-541044/ 256-772-405332 (cell)
F: 256-41-541044/256-41-532091
cnakabiito@mujhu.org

Gita Ramjee, MSc, PhD
Co-Investigator
South African Medical Research Council
HIV Prevention Research Unit
P.O. Box 70380
Overport Durban KwaZulu-Natal 4067 South Africa
T: 27-31-242-3631
F: 27-31-242-3806
ramjeeg@mrc.ac.za

Sharon A. Riddler, MD, MPH
CRS Principal Investigator
University of Pittsburgh
Falk Medical Building, Suite 611
3601 Fifth Avenue
Pittsburgh, PA 15213 USA
T: 412-647-6710
F: 412-647-5519
riddler@dom.pitt.edu

Robert A. Salata, MD
Co-Investigator
Case Western Reserve University
Department of Medicine
11100 Euclid Avenue
Cleveland, OH 44106 USA
T: 216-844-3287
F: 216-844-1632
robert.salata@case.edu

Microbicide Trials Network (MTN)

Katherine Bunge, MD
MTN Safety Physician
Microbicide Trials Network
MWH of UPMC
Department of OB/GYN/RS
300 Halket St.
Pittsburgh, PA 15213 USA
T: 412-917-9936 (pager)
F: 412-641-6170
kbunge@mail.magee.edu

Nancy Connolly, MD
MTN Safety Physician
Microbicide Trials Network
10001 35th Ave., NE
Seattle, WA 98125 USA
T: 206-523-1177
nancycsc@gmail.com

Ross D. Cranston MD FRCP
MTN Safety Physician
Division of Infectious Disease
University of Pittsburgh Medical Center
Falk Medical Building, Suite 611
3601 Fifth Avenue
Pittsburgh, PA 15213 USA
T: 412-647-4007
F: 412-647-5519
cranstonr@dom.pitt.edu

Charlene S. Dezzutti, PhD
MTN Network Laboratory Director
Microbicide Trials Network
204 Craft Avenue
Pittsburgh, PA 15213 USA
T: 412-641-3462
F: 412-641-6170

dezzuttics@upmc.edu

Betsy Herold, MD
MTN Biomedical Science Committee Representative
Albert Einstein College of Medicine
1300 Morris Park Avenue, Forchheimer 702
Bronx, NY 10463 USA
T: 718-430-2974
T: 718-430-2975 (Lab)
F: 718-430-8627
bherold@aecom.yu.edu

Sharon Hillier, PhD
MTN Principal Investigator
Microbicide Trials Network
204 Craft Avenue
Pittsburgh, PA 15213 USA
T: 412-641-8933
F: 412-641-6170
shillier@mail.magee.edu

Cindy Jacobson, PharmD
MTN Research Pharmacist
Microbicide Trials Network
Magee-Womens Research Institute
204 Craft Avenue
Pittsburgh, PA 15213 USA
T: 412-641-8913
F: 412-641-6170
cjacobson@mail.magee.edu

Edward Livant, BSMT (ASCP), MPH
MTN Research Manager
Microbicide Trials Network
204 Craft Avenue, Room A534
Pittsburgh, PA 15213 USA
T: 412-641-3772
F: 412-641-5290
livantew@upmc.edu

Ian McGowan, MD, PhD, FRCP
MTN Co-Principal Investigator
Microbicide Trials Network
204 Craft Avenue
Pittsburgh, PA 15213 USA
T: 412-641-8999
F: 412-641-6170
imcgowan@pitt.edu

Alexandra Minnis, PhD, MPH
Co-Investigator
San Francisco Office for RTI
114 Sansome Street, Suite 500
San Francisco, CA 94104-3812 USA
T: 415-848-1323
F: 415-848-1330
aminnis@rti.org

Patrick Ndase, MBChB, MPH
MTN Regional Physician
Plot 131 Makindye Road
Kampala, Uganda
T: 256-753-080-489
F: 256-41-532091
pndase@u.washington.edu

Lisa Noguchi, CNM, MSN
Director of Operations
Microbicide Trials Network
204 Craft Avenue
Pittsburgh, PA 15213 USA
T: 412-641-8945
F: 412-641-6170
lnoguchi@mail.magee.edu

Nancy Padian, PhD
MTN Behavioral Research Committee Representative
San Francisco Office for RTI
114 Sansome Street, Suite 500
San Francisco, CA 94104-3812 USA
T: 415-848-1331
F: 415-848-1330
npadian@rti.org

Mala Shah, MPH
MTN Protocol Development Manager
Microbicide Trials Network
204 Craft Avenue
Pittsburgh, PA 15213 USA
T: 412-641-3859
F: 412-641-6170
shahms@mail.magee.edu

Microbicide Trials Network (MTN) Community Working Group (CWG)

Robert Bucklew, JD
MTN CWG Representative
CWRU/UH AIDS Clinical Trials Unit
Center for AIDS Research
2061 Cornell Road, #312
Mail Stop: 5083
Cleveland, OH 44106-5083 USA
T: 216-844-2247
F: 216-844-3926
bucklew.robert@clevelandactu.org

Statistical Data Management Center (SDMC)

Corey Kelly, MPH
MTN SDMC Project Manager
FHCRC—SCHARP
1100 Fairview Avenue North, LE-400
PO Box 19024
Seattle, WA 98109-1024 USA
T: 206-667-5170
F: 206-667-4812
ckelly@scharp.org

Karen Patterson, MPH
SDMC Project Manager
FHCRC—SCHARP
1100 Fairview Avenue N., LE-500
P.O. Box 19024
Seattle, WA 98109-1024 USA
T: 206-667-7052
F: 206-667-4812
karen@scharp.org

Barbra Richardson, PhD
SDMC Biostatistician
1100 Fairview Avenue North
LE-400
Seattle, WA 98109-1024 USA
T: 206-731-2425 (T, W, TH)
T: 206-667-7788 (M, F)
F: 206-667-4812
Barbra@scharp.org

MTN-001

Phase 2 Adherence and Pharmacokinetics Study of Oral and Vaginal Preparations of Tenofovir

INVESTIGATOR SIGNATURE FORM

Version 2.0
03 September 2008

A Study of the Microbicide Trials Network (MTN)

Sponsored by:
Division of AIDS, US National Institute of Allergy and Infectious Diseases
US National Institutes of Health

Co-Sponsored by:
CONRAD
Gilead Sciences, Inc.

I, the Investigator of Record, agree to conduct this study in full accordance with the provisions of this protocol. I will comply with all requirements regarding the obligations of investigators as outlined in the Statement of Investigator (Form FDA 1572), which I have also signed. I agree to maintain all study documentation for at least two years following the date of marketing approval for the study gel and/or tablets for the indication in which it was/they were studied. If no marketing application is filed, or if the application is not approved, the records will be retained for two years after the investigation is discontinued and the US Food and Drug Administration is notified. Publication of the results of this study will be governed by MTN policies. Any presentation, abstract, or manuscript will be submitted to the MTN Manuscript Review Committee, DAIDS, CONRAD, and Gilead Sciences, Inc. for review prior to submission.

I have read and understand the information in the Investigator's Brochure(s) and Package Insert(s), including the potential risks and side effects of the products under investigation, and will ensure that all associates, colleagues, and employees assisting in the conduct of the study are informed about the obligations incurred by their contribution to the study.

____
Name of Investigator of Record

______________________________ ______________________________
Signature of Investigator of Record Date

MTN-001

Phase 2 Adherence and Pharmacokinetics Study of Oral and Vaginal Preparations of Tenofovir

PROTOCOL SUMMARY

Short Title: Adherence and Pharmacokinetics Study of Oral and Vaginal Preparations of Tenofovir

Clinical Phase: 2

IND Sponsor: DAIDS (for both oral TDF 300 mg tablet and tenofovir 1% vaginal gel)

Protocol Chair: Craig W. Hendrix, MD

Sample Size: Approximately 144 evaluable participants (including 72 for intensive pharmacokinetic sub-study at domestic sites)

Study Population: Sexually active, HIV-uninfected women between the ages of 18 and 45 years

Participating Clinical Research Sites (CRS):

- Umkomaas CRS, Durban, KwaZulu-Natal, South Africa
- Botha's Hill CRS, Durban, KwaZulu-Natal, South Africa
- Makerere University - JHU Research Collaboration {MUJHU CARE LTD} CRS, Kampala, Uganda
- Alabama Microbicide CRS, Birmingham, USA
- Bronx-Lebanon Hospital Center CRS, Bronx, USA
- Case CRS, Cleveland, USA
- Pitt CRS, Pittsburgh, USA

Study Design: Phase 2, multi-site, randomized, six sequence, three period, open label crossover study

Study Duration: Approximately 21 weeks per participant, with projected six calendar months of accrual

Study Regimen:

Table 1: Study Regimen

	N	Period 1: 6 WEEKS	1 WK Wash-out	Period 2: 6 WEEKS	1 WK Wash-out	Period 3: 6 WEEKS	1 WK Wash-out
Sequence A	24	Oral		Vaginal		Oral + Vaginal	
Sequence B	24	Vaginal		Oral		Oral + Vaginal	
Sequence C	24	Oral + Vaginal		Oral		Vaginal	
Sequence D	24	Oral + Vaginal		Vaginal		Oral	
Sequence E	24	Oral		Oral + Vaginal		Vaginal	
Sequence F	24	Vaginal		Oral + Vaginal		Oral	

Primary Objectives:

- To compare adherence to and acceptability of three daily regimens of tenofovir (oral, vaginal, and dual use)
- To compare systemic and local pharmacokinetics (PK) among three regimens of tenofovir (oral, vaginal, and dual use) in a subset of participants

Primary Endpoints:

- **Adherence.** Participant self reported product use. For each woman, adherence to each regimen will be computed by dividing the number of daily doses she reports having taken (numerator) by the number of expected doses if she were fully adherent (denominator).
- **Acceptability**. The proportion of participants who indicate that they would be "unlikely" to use the study product in the future.
- **PK**. Area under the curve (AUC), maximum serum concentrations ($C_{max)}$, and minimum serum concentrations (C_{min}) associated with oral, vaginal, and dual use regimens.

Secondary Objectives:

- To identify factors associated with product adherence, and whether these differ when women use one of three daily regimens of tenofovir (oral, vaginal, and dual use)
- To examine whether sexual activity or male condom use varies when women use one of three daily regimens of tenofovir (oral, vaginal, and dual use)
- To assess the timing of product use with sexual intercourse
- To determine the level of sharing of study products with non-participants (and to assess with whom products are shared)

- To characterize the differential safety profiles of three daily regimens of tenofovir (oral, vaginal, and dual use)

Secondary Endpoints

- Proportion of women who report taking at least 90% of expected daily doses, frequency of use during the follow-up interval using an ordinal measure (5 categories of use, never to always); number of days product missed or not used during the previous week

- Frequency (ordinal measures) of sexual activity and male condom use

- Time interval between product usage and sexual intercourse; sequence of product use and sexual intercourse

- Reported sharing of study product; quantity of shared study product

- Grade 3 or higher toxicity for systemic and local effects as defined by DAIDS Adverse Event (AE) Grading Table Version 1.0, December 2004, or Grade 3 or higher genital infection, pain or epithelial lesion as defined by the Female Genital Grading Table for Use in Microbicide Studies which cannot be directly attributed to another cause, and judged as definitely, probably, possibly, or probably not related to the study gel, applicator, or study tablet

Exploratory Objectives

- To build a PK model of intracellular-extracellular tenofovir levels in the systemic and female genital tract compartments

- To examine the impact of oral tenofovir and vaginal tenofovir gel on mucosal immunity in the female genital tract

- To assess correlation of PK and adherence measures

Exploratory Endpoints

- PK measures (blood, intracellular, and tissue values for C_{min}, C_{max}, and AUC)

- Intrinsic antimicrobial activity and mediators of mucosal immunity at Enrollment and at the end of each study period

- Adherence measures as outlined above for the primary and secondary objectives

1 KEY ROLES

1.1 Protocol Identification

Protocol Title:	Phase 2 Adherence and Pharmacokinetics Study of Oral and Vaginal Preparations of Tenofovir
MTN Protocol Number:	MTN-001
Date:	03 September 2008

1.2 Sponsor and Monitor Identification

Sponsor:	DAIDS/National Institute of Allergy and Infectious Diseases (NIAID)/National Institutes of Health (NIH)<br>6700 B Rockledge Drive<br>Bethesda, MD 20892 USA
Co-Sponsor:	CONRAD<br>Eastern Virginia Medical School<br>1611 North Kent Street, Suite 806<br>Arlington, VA 22209 USA
Co-Sponsor:	Gilead Sciences, Inc.<br>333 Lakeside Drive<br>Foster City, CA 94404 USA
Monitor:	Pharmaceutical Product Development, Inc. (PPD)<br>929 North Front Street<br>Wilmington, NC 28401-3331 USA

1.3 Medical Officer

**DAIDS Medical Officer:**	Lydia E. Soto-Torres, MD, MPH<br>National Institute of Allergy and Infectious Diseases<br>Division of AIDS<br>6700 B Rockledge Drive, Room 5138<br>Bethesda, MD 20892 USA

1.4 Site Investigators

Site Investigator:	Clemensia Nakabiito, MBChB, MMed<br>Makerere University-Johns Hopkins University<br>Uganda

Site Investigator:	Roshini Govinden, PhD<br>Umkomaas CRS and Botha’s Hill CRS<br>Medical Research Council<br>South Africa
Site Investigator:	Mitchell D. Creinin, MD<br>University of Pittsburgh<br>USA
Site Investigator:	Robert Salata, MD<br>Case Western Reserve University<br>USA
Site Investigator:	Craig Hoesley, MD<br>University of Alabama at Birmingham<br>USA
Site Investigator:	Jessica Justman, MD<br>Bronx-Lebanon Hospital Center<br>USA

1.5 Clinical Laboratories

Network Laboratory:	MTN Network Laboratory (NL)<br>Magee-Womens Research Institute<br>204 Craft Avenue<br>Pittsburgh, PA 15213 USA
Pharmacology:	MTN NL Pharmacology CORE<br>Osler 527<br>600 N. Wolfe St.<br>Johns Hopkins University<br>Baltimore, MD 21287 USA
Herold Laboratory:	Albert Einstein College of Medicine<br>1300 Morris Park Avenue, Forchheimer 702<br>Bronx, NY 10463 USA

1.6 Data Center

Data Center:	Statistical Center for HIV/AIDS Research & Prevention (SCHARP)<br>Fred Hutchinson Cancer Research Center<br>1100 Fairview Avenue N., LE-400<br>PO Box 19024<br>Seattle, WA 98109-1024 USA

1.7 Study Operations

Study Operations: Family Health International
PO Box 13950
Research Triangle Park, NC 27709 USA

2 INTRODUCTION

2.1 Oral Pre-Exposure Prophylaxis and Microbicides in HIV/AIDS Prevention

More than twenty years after the first diagnosis of human immunodeficiency virus (HIV) was made, the epidemic continues to grow at an alarming rate. While recent developments in treatment have made dramatic improvements in life expectancy and quality of life for persons living with HIV and Acquired Immune Deficiency Syndrome (AIDS), these regimens are not cures, and remain inaccessible for much of the world. The XVI AIDS Conference highlighted these issues, as well as ongoing efforts to develop new strategies for the prevention of HIV transmission, particularly pre-exposure prophylaxis (PrEP) and microbicides.

Pre-exposure prophylaxis refers to the use of oral antiretroviral (ARV) medication to prevent HIV infection. Due to encouraging evidence from recent animal studies, scientific interest in PrEP continues, despite the closure of studies of PrEP using tenofovir in Cambodia and Cameroon due to local pressure. Pre-exposure prophylaxis has been controversial for several reasons. There is a concern about the cost of the regimen, especially in developing nations where cost-effective prevention strategies are needed most. More concerning, in particular for the HIV/AIDS activist community, is the perception that widespread use of PrEP could lead to problems with toxicity, ARV resistance, and behavioral disinhibition. The urgent need for new prevention strategies and the many unanswered questions surrounding PrEP call for further study on its potential as a safe, effective, and acceptable means of HIV prevention. The ideal PrEP drug would be safe, effective, have no or limited impact on HIV drug resistance, easy to use, and cost-effective. Such a product is yet to be identified.

Microbicides are being developed as substances intended to reduce or prevent transmission of HIV and/or other sexually transmitted infections (STIs) when applied topically to genital mucosal surfaces. A significant body of *in vitro*, animal, and preliminary clinical data suggests that tenofovir holds promise as a safe and effective vaginal microbicide. However, a potential weakness for single-compound ARV microbicides, such as tenofovir, is drug resistance. Exposure to HIV resistant to the ARV in a microbicide might result in protection levels that are less than those perceived by the user. It remains unclear whether drug resistance (and associated limitations in future treatment options) could occur with a microbicide containing a single ARV that is not significantly systemically absorbed. It has been posited that drug levels would likely be too low to select for drug resistant virus, but there are currently no data on this.

To address many of the unanswered questions, the MTN proposes to conduct a large scale safety and effectiveness study of both oral and vaginal tenofovir, MTN-003, the Vaginal and Oral Interventions to Control the Epidemic (VOICE) Study. Tenofovir will be studied because of its favorable toxicity and resistance profile, demonstrated efficacy against HIV-1 infection in some animal studies, and relatively rapid development path for use as a vaginal microbicide. The comparison of oral vs. vaginal tenofovir is being undertaken because each approach carries specific theoretical and operational advantages: while vaginal use may confer less systemic toxicity, oral use is less closely linked to sexual practices, and possibly could be administered by the woman without knowledge of her partner.

There are theoretical reasons to favor either approach for efficacy and/or selection of resistance, and only a head-to-head trial of these two approaches will begin to answer these questions. However, before a large efficacy trial comparing oral and vaginal preparations is undertaken, it is prudent to estimate the rates of adherence for both oral and vaginal regimens of tenofovir. While not strictly a feasibility study, MTN-001 will provide data on adherence to and PK of both oral and vaginal regimens of tenofovir that will inform the design of a large Phase 2B trial to compare the potential effectiveness of these two prevention strategies.

The behavioral factors associated with adherence to different prevention strategies are still being described. The full complement of HIV prevention strategies in the future arguably could include both orally and vaginally formulated agents. Different strategies will likely appeal to different risk populations, or even to the same woman at different times in her life. A woman’s adherence to a particular prevention strategy would impact its effectiveness, as well as its effect on her health. Different levels of adherence to PrEP or ARV-based vaginal microbicide use could also have variable effects on the development of drug-resistant virus in a woman unknowingly infected with HIV. Thus, understanding how and why women are willing or able to commit to a particular formulation is vital to the development of safe and effective drug-based prevention strategies.

2.2 Tenofovir Disoproxil Fumarate (TDF)

2.2.1 Description

Tenofovir disoproxil fumarate is currently approved under the trade name Viread® for the treatment of HIV-1 infection in adults.[1] Tenofovir disoproxil fumarate is the oral pro-drug of tenofovir, an acyclic nucleotide analog (9-R-2-phosphonomethoxypropyl adenine, PMPA) with activity *in vitro* against retroviruses, including HIV-1 and HIV-2, as well as hepadnaviruses. Further information on TDF is available in the current version of the Viread® Package Insert.

2.2.2 Mechanism of Action

Once absorbed, TDF is rapidly converted by diester hydrolysis to tenofovir. Tenofovir is phosphorylated by cellular enzymes to tenofovir diphosphate, which is a competitive inhibitor of HIV-1 reverse transcriptase (RT) that terminates the growing deoxyribonucleic acid (DNA) chain.

2.2.3 Strength of Study Product

The strength of the TDF tablets will be the dose approved by the FDA for the indication of treatment of HIV-1 infection in adults (300 mg). For the treatment of HIV-1 infection, TDF is administered once daily as one 300 mg tablet and has excellent activity against wild type and many drug resistant viruses.

2.3 Tenofovir 1% Gel (Tenofovir Gel)

2.3.1 Description

Tenofovir gel contains 1 gm/100 mL of 9-[2-(Phosphonomethoxy)propyl]adenine (PMPA), an acyclic nucleotide analogue with activity against retroviruses, including HIV-1 and HIV-2, as well as hepadnaviruses.[2] Further information is available in the current version of the tenofovir gel investigator's brochure.

2.3.2 Mechanism of Action

Tenofovir is an acyclic nucleotide analogue of adenosine monophosphate. Tenofovir requires subsequent phosphorylations by cellular enzymes to form tenofovir diphosphate (PMPApp). Tenofovir diphosphate inhibits the activity of HIV-1 reverse transcriptase by competing with the natural substrate deoxyadenosine 5'-triphosphate and, after incorporation into DNA, by DNA chain termination. Tenofovir diphosphate is a weak inhibitor of mammalian DNA polymerases α, β, and mitochondrial DNA polymerase γ.

2.3.3 Strength of Study Product

The strength of tenofovir gel will be the dose (1%) previously tested in HPTN 050/IND 55,690, CONRAD A04-095/IND 73,382, CONRAD A04-099/IND 73,382, HPTN 059/IND 55,690, and MTN-002/IND 55,690. The application proposed in this study will deliver approximately 40 mg of tenofovir to the vaginal compartment.

2.4 *In Vitro* Studies

Anti-HIV-1 Activity

The *in vitro* antiviral activity of tenofovir against laboratory and clinical isolates of HIV-1 was assessed in lymphoblastoid cell lines, primary monocyte/macrophage cells and peripheral blood lymphocytes.[1] The EC_{50} (50% effective concentration) values for tenofovir were in the range of 0.04 μM to 8.5 μM. In drug combination studies of tenofovir with nucleoside reverse transcriptase inhibitors (abacavir, didanosine, lamivudine, zalcitabine, zidovudine), additive synergistic effects were observed. Tenofovir displayed antiviral activity *in vitro* against HIV-1 clades A, B, C, D, E, F, G, and O (EC_{50} values ranged from 0.5 μM to 2.2 μM) and strain specific activity against HIV-2 (EC_{50} values ranged from 1.6 μM to 4.9 μM).

2.5 Animal Studies

2.5.1 Tenofovir and Tenofovir Disoproxil Fumarate

Toxicology

Tenofovir and TDF administered in toxicology studies to rats, dogs, and monkeys at exposures (based on AUCs) greater than or equal to 6 fold those observed in humans caused bone toxicity.[1] In monkeys the bone toxicity was diagnosed as osteomalacia. Osteomalacia observed in monkeys appeared to be reversible upon dose reduction or discontinuation of tenofovir. In rats and dogs, the bone toxicity manifested as reduced bone mineral density. The mechanism(s) underlying bone toxicity is unknown.

Four gravid rhesus monkeys were administered tenofovir subcutaneously once daily from 20 to 150 days of gestation (30 mg/kg; term: 165 ± 10 days).[3] Fetuses were monitored sonographically, and maternal and fetal blood and urine samples were collected to assess hematologic parameters, clinical chemistry, insulin-like growth factor (IGF) levels, and bone biomarkers. Fetuses were delivered by hysterotomy near term for necropsy and evaluation of bone-related mechanical properties. Results of these studies showed 1) normal fetal development, although overall body weights and crown-rump lengths were less than those for age-matched controls ($p \leq .03$); 2) a significant reduction in circulating IGF-I ($p < .001$); 3) a small reduction in fetal bone porosity ($p \leq .03$); and 4) transient alterations in maternal body weights and bone-related biomarkers during treatment. Results of these studies suggest that chronic fetal exposure to subcutaneous tenofovir at the maternal dose of 30 mg/kg throughout gestation can alter select fetal parameters and transiently affect maternal bone biomarkers.

Evidence of renal toxicity was noted in 4 animal species.[1] Increases in serum creatinine, blood urea nitrogen, glycosuria, proteinuria, phosphaturia, and/or calciuria and decreases in serum phosphate were observed to varying degrees in these animals. These toxicities were noted at exposures (based on AUCs) 2–20 times higher than those observed in humans. The relationship of the renal abnormalities, particularly the phosphaturia, to the bone toxicity is not known.

Carcinogenesis and Mutagenesis

Long-term oral carcinogenicity studies of TDF in mice and rats were carried out at exposures up to approximately 16 times (mice) and 5 times (rats) those observed in humans at the therapeutic dose for HIV infection.[1] At the high dose in female mice, liver adenomas were increased at exposures 16 times that observed in humans. In rats, the study was negative for carcinogenic findings at exposures up to 5 times that observed in humans at the therapeutic dose. Tenofovir disoproxil fumarate was mutagenic in the *in vitro* mouse lymphoma assay, but negative in an *in vitro* bacterial mutagenicity test (Ames test). In an *in vivo* mouse micronucleus assay, tenofovir disoproxil fumarate was negative when administered to male mice.

Reproductive Toxicity

There were no effects on fertility, mating performance or early embryonic development when TDF was administered to male rats at a dose equivalent to 10 times the human dose based on body surface area comparisons for 28 days prior to mating and to female rats for 15 days prior to mating through day seven of gestation.[1] There was, however, an alteration of the estrous cycle in female rats. Reproduction studies have been performed in rats and rabbits at doses up to 14 and 19 times the human dose based on body surface area comparisons and revealed no evidence of impaired fertility or harm to the fetus due to tenofovir.

2.5.2 Tenofovir 1% Gel

Pharmacokinetics

Single-dose PK in female rabbits has been previously examined (0.5 mL, 1% weight/volume (w/v) tenofovir, 5 mg per animal, 50 µCi/kg).[2] Plasma concentrations of radioactivity were highest at the first sample time point (0.5 hr) and below the level of quantification at 24 hours. Pharmacokinetic parameters including the proportion of dose absorbed systemically could not be estimated, due to the very low plasma concentrations.

In a tissue distribution study using the same tenofovir vaginal gel formulation, dose and strength as the above study, eighteen female rabbits were administered an intravaginal dose using a gavage needle.[2] An additional eighteen rabbits received an intravaginal dose of 3% w/v tenofovir (15 mg per animal). Analysis of vaginal tissue sections found no clear relationship between tissue concentration and dose, with no consistent pattern of distribution. Very little radioactivity was recovered in non-vaginal tissues. Concentrations in blood (0.002 to 0.047 µg-eq/g of tissue) exemplified the variability of distribution of the product.

The PK, excretion and tissue distribution of ^{14}C-PMPA were evaluated in rats following intravaginal administration of an earlier formulation of tenofovir gel containing propylene glycol.[4] Four female rats received a single intravaginal dose administered as an aqueous gel containing 20 mg tenofovir/g. Plasma concentrations of total radioactivity were highly variable; this was attributed to inconsistent retention of the formulation

within the vagina. The apparent C_{max} for tenofovir occurred at the earliest time point (15 minute), suggesting that absorption from the vagina was relatively rapid. Thereafter plasma concentrations declined with an approximate half-life of 1.6 hours. The bioavailability of intravaginal tenofovir was estimated by comparison of the observed $AUC_{(0-24)}$ with historical AUC data for an intravenous dose of 10 mg/kg tenofovir in rats (9.71 µg hr/mL). The observed systemic bioavailability of intravaginal tenofovir was 7.9%.

In the excretion and distribution study, two groups of four additional rats received a single intravaginal dose of ^{14}C-PMPA (approximately 10 mg/kg, 100 µCi/kg) administered as an aqueous gel containing 20 mg tenofovir/g. This study found that much of the dose was lost from the vaginal orifice by leakage. Vaginal tissue contained 0.1% of the dose and less than 0.01% of the dose was recovered in the ovaries and uterus.

The PK of radiolabeled tenofovir was evaluated via plasma and vaginal biopsies collected from four rhesus monkeys following single-dose intravaginal administration of tenofovir 1% vaginal gel.[2] Radioactivity was detected starting at 15 minutes post application, with peak concentration of tenofovir in tissue at 8 hours and remaining high at 12 hours. No significant radioactivity was detected in whole blood or plasma.

The systemic and vaginal tissue bioavailability was assessed in female white New Zealand rabbits following single and multiple intravaginal doses (twice a day for 7 or 14 days) of 1mL of 1% tenofovir gel or a single intravenous (IV) solution of 10mg tenofovir.[2] Vaginal tissue was rinsed and samples collected at either 4 or 8 hours post-dose. System absorption following a single intravaginal dose was barely detectable, and only within the first 30 minutes. Multiple intravaginal administrations of tenofovir 1% gel and the single IV administration of 10mg tenofovir resulted in systemic levels of tenofovir (see Table 2).

Table 2: Tenofovir Bioassay Data

	**Mean 1st Rinse Vaginal Surface (ng/mL)**	**Mean Vaginal Tissue Concentration (ng/gm)**	**C_{max} (ng/mL)**	**AUC (0-4 hr) (ng*hr/mL)**
Single IV, 8 hr	**362** (19-990)	**950** (120-5,019)	**10,221**	**4,013** (3,192-4,503)
Single vaginal, 8 hr	**97** (7-415)	**940** (10-7,277)	**3**	--
Single vaginal, 4 hr	**1,441** (2-5,100)	**2,817** (35-11,780)	**5**	--
Twice daily x 7d vaginal, 4 hr	**1,086** (145-4,369)	**3,146** (448-14,429)	**239** (29-808)	**342** (54-1,037)
Twice daily x 14d vaginal, 4 hr	**3,361** (33-8,000)	**11,409** (245-50, 102)	**71** (24-197)	**94** (12-229)

Toxicology
The preclinical toxicity of tenofovir gel has been evaluated in 14-day rat and 10-day rabbit vaginal irritation and toxicity studies.[5,6] Daily intravaginal administration of tenofovir gel produced no vaginal irritation in rats (≤10% tenofovir) and minimal to mild vaginal irritation in rabbits (3% or 10% tenofovir).

2.6 Clinical Studies

2.6.1 Tenofovir Disoproxil Fumarate 300 mg Tablet

Additional safety information from clinical studies on the TDF 300 mg tablet is available in the Viread® package insert.[1] Tenofovir disoproxil fumarate PK have been evaluated in healthy volunteers and HIV-1 infected individuals. Tenofovir PK are similar between these populations.

Pharmacokinetics
Tenofovir disoproxil fumarate is a water soluble diester prodrug of the active ingredient tenofovir. The oral bioavailability of tenofovir from TDF in fasted patients is approximately 25%. Following oral administration of a single dose of TDF 300 mg to HIV-1 infected patients in the fasted state, C_{max} are achieved in 1.0 ± 0.4 hrs. Maximum serum concentration and AUC values are 296 ± 90 ng/mL and 2287 ± 685 ng·hr/mL, respectively. The PK of tenofovir are dose proportional over a TDF dose range of 75 to 600 mg and are not affected by repeated dosing. Tenofovir PK are similar in male and female patients.

Safety
Gilead Study 903, a randomized, double-blind trial conducted in the United States, Europe and South America, was designed to compare the efficacy and safety of a treatment regimen of TDF, lamivudine (3TC) and efavirenz to a regimen of stavudine (d4T), lamivudine and efavirenz in 600 antiretroviral-naïve HIV-1 infected patients. In Gilead Study 903 through 144 weeks of study treatment, decreases from baseline in bone mineral density (BMD) were seen at the lumbar spine and hip in both arms of the study. At Week 144, there was a significantly greater mean percentage decrease from baseline in BMD at the lumbar spine in patients receiving TDF + lamivudine + efavirenz (-2.2% ± 3.9) compared with patients receiving stavudine + lamivudine + efavirenz (-1.0% ± 4.6). Changes in BMD at the hip were similar between the two treatment groups (-2.8% ± 3.5 in the TDF group vs. -2.4% ± 4.5 in the stavudine group). In both groups, the majority of the reduction in BMD occurred in the first 24–48 weeks of the study and this reduction was sustained through Week 144. Twenty-eight percent of TDF-treated patients vs. 21% of the stavudine treated patients lost at least 5% of BMD at the spine or 7% of BMD at the hip. Clinically relevant fractures (excluding fingers and toes) were reported in 4 patients in the TDF group and 6 patients in the stavudine group. In addition, there were significant increases in biochemical markers of bone metabolism (serum bone specific alkaline phosphatase, serum osteocalcin, serum C-telopeptide, and urinary N-telopeptide) in the TDF group relative to the stavudine group, suggesting increased bone turnover. Serum parathyroid hormone levels and 1.25 Vitamin D levels

were also higher in the TDF group. Except for bone specific alkaline phosphatase, these changes resulted in values that remained within the normal range. The effects of TDF-associated changes in BMD and biochemical markers on long-term bone health and future fracture risk are unknown.

Tenofovir is eliminated by renal elimination, including tubular secretion. Thus, dose-interval adjustments are necessary for TDF in patients with significant renal impairment. TDF-induced nephrotoxicity has been reported, especially in patients with other medical problems or pre-existing renal dysfunction.

The most common (occurring in 2% or more of recipients) treatment-emergent adverse events in the Gilead 903 study in HIV-infection treatment naïve adults receiving TDF + lamivudine + efavirenz included whole body (headache, pain, fever, abdominal pain, back pain, asthenia), gastrointestinal (diarrhea, nausea, dyspepsia, vomiting), musculoskeletal (arthralgia, myalgia), nervous system (depression, insomnia, dizziness, anxiety), respiratory (pneumonia), and skin rash. The most frequent laboratory abnormalities were elevations in fasting cholesterol, creatine kinase, amylase, or aspartate aminotransferase (AST) or alanine transaminase (ALT), hematuria, and decreased absolute neutrophil count. The frequency of all these events and laboratory abnormalities was similar or lower in the TDF treated group compared to the stavudine treated group.

A Phase 1, 17-day, open label, two-way crossover, randomized PK study to evaluate the relative bioavailability and bioequivalence between a single dose of TDF oral powder and tablet formulation was conducted in healthy male and female participants.[1]. The primary objective of this study was to determine the relative bioavailability between the investigational oral powder and the 300 mg tablet formulations of TDF. A total of 32 healthy adult participants (including non-pregnant, non-lactating females) were recruited at a single US site and received TDF in the study.

Preliminary results of this completed study indicate that TDF administered as the 300 mg tablet or oral powder formulation was well tolerated. No participant discontinued the study because of an adverse event. All treatment-emergent adverse events after administration of the oral powder and tablet were Grade 1 in severity. No serious adverse events or deaths occurred. Two participants discontinued the study by withdrawing consent. Treatment-emergent AEs were reported in 12 of 30 participants (40%, 18 events) after administration of the oral powder compared with 15 of 32 participants (47%, 29 events) after administration of the tablet. Headache, experienced by 4 participants (13%), was the most frequently reported treatment-emergent AE after the oral powder. The most frequently reported treatment-emergent AE after administration of the tablet were headache (6 participants, 19%) and dizziness (4 participants, 13%). Treatment-emergent AEs considered by the investigator to be related to study drug included flatulence (2 participants, 7 %), hot flush (1 participant, 3%) and increased ALT (1 participant, 2%); all related AEs were Grade I in severity and resolved without therapy. No AEs were considered related to study drug after administration of the tablet.

Peterson, et al. evaluated the safety of TDF 300 mg daily versus placebo for prevention of HIV-1 infection in women in a Phase 2 double-blind study conducted at 3 sites in West Africa.[7] The study closed prematurely resulting in insufficient power to evaluate efficacy. In the primary safety analysis, with 428 person-years (p-y) of follow up, there was no significant difference in the rate of safety endpoints (defined as grade 2 or higher serum creatinine, grade 3 or 4 transaminase elevation, or grade 3 or 4 phosphate abnormality). Among the 368 participants on TDF, none had grade 3 or 4 transaminase elevation or grade 2 or higher creatinine. One TDF recipient had self-limited grade 3 phosphate.

Several other studies examining the safety and effectiveness of oral tenofovir as PrEP are underway or in later stages of planning. These include the following:

- The Bangkok Tenofovir Study (safety and efficacy of daily tenofovir to prevent parenteral HIV infection among injection drug users, CDC)
- Botswana TDF/FTC Oral HIV Prophylaxis Trial (Safety and Efficacy of Daily Oral Antiretroviral Use for the Prevention of HIV Infection in Heterosexually Active Young Adults in Botswana, CDC)
- Extended Safety Study of Tenofovir Disoproxil Fumarate (TDF) Among HIV-1 Negative Men (clinical and behavioral safety and tolerability of oral daily TDF use as pre-exposure prophylaxis (PrEP) to prevent HIV infection in uninfected men, CDC)
- Chemoprophylaxis for HIV Prevention in Men (IND# 71,859)
- FHI 2/Phase III daily Truvada or daily oral placebo for prevention of HIV-1 infection
- Parallel Comparison of Tenofovir and Emtricitabine/Tenofovir Pre-Exposure Prophylaxis to Prevent HIV-1 Acquisition within HIV-1 Discordant Couples (IND #75,365)
- Safety and Effectiveness of Tenofovir Disoproxil Fumarate (TDF) in high-risk, West African women (FHI)

2.6.2 Tenofovir 1% Gel

Pharmacokinetics

A Phase 1 Safety and Acceptability Study of the Vaginal Microbicide Agent PMPA Gel, also known as HPTN 050, is a recently completed study of tenofovir vaginal gel with published data.[8] Eighty-four (60 HIV negative and 24 HIV positive) women applied

either 0.3% or 1% tenofovir gel once or twice daily for 14 days. Systemic absorption was limited (maximum serum levels 3.1-25.8 ng/mL).

Safety

In HPTN 050, the tenofovir 1% gel formulation was well tolerated in both HIV negative and HIV positive women. Although 92% reported at least 1 AE, the majority (87%) were mild and limited to the genitourinary tract (77%). Product concentration, sexual activity and HIV status were not associated with a specific AE pattern. No clinically significant systemic toxicity was observed. Tenofovir gel showed no negative effect on vaginal microflora in this study. No new resistance mutations evolved in plasma or cervicovaginal lavage (CVL) after 14 days of tenofovir gel use but 3 women had plasma mutations associated with low level tenofovir resistance at day 0 and 14 (M41L, L210M, ±T215I/Y). The AE and safety profile in HPTN 050 was reviewed by the FDA who subsequently permitted the initiation of the HPTN 059 extended safety protocol for vaginal tenofovir. Tenofovir gel was highly acceptable to both women and men in HPTN 050, with 94% of the women and 81% of the men indicating that they would definitely or probably use tenofovir gel in the future.

In a male tolerance study of tenofovir 1% gel (CONRAD A04-099) (IND #73,382), tenofovir gel was well tolerated in men following seven days of once daily exposure, for 6 to 10 hours, to the penis. There were few reported and observed genital findings after product use including mild pain (burning, irritation, discomfort) and pruritis[2]. All observed findings were classified as mild, were small in size and required no treatment. All reported symptoms were mild, of short duration and resolved by the final visit. There were no noticeable differences between signs and symptoms of genital irritation observed in the circumcised compared to the uncircumcised group.[2,9]

A Phase 2 study of tenofovir 1% gel (HPTN 059) has completed follow up. This study assessed safety and acceptability of, and adherence to a regimen of tenofovir gel for vaginal use in HIV-uninfected women versus a placebo gel. Exploratory objectives included measurement of vaginal flora characteristics, assessment of the effects of gel on genital cytokine and chemokine expression, and the evaluation of cytokine and chemokine expression to correlate expression with evidence of inflammation, epithelial disruption and genital symptoms. The study was a Phase 2 four arm, three site, randomized, controlled trial comparing gel used once daily and gel used prior to intercourse, to placebo gel, with 6 months gel exposure and follow-up. The study was conducted among 200 women in Pune, India; Birmingham, Alabama, USA; and New York, New York, USA. Participants were sexually active, HIV-uninfected women between ages 18 and 50, but not menopausal or post menopausal. Participants had six months of study gel exposure and six months of follow-up. They were randomized to either once daily or coitally dependent group, and received either tenofovir or placebo gel. Participants received single use unit dose tubes and single use applicators.

No statistically significant differences were seen between those receiving active and placebo gels in complete blood count, liver function tests, or renal function tests. Among those using a study gel daily, no participants had pelvic exam findings involving

generalized erythema or severe edema or deep epithelial disruption at any follow-up visit during the study. At the Week 24 Visit, no participants had exam findings suggestive of vaginitis, cervicitis, superficial disruption, disrupted blood vessels, or intermenstrual bleeding. Adherence to study gel was high, and was supported by PK data. 79% of women reporting gel use in past 12 hours had low but detectable plasma tenofovir supporting self-reported adherence data. Daily and coital use was highly acceptable to women. These data suggest a favorable safety and acceptability profile of tenofovir gel, and support routine monitoring for genital findings among women without genital symptoms at six month intervals.[10]

Several other tenofovir gel studies are ongoing or in development. These include:

- CONRAD A04-095: Single Dose and 14-Day Once or Twice-daily Pharmacokinetic Study of the Vaginal Microbicide Agent 1% Tenofovir Gel (IND #73,382)

- CAPRISA 004: Safety and Effectiveness Study of a Candidate Vaginal Microbicide for Prevention of HIV (proposed Phase 2B, two-arm, double-blinded, randomized, placebo controlled trial comparing 1% tenofovir gel with a placebo gel)

- MTN-002: Phase 1 Study of the Maternal Single-Dose Pharmacokinetics and Placental Transfer of Tenofovir 1% Vaginal Gel among Healthy Term Gravidas

- MTN-003: Phase 2B Safety and Effectiveness Study of Tenofovir 1% Gel, Tenofovir Disoproxil Fumarate (TDF) Tablet and TDF-Emtricitabine Tablet for the Prevention of HIV-1 Infection in Women

- MTN-006: Phase 1 Rectal PK Study of Tenofovir 1% Gel

- MTN-007: Phase 1 Rectal Safety of Tenofovir 1% Gel

2.7 Study Hypothesis and Rationale

2.7.1 Study Hypothesis

MTN-001 hypothesizes that:

- There will be no differences in rates of adherence among the three study regimens

- There will be no difference in rates of acceptability among the three study regimens

- Tissue levels of PMPA will be similar irrespective of the route of administration

- Oral TDF will be associated with higher concentrations of PMPA in the blood compared to topical administration of PMPA

- Neither tenofovir 1% gel nor oral TDF regimens will adversely impact the genital tract environment

2.7.2 Rationale

Adherence

The microbicide field will benefit from evaluating the behavioral factors associated with adherence to oral and vaginal formulations. The full complement of HIV prevention strategies in the future may include orally or vaginally formulated agents or combinations of both. Accordingly, efficacy trials should also include regimens of oral, vaginal, or both types of formulations. Different strategies will likely appeal to different risk populations, or even to the same woman at different times in her life. A woman's level of adherence to a particular prevention modality would impact its effectiveness, depending on her use pattern and the drug's half-life, as well as its effect (if any) on her health. It has also been posited that different levels of adherence to PrEP or ARV-based vaginal microbicide use could have variable effects on the development of drug-resistant virus in a woman unknowingly infected with HIV.

Understanding how product formulation affects adherence and acceptability as well as sexual risk taking (particularly condom use) is important to the design of the MTN-003 trial. Including adherence and acceptability assessments in MTN-001 permits the opportunity to pilot measures that would be used in MTN-003. Behavioral adherence data could complement interpretation of PK results.

Pharmacokinetics

No head to head comparison controlling for intra-individual variability has yet been performed to compare the relative efficiency of oral versus vaginal tenofovir dosing to achieve measurable and "adequate" (biologically active based on *in vitro* data) tenofovir levels in tissue. MTN-001 will evaluate intracellular tenofovir diphosphate concentrations (active moiety) in tissue and blood to inform PK models.

Based on data from HPTN 050, only one-third of the participants are expected to yield measurable levels in blood after vaginal dosing of tenofovir gel. An intensive PK sampling cohort is planned to provide frequent blood samples and carefully timed corresponding cervicovaginal lavage (CVL) and vaginal biopsy, one per woman, at pre-specified times after dosing to provide data for a sparse sampling analysis. For this reason and for reasons of technical capacity, all participants at the United States (US) sites will enter the Intensive PK substudy.

2.8 Justification of Dosing

Tenofovir 1% Vaginal Gel

Choice of the tenofovir 1% vaginal gel concentration for MTN-001 is based on both animal and clinical evidence suggesting an appropriate safety profile and potency. Animal and human studies have demonstrated minimal vaginal irritation at this concentration. A rabbit vaginal irritation test identified tenofovir 1% gel as being histopathologically identical to sham or control treatment, while on a qualitative basis 3% gel was more irritating to vaginal epithelia.[2] The tolerability of the 1% gel was confirmed in the HPTN 050 Phase 1 study, the Phase 1 dose ranging study of tenofovir gel (0.3% once daily, then 1.0% once daily, then 0.3% twice daily followed by 1% twice daily).[2,Error! Bookmark not defined.] In this study, of the two doses and frequencies studied in the dose finding cohort, the 1% gel applied intravaginally twice daily for 14 days was well tolerated and was identified as the highest practical dose and frequency for further study in subsequent cohorts.

The second line of evidence is from vaginal transmission inhibition studies performed in non-human primates.[2] Six separate studies provided evidence for efficacy of the gel over a range of tenofovir concentrations of 1% to 10%. Although the total data are limited and a powered statistical determination as to the efficacy of tenofovir 1% gel versus 0.3% and 10% cannot be made, empirical examination of the efficacy data identifies tenofovir 1% gel as the lowest efficacious concentration tested when given within two hours of infection.

Finally, limited vaginal PK tenofovir data in primates demonstrate that tenofovir gel is broadly distributed in vaginal tissues following vaginal application and can penetrate to epithelial tissues.[11] The amount of tenofovir administered by intravaginal application of 4 grams of a 1% dose (40 mg) is highly active against HIV and results in a reduction of plasma HIV ribonucleic acid (RNA) of 1.5 $\log_{10}$ copies / mL after daily administration for 21 days. Comparison of the predicted cervicovaginal concentrations of tenofovir gel delivered to those achieved systemically at the standard treatment dose of 300 mg TDF, and tenofovir's characteristic prolonged intracellular half-life (diphosphate form, nine to 50 hours depending upon cell type), suggest that an initial and potentially durational barrier to HIV transmission may be possible. In terms of weighing potential risks and benefits, the tenofovir 1% gel minimizes the potential risks of vaginal epithelial toxicity while providing the potential benefit of delivering sufficient tenofovir to achieve an initial and possibly durational barrier to infection.

Tenofovir Disoproxil Fumarate 300 mg Tablets

Choice of the 300 mg strength of the TDF tablet is based upon practical and scientific considerations. The TDF 300 mg tablet, or Viread®, is the medication US FDA approved for the indication of treatment of HIV-1 infection. More than 12,000 people have been treated with TDF alone or in combination with other antiretroviral medications for periods of 28 days to 215 weeks in Phase 1–3 clinical trials and expanded access studies. A total of 1,544 patients have received TDF 300 mg once daily in Phase 1–3 clinical trials and over 11,000 people have received TDF in expanded access studies. A

significant body of safety data has been accumulated for daily use of the TDF 300 mg tablet. In addition, data on tenofovir PK and anti-viral activity in humans suggest a reasonable expectation of effectiveness as a prevention strategy.

3 OBJECTIVES

3.1 Primary Objectives

- To compare adherence to and acceptability of three daily regimens of tenofovir (oral, vaginal, and dual use)
- To compare systemic and local PK among three regimens of tenofovir (oral, vaginal, and dual use) in a subset of participants

3.2 Secondary Objectives

- To identify factors associated with product adherence, and whether these differ when women use one of three daily regimens of tenofovir (oral, vaginal, and dual use)
- To examine whether sexual activity or male condom use varies when women use one of three daily regimens of tenofovir (oral, vaginal, and dual use)
- To assess the timing of product use with sexual intercourse
- To determine the level of sharing of study products with non-participants (and to assess with whom products are shared)
- To characterize the differential safety profiles of three daily regimens of tenofovir (oral, vaginal, and dual use)

3.3 Exploratory Objectives

- To build a PK model of intracellular-extracellular tenofovir levels in the systemic and female genital tract compartments
- To examine the impact of oral and vaginal tenofovir gel on mucosal immunity in the female genital tract
- To assess correlation of PK and adherence measures

4 STUDY DESIGN

4.1 Identification of Study Design

MTN-001 will be a Phase 2, multi-site, randomized, six sequence, three period open label crossover study of adherence to and PK of TDF 300 mg tablet and tenofovir 1% gel.

4.2 Summary of Major Endpoints

- Participant self-reported product use. For each woman, adherence to each regimen will be computed by dividing the number of daily doses she reports having taken (numerator) by the number of expected doses if she were fully adherent (denominator).

- The proportion of participants who indicate they would be "unlikely" to use the study product in the future.

- AUC, C_{max}, and C_{min} associated with oral, vaginal, and dual use regimens.

4.3 Description of Study Population

The study population will include approximately 144 evaluable generally healthy 18-45 year-old women who are HIV-uninfected, non-pregnant, sexually active and using adequate contraception, as described in Section 5.2. Among the total approximately 144 evaluable participants, the 72 participants enrolled at all US study sites will undergo more intensive specimen collection for PK analysis. These participants are hereafter referred to as “Intensive PK participants.”

4.4 Time to Complete Enrollment

The approximate time to complete study enrollment is expected to be six months. The time of total study duration is expected to be a minimum of approximately one year, including the study follow-up period.

4.5 Study Groups

Six study sequences are planned. The 144 planned participants will be randomized equally across all 6 sequences in a 1:1:1:1:1:1 ratio. All study participants will be assigned to complete each study period (oral, gel, oral plus gel) once in an order that will be randomly assigned. Up to 204 women may be enrolled to reach the target of 144 evaluable women (i.e., women who have at least one follow-up visit with adherence data in each of the three periods of the study). The need to enroll replacement participants for enrollees will be dependent on whether the enrollee had at least one full period of the study with no follow-up adherence data. Details on procedures for

participant replacement are outlined in the MTN-001 Study Specific Procedures (SSP) Manual (www.mtnstopshiv.org).

4.6 Sequence and Duration of Trial Periods

The total duration of participation from the Enrollment Visit to the Termination Visit is 21 weeks, including three six-week study periods and three one-week washout periods. Visits may be completed within specified windows around target dates. Detailed information regarding visit windows will be thoroughly described in the MTN-001 SSP Manual.

4.7 Expected Duration of Participation

The expected duration of participation for individual enrolled participants is 21 weeks. No study data will be collected after the 21-Week Termination Visit unless the participant has an AE that has not resolved or stabilized, or is pregnant at the Termination Visit, or is a participant in the in-depth interview with applicable characteristics described in Section 7.6.

Participants who have AEs at the Termination Visit that have not resolved or stabilized will be followed beyond the Termination Visit until a clinically acceptable resolution of the AE(s) is confirmed and documented. Clinical acceptability of resolution will be determined by the site IoR in consultation with the Protocol Safety Review Team (PSRT). For participants who are pregnant at the Termination Visit, study site staff will make every effort to follow the participant until such time that her pregnancy outcome can be ascertained and documented.

4.8 Sites

Seven study sites are planned for this trial:

- Umkomaas CRS, Durban, KwaZulu-Natal, South Africa
- Botha's Hill CRS, Durban, KwaZulu-Natal, South Africa
- Makerere University - JHU Research Collaboration {MUJHU CARE LTD} CRS, Kampala, Uganda
- Alabama Microbicide CRS, Birmingham, USA
- Bronx-Lebanon Hospital Center, Bronx, USA
- Case CRS, Cleveland, USA
- Pitt CRS, Pittsburgh, USA

5 STUDY POPULATION

5.1 Selection of the Study Population

The inclusion and exclusion criteria outlined in Sections 5.2 and 5.3 will be utilized to ensure the appropriate selection of study participants for MTN-001.

5.1.1 Recruitment

Participants will be recruited from family planning and other health clinics, colleges, and other venues. Site IRB-approved media advertisements, telephone scripts, and fliers may be used.

5.1.2 Retention

Each site will establish participant retention procedures. Study site staff members at each site are responsible for developing and implementing site-specific standard operating procedures (SOPs) to target high rates of retention. Further information on retention of participants is provided in Section 10.6.

5.1.3 Enrollment Guidelines

Women with participation in any other investigational drug or device trial in the 30 days prior to enrollment will not be enrolled in this study. Study participants will be required to refrain from enrollment in other clinical trials involving investigational or prohibited drugs or investigational devices during their involvement in this study. Participants who report after enrollment their concurrent participation in such trials will be discussed by the PSRT and may be discontinued from use of study product(s). In this case they will be encouraged to remain in the study and will be followed with all safety evaluations deemed clinically appropriate by the Investigator and the NIH medical officer.

5.2 Inclusion Criteria

Women must meet all of the following criteria to be eligible for inclusion in the study:

1) Age 18-45 years (inclusive) at screening, verified per site SOP

2) Willing and able to provide written informed consent for screening and enrollment

3) Willing and able to provide adequate locator information, as defined in site standard operating procedures

4) HIV-uninfected based on testing performed by study staff during screening procedures (per applicable algorithm in Appendix II)

5) In general good health at screening and enrollment, as determined by the site IoR or designee

6) Per participant report at screening, usual menstrual cycle with at least 21 days between menses (does *not* apply to participants who report using a progestin-only method of contraception at screening, e.g., Depo-Provera)

7) Calculated creatinine clearance at least 70 mL/min by the Cockcroft-Gault formula where creatinine clearance (female) in mL/min = (140 - age in years) x (weight in kg) x 0.85/72 x (serum creatinine in mg/dL)

8) Per participant report at screening, sexually active, defined as having had penile-vaginal intercourse at least four times in the four weeks prior to screening

9) Per participant report at screening and enrollment, intending to continue penile-vaginal intercourse at least once per week for the duration of study participation

10) Per participant report at enrollment, use of an effective method of contraception at enrollment, and intending to use same method for the duration of study participation and one month thereafter; effective methods include hormonal methods (except vaginal ring); intrauterine contraceptive device (IUCD) inserted at least 30 days prior to enrollment; and sterilization (of participant or her sexual partner or partners as applicable, self-report acceptable)

11) Normal Pap smear result at screening or adequately documented normal Pap smear result per SSP within the 12 calendar months prior to screening

12) At screening and enrollment, agrees not to participate in other research studies involving drugs, medical devices, or vaginal products for the duration of study participation

5.3 Exclusion Criteria

Women who meet any of the following criteria will be excluded from the study:

1) Participant reported history of:

   a) Known adverse reaction to either of the study products (ever)

   b) Known adverse reaction to latex (ever)

   c) Any current male sex partner with known history of adverse reaction to latex (ever)

   d) More than three partners in the month prior to screening

e) Pathologic bone fracture not related to trauma

f) Last pregnancy outcome within 90 days or less prior to enrollment

g) Gynecologic or genital procedure (e.g., biopsy, tubal ligation, dilation and curettage) 90 days or less prior to enrollment

h) Participation in any other research study involving drugs, medical devices, or vaginal products 30 days or less prior to enrollment

i) Non-therapeutic injection drug use in the 12 months prior to Screening

j) As determined by the site investigator, any significant uncontrolled active or chronic cardiovascular, renal, liver, hematologic, neurologic, gastrointestinal, psychiatric, endocrine, respiratory, immunologic disorder or infectious disease, including active tuberculosis, or any other medical condition that could make participation unsafe

2) Pregnant at screening or enrollment, or per participant report intending to become pregnant during the period of study participation

3) Per participant report, breastfeeding at screening or enrollment

4) At screening:

a) any liver function test result greater than 1.5 X the site laboratory ULN (upper limit of normal)

b) serum creatinine greater than the site laboratory ULN for women

c) hemoglobin less than 10.0 g/dl

d) platelet count less than 100,000/mm^3

e) serum phosphate level below site laboratory LLN (lower limit of normal)

f) positive for hepatitis B surface antigen (HBsAg)

g) 2+ or greater dipstick urinalysis results for protein

5) At screening or enrollment, unwilling to comply with study participation requirements, including attendance at all scheduled study visits

6) At screening or enrollment,

a) Has a clinically apparent pelvic exam finding (observed by study staff) involving Grade 2 or higher genital lesions, erythema, and/or edema (grading per the Female Genital Grading Table for Use in Microbicide Studies)

b) Has any other abnormal physical or pelvic exam finding that, in the opinion of the investigator or designee, would contraindicate study participation

7) At screening, is diagnosed with RTI requiring treatment per current WHO guidelines or UTI. Reproductive tract infections requiring treatment include symptomatic bacterial vaginosis (BV), symptomatic vaginal candidiasis, other vaginitis, trichomoniasis, chlamydia, gonorrhea, syphilis, active HSV lesions, chancroid, pelvic inflammatory disease, genital sores or ulcers, cervicitis, genital warts of the labia minora, vagina, or cervix, or any other symptomatic genital warts. Presence of genital warts exterior to labia minora requiring treatment is also exclusionary. Note that HSV-2 seropositive with no active lesions is allowed (since treatment is not required).

   If diagnosed at screening: otherwise eligible participants with any of the above RTIs/conditions requiring treatment and/or UTI may be enrolled after completing treatment and all symptoms have resolved, as long as treatment is completed and all symptoms have resolved within 30 days of obtaining informed consent for screening.

8) Per participant report, use of the following at enrollment, and/or anticipated use during the period of study participation:

   a) use of a diaphragm, vaginal ring, and/or spermicide for contraception

   b) acyclovir or valacyclovir

   c) post-exposure prophylaxis for HIV exposure

   d) TDF/emtricitabine

   e) non-study vaginal products

9) At screening or enrollment, has any social or medical condition that, in the investigator's opinion, would preclude informed consent, make study participation unsafe, complicate interpretation of study outcome data, or otherwise interfere with achieving the study objectives

5.4 Selection of Participants for In-Depth Interviews

A subset of participants will be eligible for participation in an in-depth interview regarding their experiences using the study products. As outlined in Section 7.7, a random sample of women will be invited back for an in-depth interview before the study

activities are completed. Data on acceptability and factors affecting adherence will be collected during these in-depth interviews. As one of the purposes of collecting data during the in-depth interview is to facilitate the examination of variance in participant responses noted among different data collection modalities, responses during in-depth interviews need not be reconciled with data collected in other study measures.

6 STUDY PRODUCT

6.1 Regimen

Study participants will be randomized to one of the six study regimen sequences (see Table 2). Each study sequence will consist of three study periods and three wash out periods, for a total duration of 21 weeks. The study period duration is six weeks. A one week wash out period will immediately follow each six-week study period.

Study participants will receive the study products which are tenofovir 1% gel and tenofovir disoproxil fumarate 300 mg tablet. The daily regimen for each six week study period will consist of either single formulation of tenofovir 1% gel or tenofovir disoproxil fumarate 300 mg tablet. The dual formulation use will consist of tenofovir 1% gel and tenofovir disoproxil fumarate 300 mg tablet. All participants will be instructed to complete each study period, which will include the vaginal use of tenofovir 1% gel, the oral use of tenofovir disoproxil fumarate 300 mg tablet, and the dual use of both, in the order designated by their randomized sequence.

Table 3: Study Regimen

	N	Period 1: 6 WEEKS	1 WK Wash-out	Period 2: 6 WEEKS	1 WK Wash-out	Period 3: 6 WEEKS	1 WK Wash -out
Sequence A	24	Oral		Vaginal		Oral + Vaginal	
Sequence B	24	Vaginal		Oral		Oral + Vaginal	
Sequence C	24	Oral + Vaginal		Oral		Vaginal	
Sequence D	24	Oral + Vaginal		Vaginal		Oral	
Sequence E	24	Oral		Oral + Vaginal		Vaginal	
Sequence F	24	Vaginal		Oral + Vaginal		Oral	

6.2 Administration

Tenofovir 1% Gel (Single Formulation Period)

Study participants will be instructed to insert one dose (the entire contents of one applicator) of tenofovir 1% gel into the vagina once daily for the 6-week study period. Vaginal administration of study product should occur before bedtime, usually in the evening, or the longest period of rest.

If a participant misses a dose, she must insert vaginally the missed dose as soon as possible, unless the next dose is estimated to be due within 6 hours. If the next dose is

estimated to be due within 6 hours, the missed dose must be skipped. The next dose will be inserted vaginally as originally scheduled.

Tenofovir Disoproxil Fumarate 300 mg Oral Tablet (Single Formulation Period)

Study participants will be instructed to take one tenofovir disoproxil fumarate 300 mg tablet, by mouth, once daily for the 6-week study period. Tenofovir disoproxil fumarate 300 mg should be taken at bedtime, usually in the evening, or the longest period of rest, without regard to meals. If a participant misses a dose, she must take the missed dose, by mouth, as soon as possible, unless the next dose is estimated to be due within 6 hours. If the next dose is estimated to be due within 6 hours, the missed dose must be skipped. The next dose must be taken, by mouth, as originally scheduled.

Tenofovir 1% Gel and Tenofovir Disoproxil Fumarate 300 mg Oral Tablet Period (Dual Formulation Period)

Study participants will be instructed to insert one dose (the entire contents of one applicator) of tenofovir 1% gel into the vagina and take one tenofovir disoproxil fumarate 300 mg tablet by mouth once daily for the 6-week study period. Both vaginal and oral study products should be administered approximately at the same time, before bedtime, usually in the evening, or the longest period of rest.

If a participant misses a dose of tenofovir 1% gel, she must vaginally insert the missed tenofovir 1% gel as soon as possible unless the next dose is estimated to be due within 6 hours. If a participant misses a tenofovir disoproxil fumarate tablet dose, she must take the missed dose, by mouth, as soon as possible, unless the next dose is estimated to be due within 6 hours. If either dose is estimated to be due within 6 hours, the missed dose should be skipped. The next dose must be taken as originally scheduled.

6.3 Study Product Formulation

6.3.1 Tenofovir 1% Gel

Tenofovir 1% gel (weight/weight) is a gel formulation of tenofovir (PMPA, 9-[(R)-2-(phosphonomethoxy)propyl]adenine monohydrate), formulated in purified water with edetate disodium, citric acid, glycerin, methylparaben, propylparaben, hydroxyethylcellulose, and pH adjusted to 4-5. Tenofovir 1% gel is a transparent, viscous gel that will be filled into applicators to form pre-filled, single-use applicators. Each pre-filled applicator will contain a dose of approximately 4 grams (equal to 4 mL) of tenofovir 1% gel for delivery. Tenofovir 1% gel must be stored at controlled room temperature, 25°C (77°F), at all times. Excursions are permitted between 15°C and 30°C (59°F and 86°F).

6.3.2 Tenofovir Disoproxil Fumarate 300 mg Tablets

Tenofovir disoproxil fumarate (Viread®, TDF) oral tablet, is a fumaric acid salt of bis-isopropoxycarbonyloxymethyl ester derivative of tenofovir. Each tablet contains 300 mg of tenofovir disoproxil fumarate, which is equivalent to 245 mg of tenofovir disoproxil. Tenofovir disoproxil fumarate tablets should be stored and dispensed in the original container. Each bottle should contain a silica gel desiccant to protect the product from humidity, and this should remain in the container. Tenofovir disoproxil fumarate should be stored at 25°C (77°F). Excursions are permitted between 15°C and 30°C (59°F and 86°F).

6.4 Study Product Supply and Accountability

6.4.1 Study Product Supply

Tenofovir 1% Gel
Tenofovir 1% gel will be supplied by CONRAD (Arlington, VA, USA). Under direction from CONRAD, DPT Laboratories, Ltd (San Antonio, TX, USA) which is a contract manufacturing facility, will manufacture the tenofovir 1% gel, and analyze/release tenofovir 1% gel under current good manufacturing practices (cGMP). DPT Laboratories Ltd will fill the applicators with tenofovir 1% gel to create pre-filled applicators and package each applicator and plunger in a wrapper. DPT Laboratories will over-wrap the pre-filled applicators and plungers. There will be 14 wrapped applicators and plungers packed in each carton. The cartons will be shipped under a temperature controlled carrier to the NIAID Clinical Research Products Management Center (CRPMC).

Tenofovir Disoproxil Fumarate 300 mg Tablets
Tenofovir disoproxil fumarate 300 mg tablets will be supplied by Gilead Sciences, Inc. (Foster City, CA, USA). Each bottle of tenofovir disoproxil fumarate will contain 30 tablets. The tablets will be shipped under a temperature controlled carrier to the CRPMC.

Tenofovir 1% Gel and Tenofovir Disoproxil Fumarate 300 mg Tablets
Study products supplied by CONRAD and Gilead Sciences, Inc. will be available through the CRPMC. The Pharmacist of Record can obtain the study products for this protocol by following the instructions in the *Pharmacy Guidelines and Instructions for DAIDS Clinical Trials Networks* in the section Study Product Control.

6.4.2 Dispensing

Study products are dispensed only to enrolled participants, upon receipt of a written prescription from an authorized prescriber. Depending on the study period in which the participant is currently enrolled, single formulation or dual formulation, she will receive either a bottle of 30 tenofovir disoproxil fumarate tablets, 28 pre-filled applicators, or 30

tenofovir disoproxil fumarate tablets and 28 pre-filled applicators (two cartons of 14 applicators), at each regularly scheduled study visit, except the End of Study Period Visits, and the Termination Visit. See Section 7 Study Procedures, for study visit schedule.

6.4.2.1 Pharmacokinetic Visit

As described in Section 7.8 Pharmacokinetic Procedures, all participants will have one timed PK measure at the end of each period of study product administration. These PK measures will occur at the end of the study period visit during their vaginal gel period, the oral period and during the combined oral and vaginal gel period. Participants will be instructed to take the tenofovir disoproxil fumarate tablet by mouth, insert the tenofovir 1% gel, or both, the evening before the end of the study period clinic visit as throughout the study period.

Participants will be instructed to return any unused study product to the clinic at the end of the study period visit. One of the doses the participant brings to this visit will be administered for the timed PK measures. This will be the final dose of study product for that study period. The dose for the timed PK measure will be administered in the clinic.

6.4.2.2 Male Condoms and Panty Liners

All participants will receive male condoms and be offered panty liners. The condoms and panty liners will be dispensed by the clinic staff, and made available in the clinic.

6.4.3 Accountability

Each site PoR is required to maintain complete records of all study products received from the CRPMC and subsequently dispensed. All unused study products must be returned to the CRPMC (or as otherwise directed by the sponsor) after the study is completed or terminated. The procedures to be followed are provided in the manual *Pharmacy Guidelines and Instructions for DAIDS Clinical Trials Networks* in the section Study Product Control.

6.4.4 Retrieval of Unused Study Products

Study participants will be instructed to return all unused study products to the site at each scheduled study visit. In the event that unused study products are not returned at the end of each study period visit, study staff members will make every effort to encourage participants to return study product as soon as possible. Participants who are permanently discontinued from study product use will be instructed to return all

unused study product to the site. The PoR will store returned unused study products in designated areas within the study pharmacy.

6.5 Participant Counseling

Adherence counseling will be provided to study participants upon enrollment into the study, and every visit thereafter to help ensure high rates of study product use. Condom counseling will be provided to all study participants. Counseling will be provided in accordance with standard study methods that will address such topics as participant-centered strategies to remember to use the study gel and/or tablet daily (depending on study period) and to ensure the availability of the study product both in the home and away from home. Counseling also will include reminders to contact study staff with questions about study product use and requests for additional supplies. For participants who have adherence problems, every effort will be made to identify adherence strategies to increase their rates of study product use throughout the course of the study. Participants will be counseled to avoid douching as this will alter exposure to study gel.

Participants also will be instructed to:

- Only use the study gel vaginally
- Not insert vaginal products other than study gel and tampons during menstruation
- Not use other participants' study products
- Not distribute their study products to other people

Participant behaviors regarding condom and study gel use data will be collected via standardized questions developed by the Protocol Team in conjunction with study site staff and community representatives, to maximize the accuracy of self-reported data.

6.6 Assessment of Participant Adherence

Assessment of participant adherence will be addressed using a quantitative instrument and in-depth qualitative interviews (conducted with a subset of study participants).

6.7 Concomitant Medications

With the exception of medications listed as prohibited, enrolled study participants may use concomitant medications during study participation. All concomitant medications reported throughout the course of the study will be recorded on case report forms designated for that purpose. Prescription medications, over-the-counter preparations, vitamins and nutritional supplements, recreational drugs, and herbal preparations will all be recorded on forms for concomitant medications.

6.7.1 Prohibited Medications and Procedures

Study participants should not use the following medications concurrent with use of study products: acyclovir, valacyclovir, and tenofovir disoproxil fumarate/emtricitabine. Should participants report use of any of these medications, they will be required to discontinue use of study products, but will continue to complete all scheduled study visits. Participants who report medication use for post-exposure prophylaxis for possible HIV exposure will be advised to discontinue use of study products and complete all scheduled study visits.

The following devices and preparations should not be used during study participation: diaphragm, vaginal ring, and/or spermicide and all non-study vaginal products other than tampons during menstruation. Participants who report current use of these preparations and devices will be counseled regarding the use of alternative methods and provided or referred to family planning services as needed for provision of alternative methods. Participants are not expected to require gynecologic surgical procedures during follow-up; however, should such a procedure be required, the site IoR or designee will consult the PSRT regarding ongoing product use by the participant

6.7.2 Recommended Medications and Procedures

Study sites will provide a single brand of latex male condoms and a single brand of panty liners to study participants for use during study participation. Instructions and counseling on use of these products will be provided as needed throughout study participation. Male condoms will not be impregnated or coated with spermicide. In the event that a participant needs additional male condoms or panty liners between visits, she may request these from clinic staff at any time.

7 STUDY PROCEDURES

An overview of the study visits and evaluations schedule is presented in Appendix I. Presented in this section is additional information on visit-specific study procedures. A detailed instruction guide will be provided in the MTN-001 SSP Manual.

In addition to any Interim Visits that may occur in accordance with guidance outlined in Section 7.5, the following visits should take place for study participants:

- Screening
- Enrollment (Period 1 Start)
- 3-Week Visit (Mid-Study-Period Visit)
- 6-Week Visit (Period 1 End)
- 7-Week Visit (Period 2 Start)

- 10-Week Visit (Mid-Study-Period Visit)
- 13-Week Visit (Period 2 End)
- 14-Week Visit (Period 3 Start)
- 17-Week Visit (Mid-Study-Period Visit)
- 20-Week Visit (Period 3 End)
- 21-Week Visit (Termination Visit)

Participants will be in an oral dosing period, vaginal dosing period, and dual dosing period, once each (with the order depending on randomization) during the three six-week study periods (Study Period 1, Study Period 2, and Study Period 3). The Enrollment Visit will be considered Day 0 and will occur no more than 30 days following provision of informed consent for screening. The 3-Week, 10-Week, and 17-Week Visit are at the approximate midpoints of Study Periods 1, 2, and 3. These three visits will all follow the format of the Mid-Study-Period Visit. Each of the three study periods is followed by a one-week washout period. These washout periods are between the 6- and 7-Week Visit, the 13- and 14-Week Visit, and the 20- and 21-Week Visit.

7.1 Screening Visit

After providing written informed consent, potential participants may be screened for eligibility over two or more visits if necessary, and eligibility must be confirmed at the enrollment visit. For participants who do not meet the eligibility criteria, screening will be discontinued when ineligibility is determined.

For participants who are found to be presumptively eligible based on the evaluations listed below at these visits, final eligibility will be confirmed at the Enrollment Visit, scheduled to take place within 30 days of the initial Screening Visit. Unless otherwise specified in the protocol or MTN-001 SSP Manual, laboratory testing for the purpose of determining eligibility will occur prior to (and does not include testing during) the Enrollment Visit.

Table 4: Screening Visit

Screening Visit (up to 30 days prior to Enrollment Visit)	
**Component**	**Procedure/Analysis**
**Administrative**	• Obtain written informed consent for screening<br>• Assign Participant ID (PTID)<br>• Collect demographic information<br>• Collect locator information<br>• Assess behavioral eligibility<br>• Provide reimbursement for study visit<br>• Schedule next visit
**Clinical**	• Collect medical/menstrual history<br>• Collect concomitant medications<br>• Perform physical exam (See Appendix III)<br>• Perform pelvic exam (See Appendix III)<br>• Provide counseling<br>o Contraceptive<br>o HIV pre- and post-test<br>o HIV/STI risk reduction and male condom<br>• *Treat for UTI/RTIs/STIs, treat or refer for other findings<br>• Provide male condoms
**Urine**	• Collect urine sample<br>o Qualitative hCG<br>o Dipstick UA (*and culture if positive for leukocyte esterase or nitrates; may omit if culture not standard of care for UTI diagnosis)<br>o SDA for chlamydia and gonorrhea
**Blood**	• Collect blood samples<br>o Complete blood count<br>o Liver function tests<br>o Serum chemistries<br>o Syphilis serology (confirmatory tests as needed)<br>o HIV-1 test (confirmatory tests as needed)<br>o HBsAg
**Pelvic**	• Collect pelvic samples<br>o Vaginal pH<br>o Vaginal fluid for wet mount (BV, Candida, Trichomonas)<br>o *Herpes culture (at sites where standard of care for diagnosis)<br>o **Pap smear

*If indicated (see also MTN-001 SSP Manual) **If no documented normal pap result within 12 months of screening is available.

7.2 Enrollment Visit

Table 5: Enrollment Visit

<table>
<tr><th colspan="2">Enrollment Visit (Study Period 1 Start)</th></tr>
<tr><th>Component</th><th>Procedure/Analysis</th></tr>
<tr><td>Administrative</td><td>• Obtain written informed consent for enrollment (incl. compr. checklist)
• Review/update locator information
• Confirm behavioral eligibility, required for eligibility assessment
• Schedule next study visit
• Provide reimbursement for visit
• Follow procedures for randomization assignment</td></tr>
<tr><td>Clinical</td><td>• Update medical/menstrual history, required for eligibility assessment
• Update concomitant medications, required for eligibility assessment
• Document pre-existing conditions, required for eligibility assessment
• Perform physical exam (See Appendix III), required for eligibility assessment
• Perform pelvic exam (See Appendix III), required for eligibility assessment
• *Treat for UTI/RTIs/STIs, treat or refer for other findings
• Provide counseling
o Contraceptive
o *HIV pre- and post-test
o HIV/STI risk reduction and male condom
o Protocol adherence, product use/adherence
• Provide male condoms and watch device, offer panty liners</td></tr>
<tr><td>Behavioral</td><td>• Administer baseline behavioral assessment</td></tr>
<tr><td>Urine</td><td>• Collect urine sample
o Qualitative hCG, required for eligibility assessment
o *SDA for chlamydia and gonorrhea
o *Dipstick UA (*and culture if positive for leukocyte esterase or nitrates; may omit if culture not standard of care for UTI diagnosis)</td></tr>
<tr><td>Blood</td><td>• Collect blood specimens
o Complete blood count (not for eligibility assessment)
o Liver function tests (not for eligibility assessment)
o Serum chemistries (not for eligibility assessment)
o *Syphilis serology (confirmatory tests as needed)
o *HIV-1 test (conf. tests as needed) (per standard in site SOP)
o Plasma for storage</td></tr>
<tr><td>Pelvic Samples</td><td>• Collect vaginal pH
• Collect vaginal fluid for wet mount (BV, Candida, Trichomonas)
• Collect CVL for vaginal flora proteomics and markers of inflammation
• *Collect herpes culture (at sites where standard of care for diagnosis)</td></tr>
<tr><td>Study Product Supply</td><td>• Appropriate number of doses of study product(s)</td></tr>
</table>

*If indicated (see also MTN-001 SSP Manual)

7.3 Follow-up Visits

Table 6: Mid-Study-Period Visit

**Mid-Study-Period Visit (3-Week Visit, 10-Week Visit, and 17-Week Visit)**	
**Component**	**Procedure/Analysis**
**Administrative**	• Review/update locator information<br>• Schedule next study visit<br>• Provide reimbursement for study visit
**Clinical**	• Collect interval medical/menstrual history<br>• Review/update concomitant medications<br>• Record/update adverse events<br>• Perform physical exam (See Appendix III)<br>• Perform pelvic exam (See Appendix III)<br>• *Treat for UTI/RTIs/STIs, treat or refer for other findings<br>• Reinforce counseling<br>o Contraceptive<br>o HIV/STI risk reduction and male condom<br>o Protocol adherence, product use/adherence<br>• Provide male condoms, offer panty liners<br>• *Provide watch device
**Behavioral**	• Administer behavioral assessment
**Urine**	• Collect urine sample<br>o Qualitative hCG<br>o *Dipstick UA (*and culture if positive for leukocyte esterase or nitrates; may omit if culture not standard of care for UTI diagnosis)<br>o *SDA for chlamydia and gonorrhea
**Blood**	• Collect blood<br>o Liver function tests<br>o Serum chemistries<br>o Tenofovir<br>o *Syphilis serology (confirmatory tests as needed)<br>o *HBsAg
**Pelvic Specimens**	• *Collect vaginal pH<br>• *Collect vaginal fluid for wet mount (BV, Candida, Trichomonas)<br>• *Collect herpes culture (at sites where standard of care for diagnosis)
**Study Product Supply**	• Collect unused study product (gel and/or tablets)<br>• Appropriate number of doses of study product(s)

*If indicated (see also MTN-001 SSP Manual)

Table 7: End of Study Period Visit

**End of Study Period Visit (6-Week Visit, 13-Week Visit and 20-Week Visit)**	
**Component**	**Procedure/Analysis**
**Administrative**	• Review/update locator information<br>• Schedule next study visit<br>• Provide reimbursement for study visit
**Clinical**	• Collect interval medical/menstrual history<br>• Review/update concomitant medications<br>• Record/update adverse events<br>• Perform physical exam (See Appendix III)<br>• Perform pelvic exam (See Appendix III)<br>• *Treat for UTI/RTIs/STIs, treat or refer for other findings<br>• Reinforce counseling<br>o Contraceptive<br>o HIV/STI risk reduction and male condom<br>o Protocol adherence<br>• Male condoms, panty liners
**Behavioral**	• Administer behavioral assessment
**Urine**	• Collect urine sample<br>o Qualitative hCG<br>o *SDA for chlamydia and gonorrhea<br>o *Dipstick UA (*and culture if positive for leukocyte esterase or nitrates; may omit if culture not standard of care for UTI diagnosis)
**Blood**	• Insert lock for specimen collection (may be omitted for non-intensive PK)<br>• Collect blood specimens (see also Section 7.8 for time points)<br>o Pre-dose<br>▪ Liver function tests<br>▪ Serum chemistries<br>▪ Flow cytometry (at sites with capacity)<br>▪ Tenofovir<br>▪ Plasma for storage<br>▪ Peripheral blood mononuclear cells (PBMC) for intracellular tenofovir diphosphate (sites with capacity)<br>o Post-dose<br>▪ Tenofovir<br>▪ PBMC for intracellular tenofovir diphosphate (sites with capacity)<br>o *Syphilis serology (confirmatory tests as needed)<br>o *HBsAg<br>• *Remove lock after specimen collection completed (as indicated)
	(CONTINUED)

**End of Study Period Visit<br>(6-Week Visit, 13-Week Visit and 20-Week Visit) (CONTINUED FROM PREVIOUS PAGE)**	
**Component**	**Procedure/Analysis**
**Pelvic Specimens**	• Collect vaginal pH<br>• Collect vaginal fluid for wet mount (BV, Candida, Trichomonas)<br>• Collect CVL for vaginal flora proteomics, markers of inflammation, and tenofovir level (see also Section 7.8 for time points)<br>• **Intensive PK/Sub-study ONLY** (see also Section 7.8 for time points)<br>o Collect cervical cytology brush for tenofovir<br>o Collect vaginal biopsies for tenofovir<br>• *Collect herpes culture (at sites where standard of care for diagnosis)
**Study Product Supply**	• Collect unused study product (gel and/or tablets)<br>• *Dispense observed dose(s) of study product(s) (if participant does not bring sufficient unused study product to visit for use as observed dose(s), study product(s) will be dispensed to participant during the study visit)

*If indicated (see also MTN-001 SSP Manual), **Intensive PK/Sub-study (US sites) participants only (see also Section 7.8)

Table 8: 7-Week and 14-Week Study Visit

**7-Week and 14-Week Study Visits (Study Period 2 Start, Study Period 3 Start)**	
**Component**	**Procedure/Analysis**
**Administrative**	• Review/update locator information<br>• Schedule next study visit<br>• Provide reimbursement for study visit
**Clinical**	• Collect interval medical/menstrual history<br>• Review/update concomitant medications<br>• Record/update adverse events<br>• Perform physical exam (See Appendix III)<br>• Perform pelvic exam (See Appendix III)<br>• *Treat for UTI/RTI/STIs, treat or refer for other findings<br>• Reinforce counseling<br>o Contraceptive<br>o HIV/STI risk reduction and male condom<br>o Protocol adherence, product use/adherence<br>• Provide male condoms, offer panty liners<br>• Provide HIV pre- and post-test counseling
**Urine**	• Collect urine sample<br>o Qualitative hCG<br>o *Dipstick urinalysis (*and culture if positive for leukocyte esterase or nitrates; may omit if culture not standard of care for UTI diagnosis)<br>o *SDA for chlamydia and gonorrhea
**Blood**	• Collect blood specimens<br>o Complete blood count<br>o Liver function tests<br>o Serum chemistries<br>o HIV-1 test (confirmatory tests as needed)<br>o *Syphilis serology (confirmatory tests as needed)<br>o *HBsAg
**Pelvic**	• *Collect vaginal pH<br>• *Collect vaginal fluid swab for wet mount for BV, Candida, Trichomonas<br>• *Collect herpes culture (at sites where standard of care for diagnosis)
**Study Product Supply**	• *Collect any unused study product (gel and/or tablets)<br>• Dispense appropriate number of doses of study product(s)

*If indicated (see also MTN-001 SSP Manual)

Table 9: 21-Week Visit

21-Week Visit (Study Termination Visit)	
**Component**	**Procedure/Analysis**
**Administrative**	• Review/update locator information<br>• *Schedule next study visit<br>• Provide reimbursement for study visit
**Clinical**	• Collect interval medical/menstrual history<br>• Review/update concomitant medications<br>• Record/update adverse events<br>• *Perform physical exam (See Appendix III)<br>• *Perform pelvic exam (See Appendix III)<br>• *Treat UTI/RTIs/STIs, treat or refer for other findings<br>• Provide HIV pre- and post-test counseling<br>• Reinforce counseling<br>o Contraceptive<br>o HIV/STI risk reduction and male condom<br>• Provide male condoms
**Behavioral**	• Conduct in-depth interview (subset of participants, see Section 7.7)
**Urine**	• Collect urine sample<br>o Qualitative HCG<br>o *Dipstick urinalysis (*and culture if positive for leukocyte esterase or nitrates; may omit if culture not standard of care for UTI diagnosis)<br>o *SDA for chlamydia and gonorrhea
**Blood**	• Collect blood specimens<br>o Complete blood count<br>o Liver function tests<br>o Serum chemistries<br>o HIV-1 test (confirmatory tests as needed)<br>o Plasma for storage<br>o *Syphilis serology (confirmatory tests as needed)<br>o *HBsAg
**Pelvic**	• *Collect vaginal pH<br>• *Collect vaginal fluid swab for wet mount for BV, Candida, Trichomonas<br>• *Collect herpes culture (at sites where standard of care for diagnosis)
**Study Product Supply**	• *Collect any unused study product (gel and/or tablets)

*If indicated (see also MTN-001 SSP Manual)

7.4 Follow up Procedures for Participants who Discontinue Study Product

Participants who discontinue study product will be encouraged to remain in the study if they are willing, for safety evaluations according to the study follow up schedule with the exceptions described below.

7.4.1 Participants Who Seroconvert to HIV

Study staff will capture seroconversions on study CRFs. As discussed in Section 13.9.2, participants may be offered participation in the MTN Seroconverter Study (MTN-015). Protocol-specified procedures will continue except:

- HIV serology
- All PK assessments
- Provision of study gel
- Provision of study tablets
- Product use/adherence counseling
- Counseling for HIV/STI risk reduction. Counseling will be modified to address primary and secondary HIV/STI prevention for infected women.

7.4.2 Participants Who Become Pregnant

All protocol-specified study procedures will continue except:

- Provision of study gel
- Provision of study tablets
- Product use/adherence counseling
- All PK assessments

7.4.3 Participants Who Become Infected with Hepatitis B

All protocol-specified study procedures will continue except:

- Provision of study gel
- Provision of study tablets
- Product use/adherence counseling
- All PK assessments

7.4.4 Participants Who Voluntarily Discontinue Study Gel and/or Tablets

All protocol-specified study procedures will continue except:

- Provision of discontinued study product(s)
- Product use/adherence counseling (if both products are discontinued)
- All PK assessments applicable during the discontinuation period

7.4.5 Participants Who Are Discontinued from Study Gel and/or Tablet Use by the Site Investigator

All protocol-specified study procedures will continue except:

- Provision of discontinued study product(s)
- Product use/adherence counseling (if both products are discontinued)
- All PK assessments applicable during the discontinuation period

7.5 Interim Contacts and Visits

Interim contacts and visits (those between regularly scheduled follow up visits) may be performed at participant request or as deemed necessary by the investigator or designee at any time during the study. Participants will have a urine pregnancy test at each interim visit. All interim contacts and visits will be documented in participants' study records and on applicable case report forms.

Some Interim Visits may occur for administrative reasons. For example the participant may have questions for study staff or require additional study supplies. Other interim contacts and visits may occur in response to AEs experienced by study participants. When interim contacts or visits are completed in response to participant reports of AEs, study staff will assess the reported event clinically and provide or refer the participant to appropriate medical care.

Table 10: Interim Visits and Contacts

**Interim Visits and Contacts**	
**Component**	**Procedure/Analysis**
**Administrative**	• Review/update locator information<br>• *Schedule next study visit
**Clinical**	• Collect interval medical/menstrual history<br>• Review/update concomitant medications<br>• Record/update adverse events<br>• *Perform physical exam (See Appendix III)<br>• *Perform pelvic exam (See Appendix III)<br>• *Treat UTI/RTIs/STIs, treat or refer for other findings<br>• *Reinforce counseling<br>o Contraceptive<br>o HIV risk reduction and male condom<br>o Product use/adherence<br>o Protocol adherence<br>• *Provide HIV pre- and post-test counseling<br>• *Provide male condoms, offer panty liners<br>• *Provide watch device
**Urine**	• Collect urine sample<br>o Qualitative hCG<br>o *Dipstick urinalysis (*and culture if positive for leukocyte esterase or nitrates; may omit if culture not standard of care for UTI diagnosis)<br>o *SDA for chlamydia and gonorrhea
**Blood**	• *Collect blood specimens<br>o *Complete blood count<br>o *Liver function tests<br>o *Serum chemistries<br>o *HIV-1 test (confirmatory testing as needed)<br>o *Tenofovir<br>o *Syphilis serology (confirmatory testing as needed)<br>o *HBsAg
**Pelvic**	• *Collect vaginal pH<br>• *Collect vaginal fluid swab for wet mount for BV, Candida, Trichomonas<br>• *Collect herpes culture (at sites where standard of care for diagnosis)
**Study Product Supply**	• *Collect any unused study product (gel and/or tablets)<br>• *Dispense doses of product(s)

*If indicated (see also MTN-001 SSP Manual)

7.6 Clinical Evaluations and Procedures

See Appendix III for an outline of physical exam and pelvic exam components.

7.7 Behavioral Measures

The primary behavioral study aims will be addressed using a quantitative instrument and in-depth qualitative interviews (conducted with a subset of study participants). Counts of unused study products, which will be returned to the clinics, also will be compared to reports of adherence.

1. The quantitative instrument will be structured around the following topics:

- Sexual activity
- Product adherence (frequency and duration of use)
- Male condom use (frequency and in combination with study products)
- Timing of sexual activity in relationship to product use
- Intra-vaginal practices
- Sharing of study products, including with whom products shared (also selling of products and product theft)
- Experiences using study products, including obstacles to use, side effects and partner involvement

2. We will identify a random sample of eight participants from five sites (Umkomaas CRS, Botha's Hill CRS, Makerere University-JHU Research Collaboration CRS, Case CRS, and Pitt CRS) to complete an in-depth interview that addresses use of study drugs and male condoms during the trial. These interviews will be conducted by a trained study interviewer and will follow a structured questionnaire guide. They will be approximately 30 minutes in duration and will be conducted at the 21-Week study visit. Participants will be compensated for their completion of the in-depth interview. These interviews will be digitally recorded using a handheld digital voice recorder and this file will be transcribed and translated for analysis.

If, at a particular site, women with differential adherence between study products are not included in the random sample of in-depth interview participants, up to two additional participants from that site, who did report differential adherence between products, will be invited to complete an in-depth interview as well. Reported adherence levels will be monitored by site staff so that eligible women can be identified. These interviews will be conducted within one month of the 21-Week study visit.

The interviews will include the following topics:

- Challenges to use of study products
- Perceived benefits of use
- Preferences between oral and vaginal formulations

- Preferences between a single and dual use regimen
- Partner knowledge of study participation and reaction to product use
- Who knew that they had access to anti-retroviral drugs
- Whether they were ever asked to share (or sell) the product or if someone tried to take it away from them

7.8 Pharmacokinetic Procedures

7.8.1 Pharmacokinetic Procedures: All Participants

During all three study periods (single formulation and dual formulation), participants will provide specimens for PK measures. There are two types of visits that include PK measures: the Mid-Study-Period Visits and the End of Study Period Visits. As noted below and except where limited by site capacity, some PK procedures will be applicable for all participants.

Mid-Study-Period Visits

All participants will provide blood samples for tenofovir levels at Mid-Study-Period Visits (3-Week, 10-Week, and 17-Week). Every effort will be made to record the three doses of tenofovir taken prior to these visits with hour: minute accuracy. These visits do NOT include an observed dose of study product(s) in the study clinic.

End of Study Period Visits

Both US and non-US participants will have an observed dose of study product(s) at these visits.

All participants will provide a blood sample prior to their observed dose of study product(s) at the End of Study Period Visits (6-Week, 13-Week, and 20-Week). This blood sample will be used for:

- Blood tenofovir level
- *At sites with capacity,* cell lysate (intracellular tenofovir diphosphate)
- *At sites with capacity*, cell counts by flow cytometry. These cell counts serve as surrogate markers for cell activation and proliferation, respectively, and will be used as covariates during intracellular model building.

7.8.2 Pharmacokinetic Procedures: Non-Intensive PK Participants

At non-US sites, participants will take part at End of Study Period Visits (6-Week, 13-Week, and 20-Week) in the collection of samples for a Non-Intensive PK portion of the study.

Table 11: Non-Intensive PK Participants Only

	PRE-DOSE	POST-DOSE TIMING		
		1-3 HOURS	3-5 HOURS	5-7 HOURS
**Blood:**<br>• Flow cytometry (at sites with capacity)	**Study Regimen Sequences A, B, C, D, E, and F**			
**Blood:**<br>• PBMC cell lysate (intracellular tenofovir diphosphate) (at sites with capacity)<br>• Tenofovir	**Study Regimen Sequences A, B, C, D, E, and F**	**Study Regimen Sequences E and F**	**Study Regimen Sequences A and B**	**Study Regimen Sequences C and D**
**CVL**<br>• Tenofovir<br>• Proteomics and markers of inflammation		**Study Regimen Sequences E and F**	**Study Regimen Sequences A and B**	**Study Regimen Sequences C and D**

For simplicity, Non-Intensive PK Participants will be assigned to a sampling window based on their sequence randomization assignment:

- 1-3 hours, Sequences E and F
- 3-5 hours, Sequences A and B
- 5-7 hours, Sequences C and D

Thus, it is expected that Non-Intensive PK Participants will have one sampling time pre-dose (blood), and only one sampling time post-dose (blood and CVL).

For individual participants, post-dose blood and CVL samples will occur within 15 – 30 minutes of each other (either sample may be taken first). The times of these samples must be recorded with hour: minute accuracy. The same sampling time point (within 15 minutes) in each study period should be used for all of a participant's End of Study Period Visits.

The time of the observed dose(s) of study product(s) administered in clinic and the three prior doses taken must be recorded with hour: minute accuracy. Participants will

receive a small watch or similar timekeeping device to assist them in remembering or recording (on a study-provided form) the times of these doses. An additional device may be provided in the event of a lost or stolen device.

7.8.3 Pharmacokinetic Procedures: Intensive PK Participants (US Sites)

All US sites will participate in Intensive PK measures (n=72). These Intensive PK measures will occur at the End of Study Period Visits during all three study periods. Participants in the Intensive PK portion of the study will provide cervical cells (collected by cytology brush) and vaginal tissue (collected by biopsy at two locations in the vagina) which will be used for measurement of tenofovir levels.

The time of the dose administered in clinic and the three prior doses taken must be recorded with hour: minute accuracy. Participants will receive a small watch or similar timekeeping device to assist them in remembering or recording the times of these doses. Participants taking part in the Intensive PK measures will not follow procedures outlined in Table 10.

For the Intensive PK cohorts at US sites, the End of Study Period sample timing will require a second randomization which will be stratified within each of the two sites. All 72 participants will be randomized into groups (shown below as M, N, O, and P) to provide collection of pelvic exam specimens, either pre-dose, 2, 4, or 6 hours after dosing, providing up to 18 women per time point.

- All Intensive PK participants (ppts) will have blood collected within 15 – 30 minutes of the scheduled dose (pre-dose), and at 1, 2, 4, 6, and 8 hours following dosing. Blood from these specimens will be used for flow cytometry (at pre-dose only), tenofovir levels, and cell lysate (intracellular tenofovir disphosphate).

- Blood, cervical cells (cytology brush), CVL fluid, and vaginal biopsies will be collected according to the schedule outlined in the table below.

- At these three visits, participants will take their assigned dose of oral and/or vaginal tenofovir at the clinic and will undergo collection of their blood, cervical cells, CVL fluids, and vaginal biopsy within 15 – 30 minutes of the assigned sampling time, either pre-dose **or** 2, 4, or 6 hours post-dose.

- The specific post-dosing time point for each participant will be determined as part of the participant’s random assignment.

Table 12: Intensive PK Participants Only (US site participants only)

SPECIMEN	PRE-DOSE	POST-DOSE TIMING				
		1 HOUR	2 HOURS	4 HOURS	6 HOURS	8 HOURS
**Blood draw**<br>• **PBMC cell lysate (intracellular tenofovir diphosphate) (at sites with capacity)**<br>• **Tenofovir**	Groups M, N, O, and P)	Groups M, N, O, and P)	Groups M, N, O, and P)	Groups M, N, O, and P)	Groups M, N, O, and P)	Groups M, N, O, and P)
**Blood draw**<br>• **Flow cytometry (at sites with capacity)**	Groups M, N, O, and P)					
**Cervical cytology brush**<br>• **Cell lysates (intracellular tenofovir diphosphate)**<br>• **Tenofovir**	12 ppts (Group M)		12 ppts (Group N)	12 ppts (Group O)	12 ppts (Group P)	
**CVL**<br>• **Tenofovir**<br>• **Proteomics and markers of inflammation**	Group M		Group N	Group O	Group P	
**Vaginal biopsies**<br>• **Cell lysates (intracellular tenofovir diphosphate)**<br>• **Tenofovir**	Group M		Group N	Group O	Group P	

The blood sample should be taken within 15 – 30 minutes of the vaginal and cervical samples and times recorded for all samples (blood, CVL, cervical cytology brush, and vaginal biopsies). For an individual participant, the same time point (within 15 minutes) in each study period should be used for the oral, vaginal, and dual formulation periods.

Blood, cervical cytology brush, CVL and vaginal biopsy samples will be analyzed for tenofovir concentration. Cells will be extracted from tissue biopsy samples for determination of intracellular tenofovir diphosphate levels. Cell lysates will be analyzed for intracellular tenofovir diphosphate levels. Blood and intracellular samples will be analyzed for routine PK parameters - C_{max}, T_{max}, AUC, and C_{min}.

7.9 Laboratory Evaluations

7.9.1 Local Laboratory Testing

Blood
- Complete blood count
- Serum chemistries
  - o Phosphate
  - o Creatinine (creatinine clearance calculated for every creatinine result)
- Liver function tests
  - o AST
  - o ALT
- HIV serology (rapid test or enzyme-linked immunoassay, WB if indicated; see Appendix II)
- Syphilis serology (confirmatory testing as indicated)
- HBsAg
- Flow cytometry (at sites with capacity)
- PBMC cell lysate isolation (at sites with capacity)

Urine
- Qualitative hCG
- Urinalysis
- Urine culture (if clinically indicated, at sites where this is standard of care)
- SDA for chlamydia and gonorrhea (may ship to a regional Network Laboratory as needed)

Pelvic Specimens
- Pap smear
- Vaginal pH
- Vaginal swabs for wet preparation slide
- Herpes culture (at sites where standard of care for diagnosis, and where capacity exists)

7.9.2 Herold Laboratory Testing

Cervicovaginal Lavage Fluid
- Vaginal flora proteomics
- Markers of inflammation

7.9.3 Network Laboratory Testing

Blood
- HIV-1 confirmatory testing as needed (see Appendix II)
- Tenofovir level

- Cell lysate (intracellular tenofovir diphosphate)
- Plasma archive

Urine
- SDA for chlamydia and gonorrhea (for US sites not currently able to perform this test on site)

Cervicovaginal Lavage Fluid
- Tenofovir level

Cervical Cytology Brush
- Intracellular tenofovir level for Intensive PK participants

Vaginal Tissue Biopsy
- Intracellular tenofovir level for Intensive PK participants

At selected sites, 3 x 5 mm (approximate size) vaginal tissue biopsies will be obtained from a subset of participants for the purpose of measuring tenofovir level in tissue, pending the development of a validated assay.

7.10 Specimen Collection and Processing

Each study site will adhere to the standards of good clinical laboratory practice, the HPTN-MTN Network Laboratory Manual (www.mtnstopshiv.org), DAIDS Laboratory Requirements (http://www3.niaid.nih.gov/research/resources/ DAIDSClinRsrch/Labs/), MTN-001 SSP Manual (www.mtnstopshiv.org), and site standard operating procedures for proper collection, processing, labeling, transport, and storage of specimens at the local laboratory. Specimen collection, testing, and storage at the site laboratories will be documented when applicable using the Laboratory Data Management System (LDMS). In cases where laboratory results are not available due to administrative or laboratory error, sites are permitted to re-draw specimens that are intended for use in the screening process.

7.11 Specimen Handling

Specimens will be handled in accordance with Requirements for DAIDS Sponsored and/or Funded Laboratories in Clinical Trials (http://www3.niaid.nih.gov/research/resources/DAIDSClinRsrch/Labs/).

7.12 Biohazard Containment

As the transmission of HIV and other blood-borne pathogens can occur through contact with contaminated needles, blood, and blood products, appropriate blood and secretion precautions will be employed by all personnel in the drawing of blood and shipping and handling of all specimens for this study as recommended by the CDC and NIH. All biological specimens will be transported using packaging mandated by CFR 42 Part 72.

All dangerous goods materials, including diagnostic specimens and infectious substances, must be transported according to instructions detailed in the International Air Transport Association (IATA) Dangerous Goods Regulations. This applies to both US and international sites. Biohazardous waste will be contained according to institutional, transportation/carrier, and all other applicable regulations.

7.13 Final Contact

The 21-Week Visit for all participants will include laboratory testing. In addition, some participants will have an in-depth interview as described in Section 7.7. As results are not expected to be available on the same day for participants, a final contact (in person or by telephone (except for HIV test results)) may be required to provide the final study test results, post-test counseling, and treatment from these visits. In addition, for participants who become pregnant prior to the study end date, an additional contact may be required to ascertain the participant's pregnancy outcome. Study sites may complete the final contact visit(s) at the study site or at community based locations, depending on site capacities and site and participant preferences. All final contacts must be documented in participant study records.

8 ASSESSMENT OF SAFETY

8.1 Safety Monitoring

The study site Investigators are responsible for continuous close safety monitoring of all study participants, and for alerting the Protocol Team if unexpected concerns arise. A sub-group of the Protocol Team, including the Protocol Chair, DAIDS Medical Officer, MTN CORE Protocol Safety Physicians, SDMC Clinical Affairs Research Nurse, and Protocol Statistician, will serve as the PSRT; the PSRT will be chaired by the MTN CORE Protocol Safety Physicians. Not all of these members are required for quorum, which will be outlined further in the MTN-001 SSP Manual. The MTN SDMC will prepare routine safety data reports for review by the PSRT, which will meet via conference call approximately once per month or as needed throughout the period of study implementation to review safety data, discuss product use management and address any potential safety concerns. The content, format and frequency of safety data reports will be agreed upon by the PSRT and the SDMC in advance of study implementation.

8.2 Clinical Data Safety Review

A multi-tiered safety review process will be followed for the duration of this study. The study site investigators are responsible for the initial evaluation and reporting of safety information at the participant level, and for alerting the PSRT if unexpected concerns arise. Participant safety is also monitored at the Network level through a series of routine reviews conducted by the SDMC Clinical Affairs staff, the PSRT and study

sponsors. Additional reviews may be conducted at each of these levels as dictated by the occurrence of certain events.

MTN SDMC Clinical Affairs staff will review incoming safety data on an ongoing basis. Events identified as questionable, inconsistent, or unexplained will be queried for verification. Adverse event reports submitted in an expedited manner to the DAIDS Safety Office will be forwarded to the DAIDS Medical Officer and SDMC Clinical Affairs staff for review.

The PSRT will meet regularly via conference call to review clinical data reports generated by the MTN SDMC. The content, format and frequency of the clinical data reports will be agreed upon by the PSRT and the SDMC in advance of study implementation. In addition to the routine safety data reviews, the PSRT will convene on an ad hoc basis to make decisions regarding the handling of any significant safety concerns. If necessary experts external to the MTN representing expertise in the fields of microbicides, biostatistics, HIV transmission and medical ethics may be invited to join the PSRT safety review. A recommendation to stop the trial may be made by the PSRT at this time or at any such time that the team agrees that an unacceptable type and/or frequency of AEs has been observed.

Recommendations regarding permanent discontinuation of one or both study products in the study as a whole may involve sponsor consultation with the US Food and Drug Administration (FDA).

In the unlikely event that the protocol team or PSRT has serious safety concerns that lead to a decision to permanently discontinue one or both study products for all participants and stop accrual into the study, the protocol team or PRST will request a review of the data by the Study Monitoring Committee (SMC) before recommending that the study be stopped. Members of the SMC will be independent investigators with no financial interest in the outcomes of this study. If at any time, a decision is made to discontinue one or more study products in all participants, DAIDS will notify the US FDA and the site investigators of record will notify the responsible IRBs/ECs expeditiously.

8.3 Adverse Events Definitions and Reporting Requirements

8.3.1 Adverse Events

An AE is defined as any untoward medical occurrence in a clinical research participant enrolled in a clinical trial and which does not necessarily have a causal relationship with an investigational product or study participation. As such, an AE can be an unfavorable or unintended sign (including an abnormal laboratory finding, for example), symptom or disease temporally associated with the use of an investigational product or study participation, whether or not considered related to the product or study participation. This definition will be applied beginning from the time of random assignment. The term "investigational product" for this study refers to the TDF 300 mg tablets, tenofovir gel, and study gel applicator.

Study participants will be instructed to contact the study site staff to report any AEs they may experience. In the case of a life-threatening event, they will be instructed to seek immediate emergency care. Where feasible and medically appropriate, participants will be encouraged to seek medical care where the study clinician is based, and to request that the clinician be contacted upon their arrival. With appropriate permission of the participant, whenever possible records from all non-study medical providers related to AEs will be obtained and required data elements will be recorded on study case report forms. All participants reporting an AE (including pelvic exam abnormalities) will be followed clinically until the AE resolves (returns to baseline) or stabilizes.

The site IoR will determine AE resolution or stabilization in their best clinical judgment, but may seek PSRT consultation regarding follow up or additional evaluations of an AE. The PSRT will review the query and provide a consensus response regarding follow up and/or additional evaluation to the site IoR.

Study site staff will report on study case report forms all AEs reported by or observed in enrolled study participants from the time of enrollment (random assignment) until study termination, regardless of severity and presumed relationship to study product. The Female Genital Grading Table for Use in Microbicide Studies (included in Appendix IV) will be the primary tool for grading adverse events for this protocol, with the exception of asymptomatic bacterial vaginosis which will not be a reportable AE. Adverse events not included in that table will be graded by the DAIDS AE Grading Table Version 1.0, December 2004. In cases where an AE is covered in both tables, the Female Genital Grading Table for Use in Microbicide Studies will be the grading scale utilized.

Participants will be encouraged to report to the study clinician any problems experienced by their male partners that might be potentially related to study product. If any such problems are reported, study staff should evaluate and document the occurrence and the Investigator of Record (or designee) should inform the PSRT, so that this information can be considered during routine PSRT safety data reviews. Should any concerns arise with regard to partner safety the PSRT will advise all study sites on appropriate action.

8.3.2 Serious Adverse Event

Serious adverse events (SAEs) will be defined per CFR 312.32 guidelines, as AEs occurring at any dose that:

- Result in death
- Are life-threatening adverse events
- Require Inpatient hospitalization or prolongation of existing hospitalization

- Result in persistent or significant disability/incapacity, or
- Are congenital anomalies/birth defects.

Important medical events that may not result in death, be life-threatening, or require hospitalization may be considered serious when, based upon appropriate medical judgment, they may jeopardize the participant or require medical or surgical intervention to prevent one of the outcomes listed above.

8.3.3 Adverse Event Relationship to Study Product

The relationship of all AEs to study product will be assessed per the Manual for Expedited Reporting of Adverse Events to DAIDS (dated 6 May 2004), the tenofovir gel investigator's brochure, the Viread® package insert, and clinical judgment. Per the Manual for Expedited Reporting of Adverse Events to DAIDS, the relationship categories that will be used for this study are:

- *Definitely related*: adverse event and administration of study agent are related in time, and a direct association can be demonstrated with the study agent.
- *Probably related*: adverse event and administration of study agent are reasonably related in time, and the adverse event is more likely explained by the study agent than by other causes.
- *Possibly related*: adverse event and administration of study agent are reasonably related in time, and the adverse event can be explained equally well by causes other than the study agent.
- *Probably not related*: a potential relationship between administration of study agent and adverse event could exist, but is unlikely, and the adverse event is most likely explained by causes other than the study agent.
- *Not related*: the adverse event is clearly explained by another cause unrelated to administration of the study agent. Reportable events must have documentation to support the determination of "not related".

8.4 Expedited Adverse Event Reporting Requirements

Expedited Adverse Event Reporting to DAIDS
The expedited adverse event (EAE) reporting requirements and definitions for this study and the methods for expedited reporting of adverse events (AEs) to the DAIDS Regulatory Compliance Center (RCC) Safety Office are defined in "The Manual for Expedited Reporting of Adverse Events to DAIDS" (DAIDS EAE Manual), **dated May 6, 2004**. The DAIDS EAE Manual is available on the RCC website: http://rcc.tech-res-intl.com/.

AEs reported on an expedited basis must be documented on the DAIDS Expedited Adverse Event Reporting Form (EAE Reporting Form) available on the RCC website: http://rcc.tech-res-intl.com.

DAIDS EAE forms should be submitted to DAIDS through the Regulatory Compliance Center (RCC) Safety Office (rccsafetyoffice@tech-res.com) or call 1-800-537-9979 or 301-897-1709 or fax 1-800-275-7619 or 301-897-1710.

EAE Reporting Requirements for this Study

EAE Reporting Level

This study uses the Standard Level of expedited AE reporting as defined in the DAIDS EAE Manual.

Study Agents for Expedited Reporting to DAIDS

The study agents that must be considered in determining relationships of AEs requiring expedited reporting to DAIDS are: TDF 300 mg tablet, tenofovir 1% gel, and study gel applicator.

Grading Severity of Events

The Female Genital Grading Table for Use in Microbicide Studies (included in Appendix IV) will be the primary tool for grading adverse events for this protocol, with the exception of asymptomatic bacterial vaginosis which will not be a reportable AE. Adverse events not included in that table will be graded by the DAIDS AE Grading Table Version 1.0, December 2004. In cases where an AE is covered in both tables, the Female Genital Grading Table for Use in Microbicide Studies will be the grading scale utilized.

The Division of AIDS Table for Grading the Severity of Adult and Pediatric Adverse Events (DAIDS AE Grading Table), Version 1.0, December, 2004, is available on the RCC website at http://rcc.tech-res-intl.com/.

EAE Reporting Periods

AEs must be reported on an expedited basis at the Standard Level during the Protocol-defined EAE Reporting Period, which is the entire study duration for an individual participant (from study enrollment until study termination).

For each study participant, expedited AE reporting will be undertaken throughout the scheduled duration of follow-up, i.e., from the time of random assignment through completion of the 21-Week Visit. After the 21-Week Visit, pregnancy outcomes that meet criteria for expedited AE reporting (e.g., fetal losses, congenital anomalies) occurring among participants known to be pregnant at Week 21 will be reported. In addition, should site staff become aware of any serious, unexpected, clinical suspected adverse drug reactions after Week 21, such events also will be reported as EAEs.

8.5 Local Regulatory Requirements

Site investigators will submit AE information in accordance with local regulatory agencies' or other local authorities' requirements. This reporting will include site IRB/EC-mandated reporting of AEs, SAEs, and other relevant safety information.

8.6 Social Harms Reporting

Although study sites make every effort to protect participant privacy and confidentiality, it is possible that participants' involvement in the study could become known to others, and that social harms may result (i.e., because participants could become known as HIV-infected or at "high risk" for HIV infection). For example, participants could be treated unfairly or discriminated against, or could have problems being accepted by their families and/or communities. Social harms that are judged by the Investigator of Record to be serious or unexpected will be reported to responsible site Institutional Review Boards/Ethics Committees (IRBs/ECs) at least annually, or according to their individual requirements. In the event that a participant reports social harm, every effort will be made by study staff to provide appropriate care and counseling to the participant, and/or referral to appropriate resources for the safety of the participant as needed. While maintaining participant confidentiality, study sites may engage their Community Advisory Boards in exploring the social context surrounding instances of social harm.

9 CLINICAL MANAGEMENT

Guidelines for clinical management and product hold/discontinuation are outlined in this section.

In general, the site investigator has the discretion to hold study product at any time if s/he feels that continued product use would be harmful to the participant, or interfere with treatment deemed clinically necessary according to the judgment of the investigator. Unless otherwise specified below, the investigator should immediately consult PSRT for further guidance in restarting study drug(s) or progressing to permanent discontinuation.

9.1 Grading System

The grading system is located in the Female Genital Grading Table for Use in Microbicide Studies, which can be found in Appendix IV, and in the Division of AIDS Table for Grading Severity of Adult and Pediatric Adverse Events (DAIDS AE Grading Table), Version 1.0, December 2004, which can be found on the Regulatory Compliance Center (RCC) Web site: http://rcc.tech-res-intl.com/eae/htm/.

9.2 Dose Modification Instructions

No dose reductions are allowed.

9.3 Discontinuation of Study Product(s) in the Presence of Toxicity

Grade 1 or 2

In general, participants who develop a Grade 1 or 2 AE regardless of relatedness to study product that is not specifically addressed below may continue use of both study products per protocol.

Grade 3

Participants who develop a Grade 3 adverse event or toxicity that is not specifically addressed below and is judged to be possibly, probably, or definitely related to study product should have that study product held. In general, and unless otherwise decided in consultation with the PSRT, the investigator should re-evaluate the participant at least weekly up to 2 weeks. If documentation is not available within 2 weeks to show that the adverse event is $\leq$ Grade 2, the current study product must be permanently discontinued.

If the same Grade 3 adverse event recurs after reintroduction of study product, the current study product must be permanently discontinued if the investigator considers the adverse event probably not, possibly, probably, or definitely related to study product. However if the investigator determines that the toxicity is definitely not related to study product, participants may continue the study product and the PSRT must be notified.

Grade 4

Participants who develop a Grade 4 adverse event or toxicity that is not specifically addressed below (regardless of relationship to study product(s)) should have the current study product(s) held. If the investigator determines that the toxicity is definitely not related to study product(s), PSRT may be consulted to consider restarting study product(s), but product(s) should be held until a recommendation from PSRT is obtained. The participant should be re-evaluated at least weekly up to 2 weeks. If documentation is not available within 2 weeks to show that the adverse event is $\leq$ Grade 2, the study product(s) must be permanently discontinued. If the same Grade 4 adverse event recurs at either Grade 3 or 4 level after reintroduction of study product(s), study product(s) must be permanently discontinued.

9.4 General Criteria for Discontinuation of Study Product

Participants may voluntarily discontinue one or both study products for any reason at any time. Site IoRs will temporarily hold or permanently discontinue participants from one or both study products per protocol for any of the specific criteria below, which may be further clarified in the SSP Manual. Site IoRs also may temporarily hold or permanently discontinue participants from one or both study products for use of prohibited medication (per Section 6.7.1), for reasons not shown here or in the SSP Manual, e.g., to protect participant safety and/or if participants are unable or unwilling to comply with study product use procedures. In such cases, the Site IoRs would temporarily hold product use and provide a written query with a request for permanent

study product discontinuation to the PSRT for review. The PSRT will provide a written response to the site indicating whether the PSRT has recommended permanent discontinuation of study product(s). Such recommendations regarding permanent discontinuation of one or both study products in individual participants will be made by the PSRT based on careful review of all relevant data.

The criteria for permanent discontinuation of further study product use of one or both study products for an individual participant are:

- Study product-related toxicity requiring permanent discontinuation of study product(s) per Section 9 of this protocol
- Completion of regimen as defined in the protocol
- Request by participant to terminate study product(s)
- Clinical reasons determined by the physician
- HIV infection
- Hepatitis B infection
- Pregnancy or breastfeeding

If a participant is permanently discontinued from oral study product, and if the Tenofovir 1% Gel Period has not yet begun for this participant, the investigator in consultation with the PSRT may decide that she may participate per protocol in the Tenofovir 1% Gel Period as scheduled according to her sequence randomization. If a participant is permanently discontinued from gel study product, and if the TDF 300 mg Oral Tablet Period has not yet begun for this participant, the investigator in consultation with the PSRT may decide that she may participate per protocol in the TDF 300 mg Oral Tablet Period as scheduled according to her sequence randomization.

However, a participant who has been permanently discontinued from either type of study product will not take any study product during the Dual Formulation Period (if this study period has not yet begun for an individual participant), but will be followed according to regularly scheduled evaluations of safety, according to Section 7.4.5.

Participants may initiate study product use per protocol in a subsequent study period (if applicable), provided other product hold guidelines do not apply. Participants must meet applicable criteria for restarting a particular study product before initiating that study product's use in a subsequent study period.

9.5 Management of Specific Toxicities

Specific guidance related to product hold is also noted here as it pertains to the clinical management of toxicities.

9.5.1 Nausea and Vomiting

Participants with Grade 1 and/or 2 nausea or vomiting may be treated symptomatically with hydration, oral antiemetic therapies or antiemetic suppositories. Participants should be instructed to take oral study product with food.

ORAL STUDY PRODUCT

Participants with Grade ≥ 3 nausea and/or vomiting must hold the study product until the toxicity grade returns to Grade ≤ 2 and be treated symptomatically. Therapy should be resumed using the full doses of study product. If Grade ≥ 3 nausea and/or vomiting recurs upon the resumption of study product despite symptomatic treatment, study product must be permanently discontinued.

VAGINAL STUDY PRODUCT

Unless other product hold guidelines apply, vaginal study product may be continued at the discretion of the investigator.

9.5.2 Diarrhea

Participants with diarrhea of any toxicity grade may be treated symptomatically with permitted antimotility agents and rehydration at the discretion of the site investigator.

ORAL STUDY PRODUCT

Participants with new onset Grade ≥ 3 diarrhea that is unresponsive to antimotility agents and for which an alternative etiology (e.g., infectious diarrhea) is not established must hold the oral study product until the toxicity grade returns to Grade ≤ 2 or to baseline and be treated symptomatically. Oral study product administration should be resumed using the full dose of oral study product. If Grade ≥ 3 diarrhea recurs upon the resumption of oral study product despite symptomatic treatment, oral study product must be permanently discontinued.

VAGINAL STUDY PRODUCT

Unless other product hold guidelines apply, vaginal study product may be continued at the discretion of the investigator.

9.5.3 AST/ALT Elevations

Careful assessments should be done to rule out the use of alcohol, non-study medication-related drug toxicity, or viral hepatitis as the cause of elevation in AST or ALT of any grade.

ORAL STUDY PRODUCT

The participant must be carefully assessed for any symptoms or signs of hepatotoxicity, including fatigue, malaise, anorexia and nausea, jaundice, acholic stools, RUQ pain or hepatomegaly. If the AST/ALT elevation is considered most likely to be due to concomitant illness or medication, standard management, including discontinuation of the likely causative agent, should be undertaken.

If symptoms or signs of clinical hepatitis are present, study treatment must be discontinued (see below). Careful assessments should be undertaken for alcohol use, non-study medication-related drug toxicity, the lactic acidosis syndrome, and viral hepatitis as the cause of the transaminase elevation. If the AST/ALT elevation is considered most likely to be due to concomitant illness or medication, standard management, including discontinuation of the likely causative agent, should be undertaken.

Grade 1

For study participants with Grade 0 ALT and AST at study entry, an increase to Grade 1 ALT or AST even in an asymptomatic participant may be of concern.

ALT and AST must be repeated as soon as possible (at most within 1 week) of a new Grade 1 ALT or AST. Study treatment may be continued while repeating ALT and AST at the discretion of the investigator provided the participant is asymptomatic.

Participants with a confirmed Grade 1 ALT or AST who are asymptomatic may continue study medications with continued close observation.

Grade 2

Participants should have ALT/AST re-checked as soon as possible (at most within 1 week) and then be followed weekly until levels are Grade ≤ 1. The frequency of follow up may be altered at the discretion of the site investigator following consultation with the PSRT. Study treatment may continue at the discretion of the investigator provided the participant is asymptomatic.

Grade 3

Study product should be held for any ALT or AST of Grade 3.

Participants should have ALT/AST re-checked as soon as possible (at most within 1 week). Participants should then be followed weekly until levels are Grade ≤ 1, at which point study medication may be restarted with close follow-up and in consultation with

the PSRT. If lab documentation is not available that levels have returned to Grade ≤ 1 within three weeks, study product must be permanently discontinued.

For participants who have restarted study product, any level of Grade 3 or above should result in permanent discontinuation of study product.

Grade 4
Study product should be permanently discontinued. ALT/AST must be followed at least weekly until Grade ≤1.

VAGINAL STUDY PRODUCT
Unless other product hold guidelines apply, vaginal study product may be continued at the discretion of the investigator.

9.5.4 Creatinine Clearance

If the creatinine clearance is <50mL/min, it should be confirmed within 1 week of the receipt of the results in consultation with the PSRT.

ORAL STUDY PRODUCT
If the creatinine clearance is confirmed to be <50mL/min, the oral study product must be permanently discontinued. If the participant fails to have the creatinine clearance confirmed in one week, all attempts should be made by the site to contact the participant to have the creatinine clearance confirmed within three working days. Participants who fail to have a confirmed test will permanently discontinue the oral study product.

VAGINAL STUDY PRODUCT
Unless other product hold guidelines apply, vaginal study product may be continued at the discretion of the investigator.

9.5.5 Hypophosphatemia

For Grades 1 and 2 hypophosphatemia, the phosphate should be repeated within 2 weeks of the receipt of the results. Supplemental phosphate should be given with phosphate-rich food or fluid with or without neutral phosphate solution. For Grades 3 and 4 hypophosphatemia, the phosphate should be repeated within 1 week of the receipt of the results. Supplemental phosphate should be given with phosphate-rich food or fluid with or without neutral phosphate solution, and other causes of low phosphate should be investigated. During the time that supplemental phosphate is provided to the participant and the time that testing is repeated, sites should follow product hold guidelines described in Section 9.3.

ORAL STUDY PRODUCT

If documentation is not available within 1 week to show response to supplementation (resolution to Grade $\leq$ 2), the oral study product must be permanently discontinued.

VAGINAL STUDY PRODUCT

Unless other product hold guidelines apply, vaginal study product may be continued at the discretion of the investigator.

9.5.6 Genital Sexually Transmitted Infection/Reproductive Tract Infection

Management of sexually transmitted infections commonly referred to as STIs and other forms of vaginitis and cervicitis will be in accordance with current WHO guidelines, available at http://www.who.int/en/.

ORAL STUDY PRODUCT:

Oral study product need not be held in the event of genital STI/RTI requiring treatment, unless other product hold guidelines apply.

VAGINAL STUDY PRODUCT

For Grade 2 genital STI(s)/RTI(s) requiring treatment, vaginal study product may be held until treatment is completed and symptoms have resolved at the judgment of the investigator. Once treatment is complete and any symptoms have resolved, vaginal study product use can be resumed unless other product hold guidelines apply.

9.6 HIV and Hepatitis B

Participants who are identified as infected with HIV and/or hepatitis B will be managed or referred for management according to the local standard of care. For additional guidance regarding participants who are identified as infected with HIV, please see Section 13.9.2.

NOTE: Participants who are identified as infected with HIV and/or hepatitis B will discontinue permanently all study product(s).

9.7 Clinical Management of Pregnancy

All study participants are required to be using an effective method of contraception according to Section 5.2 at enrollment, and intending to use same method for the duration of study participation. Study staff will provide contraceptive counseling to enrolled participants as needed throughout the duration of study participation and will facilitate access to contraceptive services through direct service delivery and/or active referrals to local service providers. Study staff also will provide participants with male condoms and counseling on use of condoms ideally during every sex act during study participation.

Pregnancy testing will be performed at all study visits and participants will be encouraged to report all signs or symptoms of pregnancy to study staff. The site IoR or designee will counsel any participants who become pregnant regarding possible risks to the fetus according to site-specific SOPs. The IoR or designee also will refer the participant to all applicable services; however, sites will not be responsible for paying for pregnancy-related care.

Participants who are pregnant at the termination visit will continue to be followed until the pregnancy outcome is ascertained (or, in consultation with the PSRT, it is determined that the pregnancy outcome cannot be ascertained). Pregnancy outcomes will be reported on relevant case report forms; outcomes meeting criteria for EAE reporting also will be reported on EAE forms.

NOTE: Participants who become pregnant during the course of the study will discontinue permanently all study product(s).

9.8 Criteria for Early Termination of Study Participation

Participants may voluntarily withdraw from the study for any reason at any time. Site IoRs may, with the approval of the PSRT, withdraw participants before their scheduled termination visit to protect their safety, and/or if participants are unable or unwilling to comply with study procedures. Participants also may be withdrawn if the study sponsors, government or regulatory authorities (including the Office of Human Research Protections (OHRP)), or site IRBs/ECs terminate the study prior to its planned end date. Site investigators are required to consult the Protocol Chair and Protocol Biostatistician prior to the termination of any study participant. Study staff will record the reason(s) for all withdrawals in participants' study records. In the event that participants who voluntarily withdraw from the study wish to re-join the study during their planned 21-week follow-up period, they may resume study procedures and follow-up at the investigator's discretion.

10 STATISTICAL CONSIDERATIONS

10.1 Overview and General Design

This is a three-period, three treatment, multi-site, open label, crossover study. All the enrolled women will use all three treatments (i.e. oral TDF, tenofovir 1% gel, and a period of using both products), each in a separate period. The sequence of the three treatments will be randomly assigned. The total length of follow-up is 21 weeks: three periods of 6 weeks on treatment, each followed by a one-week washout period.

Each one-week washout period will be used to collect adverse events that may have resulted from product use during the previous period.

10.2 Study Endpoints

10.2.1 Study Primary Endpoints

Consistent with the primary study objectives, the following primary endpoints will be assessed:

- **Adherence.** Participant self reported product use. For each woman, adherence to each regimen will be computed by dividing the number of daily doses she reports having taken (numerator) by the number of expected doses if she were fully adherent (denominator).

- **Acceptability.** The proportion of participants who indicate that they would be "unlikely" to use the study product in the future.

- **PK.** AUC, C_{max}, and C_{min} associated with oral, vaginal, and dual use regimens.

10.2.2 Study Secondary Endpoints

Consistent with the secondary study objectives, the following secondary endpoints will be assessed:

- Proportion of women who report taking at least 90% of expected daily doses, frequency of use during the follow-up interval using an ordinal measure (5 categories of use, never to always); number of days product missed or not used during the previous week

- Frequency (ordinal measures) of sexual activity and male condom use

- Time interval between product usage and sexual intercourse; sequence of product use and sexual intercourse

- Reported sharing of study product; quantity of shared study product

- Grade 3 or higher toxicity for systemic and local effects as defined by DAIDS AE Grading Table Version 1.0, December 2004, or Grade 3 or higher genital infection, pain or epithelial lesion as defined by the Female Genital Grading Table for Use in Microbicide Studies which cannot be directly attributed to another cause, and judged as definitely, probably, possibly, or probably not related to the study gel, applicator, or study tablet

10.3 Study Hypotheses

The study hypotheses for the primary objective are:

- There will be no differences in rates of adherence among the three study regimens

- There will be no difference in rates of acceptability among the three study regimens

- Tissue levels of PMPA will be similar irrespective of the route of administration

- Oral TDF will be associated with higher concentrations of PMPA in the blood compared to topical PMPA

- Neither tenofovir 1% gel nor oral TDF regimens will adversely impact the genital tract environment

10.4 Sample Size

Sample size/power formulas for a parallel design with two arms (i.e. two different groups of women) can be used to compute sample size/power. Then, this sample size resulting from the assumption of independent groups can be adjusted to reflect that there will be intra-woman correlation in the crossover design. The sample size adjustment can be obtained by this formula:

N' = N (1- rho)/2

where N' is the sample size for a crossover study, N is the total number of women required in a parallel design with two arms (N/2 in each arm) and rho is the correlation between responses within a single woman during different periods (intra-woman correlation).

For the adherence endpoint, we use preliminary data from HPTN 059 to estimate the standard deviation (SD) of daily gel use and make the assumption that the standard deviations of adherence to daily oral use and of adherence to dual use are similar to that for daily gel use. From the first 4 weeks and the first 8 weeks of daily gel use observed in HPTN 059, we have observed a SD of 11% and 16%, respectively (note that we do not have data in HPTN 059 for the first 6 weeks). Therefore, we use a conservative estimate for the SD for the first 6 weeks of 20%.

To our knowledge, there are no data available for estimating rho. However, it is highly likely that this correlation will be positive and large. The following table gives the power to detect various differences in adherence with a sample size of 144 evaluable women:

Table 13: Minimum Detectable Difference in Adherence

	Rho			
	**0.0**	**0.3**	**0.5**	**0.7**
Minimum Detectable Absolute Difference in Adherence Between Any Two Regimens				
2.5%	18%	24%	32%	49%
5.0%	56%	72%	85%	97%
7.5%	89%	97%	99%	>99%

If there is no intra-woman correlation for adherence, the study will have ≥89% power to detect absolute differences between the different study drug regimens of at least 7.5% with a two-sided alpha of 5%. Assuming a moderately high intra-woman correlation of 0.5 for adherence, the study will have ≥85% power to detect an absolute difference of at least 5.0% with a two-sided alpha of 5%.

For the acceptability endpoint, based on previous studies we expect to observe acceptability > 95%, which equates to ≤5% of women reporting they would be "unlikely" to use the study product in the future. Power is computed by using formulas appropriate for the McNemar test (formula 3.1 in Ezzet and Whitehead).[12] For the computations, we assume that the pooled acceptability for two regimens is 90% giving a probability of 18% that a woman has two different outcomes in two different periods (i.e. finds one product acceptable and the other not acceptable). Under this assumption, and assuming a 2-sided test with α=0.05, with 144 evaluable women we have 80% power to detect a log-odds ratio between acceptability rates in two groups of 1.10. If acceptability in one group is 95%, this corresponds to being able to detect a 13% difference (i.e., 5% versus 18% being "unlikely" to use study product in the future) in acceptability between different study product regimens. If acceptability in one group is 98%, this corresponds to being able to detect a 5% difference (i.e. 2% versus 7% being "unlikely" to use study product in the future) in acceptability between different study product regimens. Although the primary analysis for this endpoint will not rely on the McNemar test (see details in Section 10.7.2), the above computations provide a good approximation given that the range of individual acceptabilities observed in previous studies is rather homogenous. This leads to a small degree of heterogeneity; therefore negligible sample size adjustment is needed for the use of random effects model.

Finally, for the secondary endpoint assessing condom use rates during the different drug regimens, we use preliminary data from HPTN 035 to estimate the SD of percent condom use and make the assumption that the SDs of condom use for daily oral use and dual use are similar to that for daily gel use. From the first 2028 participants enrolled in HPTN 035 we have observed a condom use rate of 72% with a SD of 34% and we use this estimate of the SD for the power calculations.

Again, to our knowledge, there are no data available for estimating rho. However, it is highly likely that this correlation will be positive and large. The following table gives the

power to detect various differences in condom use rates with a sample size of 144 evaluable women:

Table 14: Minimum Detectable Difference in Condom Use

	Rho			
	**0.0**	**0.3**	**0.5**	**0.7**
Minimum Detectable Absolute Difference in Condom Use Between Any Two Regimens				
2.5%	9%	11%	14%	21%
5.0%	24%	32%	42%	62%
7.5%	46%	61%	75%	93%
10%	70%	85%	94%	>99%

If there is no intra-woman correlation for condom use, the study will have low power to detect absolute differences in condom use rates of <10% between the different study drug regimens. Assuming a moderately high intra-woman correlation of 0.5 for condom use, the study will have $\geq$75% power to detect an absolute difference of at least 7.5% with a two-sided alpha of 5%.

Methods for sample size estimates for the sparse sampling population PK analysis are not available. However, experience indicates that 144 participants, especially when each contributes a pair of specimens the same dosing interval, should provide robust PK parameter estimates.

The more intensive PK sampling to be done at the domestic sites will involve 72 women. Based on data from Gilead in the package insert[1], the coefficient of variation for C_{max}, AUC, and total clearance (Cl/F) are 30%, 30%, and 11%, respectively. With 72 participants, assuming no intra-individual correlation, we have 90% power to detect a 0.38 SD unit difference between any two regimens being compared. This represents a 14% difference in C_{max} and AUC and a 5% difference in total clearance (Cl/F). If one assumes some correlation within a participant between periods – a very reasonable assumption – these estimates would be even lower. However, because we are using fewer sample points than used in the Gilead tenofovir studies in which the parameter estimates and SDs were determined, we expect that this may increase the variability of the estimates, so we will make no adjustments for intra-participant correlations.

In HPTN 050, blood levels after vaginal tenofovir were too low to allow variance estimates needed for sample size calculations since only one-third of the women had detectable levels of tenofovir in their blood following any given dose. Comparisons of oral and dual formulation arms will rely on imputations of values that are below the assay limits of quantitation which will have the effect of actually reducing the variance of the data which may falsely increase the apparent power of the study design.

10.5 Randomization Procedures

Randomization to product sequence will be stratified by site. Within each site, participants will be randomly assigned to one of six study sequences outlined in Table 15: Study Regimen below. In an unblinded trial, special care needs to be taken to assure that the study staff cannot control or guess assignment. The MTN Statistical and Data Management Center will coordinate the randomization procedures, which will be specified in the SSP Manual.

Table 15: Study Regimen

	N	Period 1: 6 WEEKS	1 WK Wash-out	Period 2: 6 WEEKS	1 WK Wash-out	Period 3: 6 WEEKS	1 WK Wash-out
**Sequence A**	**24**	Oral		Vaginal		Oral + Vaginal	
**Sequence B**	**24**	Vaginal		Oral		Oral + Vaginal	
**Sequence C**	**24**	Oral + Vaginal		Oral		Vaginal	
**Sequence D**	**24**	Oral + Vaginal		Vaginal		Oral	
**Sequence E**	**24**	Oral		Oral + Vaginal		Vaginal	
**Sequence F**	**24**	Vaginal		Oral + Vaginal		Oral	

Assignment to end of study period non-intensive PK sample timing will correspond to product sequence randomization for simplicity. For the intensive PK cohorts at domestic sites, the end of study period sample timing will require a second randomization which will be stratified within each of the two sites.

10.6 Participant Accrual, Follow-up, Retention, and Replacement

The accrual period will be 6 months. Up to 204 women may be enrolled to reach the target of 144 evaluable women, where evaluable women are women who have at least one follow-up visit with adherence data in each of the 3 periods of the study. Five of the 7 participating sites will recruit and enroll 24 evaluable participants each, and 2 of the participating sites will enroll 12 evaluable participants each for a total of 144 evaluable participants. The need to enroll replacement participants for enrollees will be dependent on whether the enrollee had at least one full period of the study with no follow-up adherence data. Details on procedures for participant replacement are outlined in the SSP Manual.

Each participant will be followed for 21 consecutive weeks (three periods of 7 weeks). In a crossover study, it is important to have completeness of the data such that the target retention should be set at 100%. Therefore, once a participant has enrolled in the study, the study site will make every reasonable effort to retain her for the entire study period so that she is evaluable. A maximum of 5% loss-to-follow-up of enrolled participants is targeted.

10.7 Data Monitoring and Analysis

10.7.1 Study Monitoring Committee (SMC)

No Data and Safety Monitoring Board oversight is planned for this study. The MTN SMC will conduct interim reviews of study progress, including rates of participant accrual, retention, completion of primary and main secondary endpoint assessments, and study or lab issues. These reviews will take place approximately every 4-6 months, or as needed. At the time of these reviews, or at any other time, the SMC may recommend that the study proceed as designed, proceed with design modifications, or be discontinued.

10.7.2 Primary Analysis

All analyses will be based on data from the 144 evaluable women (women with at least one follow-up visit in each period of the study) after assessing differences in baseline values between the evaluable women and those women lost to follow-up. These differences will be described and used to interpret the generalizability of the results. For the adherence endpoint, the primary analysis will evaluate the difference in product adherence using statistical methods for paired data controlling for period and sequence effects. Prior to this analysis, the presence of any carryover effects will be evaluated using a more liberal alpha level of 10%. Although carryover effects are not expected, if evidence of them is found, we will resort to analyzing only the data from the first period as a simple three arm parallel design using statistical methods for independent data. This will greatly reduce the power of testing. If adherence distributions do not allow the use of parametric tests, rank tests as those described in Koch and Strokes will be used instead.[13]

For the binary acceptability endpoint, similar measures to assess for carryover effects will be used and then for the main outcome, controlling for period and sequence effects, a generalization of McCullagh's proportional odds model for repeated measures will be performed by adding a random effect on the logit scale as proposed by Ezzet and Whitehead.[14] Again, if carryover effects are present, the analysis will only use data from the first period.

Blood and intracellular samples will be analyzed for routine PK parameters - C_{max}, T_{max}, AUC, C_{min} - and described using descriptive statistics. Cervical and vaginal samples will be analyzed using sparse sampling population PK methods to provide PK parameter estimates. Measures of drug exposure (AUC, C_{max}) will be compared between oral and vaginal and dual use concentration differences controlling for sequence and period effects. Post-biopsy blood samples will be evaluated for deviation from expected values. Model building will be attempted to relate blood and intracellular model drug levels if sufficient samples have detectable drug levels.

11 DATA HANDLING AND RECORDKEEPING

11.1 Data Management Responsibilities

Study case report forms will be developed by the SDMC. Quality control reports and queries routinely will be generated and distributed by the SDMC to the study sites for verification and resolution.

11.2 Source Documents and Access to Source Data/Documents

Source documents and access to source data/documents will be maintained in accordance with the Requirements for Source Documentation in DAIDS Funded and/or Sponsored Clinical Trials. The investigator will maintain, and store securely, complete, accurate and current study records throughout the study. In accordance with US regulations, the investigator will retain all study records on site for at least two years after study closure. Study records will not be destroyed prior to receiving approval for record destruction from DAIDS. Applicable records include source documents, site registration documents and reports, correspondence, informed consent forms, and notations of all contacts with the participant.

11.3 Quality Control and Quality Assurance

Quality control and quality assurance procedures for MTN-001 will be performed in accordance with Requirements for Clinical Quality Management Plans at DAIDS Funded and/or Supported Clinical Research Sites.

11.4 Study Coordination

DAIDS holds the IND applications for this study. Assignment of all sponsor responsibilities for this study will be specified in a Clinical Trials Agreement executed by DAIDS, CONRAD, and Gilead Sciences, Inc. Study site staff will be provided with the DAIDS SOPs for Source Documentation and Essential Documents, the Manual for Expedited Reporting of Adverse Events to DAIDS, and the DAIDS AE Grading Table. Training and written instructions outlining management and reporting, study gel dispensing, product accountability, and other study operations will be provided by FHI, SCHARP, and the MTN NL.

12 CLINICAL SITE MONITORING

Study monitoring will be carried out by PPD (Wilmington, NC). On-site study monitoring will be performed in accordance with Requirements for On-Site Monitoring of DAIDS Funded and/or Sponsored Clinical Trials. Site monitoring visits will be conducted to assess compliance with Health and Human Services (HHS) Regulations 45 Code of

Federal Regulations (CFR) Part 46 and 21 CFR Parts 50, 56, and 312. Study monitors will visit the site to:

- Verify compliance with human subjects and other research regulations and guidelines, including confidentiality procedures, informed consent process, and regulatory documentation

- Assess adherence to the study protocol, SSP Manual, and local counseling practices

- Confirm the quality and accuracy of information collected at the study site and entered into the study database, including the validation of data reported on case report and DataFax forms

- Assess the resolution of any past or ongoing issues identified at previous monitoring visits

Site investigators will allow study monitors to inspect study facilities and documentation (e.g., informed consent forms, clinic and laboratory records, other source documents, case report forms), as well as observe the performance of study procedures. Investigators also will allow inspection of all study-related documentation by authorized representatives of the MTN CORE, MTN NL, FHI, SCHARP, NIAID, local regulatory authorities, and US regulatory authorities. A site visit log will be maintained at the study site to document all visits.

13 HUMAN SUBJECTS PROTECTIONS

The investigators will make efforts to minimize risks to human participants. Volunteers and study staff members will take part in a thorough informed consent process. Before beginning the study, the investigators will have obtained IRB/EC approval and the protocol will have been submitted to the FDA. The investigators will permit audits by the NIH, CONRAD, Gilead Sciences, Inc., the FDA, or any of their appointed agents.

13.1 Institutional Review Boards

Each participating institution is responsible for assuring that this protocol and the associated informed consent documents and study-related documents are reviewed by an EC or IRB prior to implementation of the protocol. Any amendments to the protocol, informed consents, or other study- related documents must be approved by the IRB/EC and DAIDS prior to implementation.

13.2 Protocol Registration

Each study site will complete protocol registration with the DAIDS RCC Protocol Registration Office. Protocol registration material can be sent electronically to

epr@tech-res.com. For questions regarding protocol registration, please call (301) 897-1707. For additional information, refer to the protocol registration documents located at http://rcc.tech-res.com/forms.htm.

Protocol registration must occur as a condition for site-specific study activation; no participants may be screened or enrolled in this study prior to obtaining protocol registration approval and completing all other study activation requirements. MTN CORE (FHI) staff will notify each study site when all activation requirements have been met by issuing a site-specific study activation notice. Study implementation may not be initiated until the activation notice is issued.

The study will be conducted in full compliance with the protocol. The protocol will not be amended without prior written approval by the Protocol Chair and DAIDS Medical Officer. All protocol amendments must be submitted to and approved by the relevant IRB/EC(s) and the RCC prior to implementing the amendment.

13.3 Risk Benefit Statement

13.3.1 Risks

Phlebotomy may lead to discomfort, feelings of dizziness or faintness, and/or bruising, swelling and/or infection. Pelvic examination may cause mild discomfort and/or vaginal bleeding or spotting. Disclosure of STI status may cause sadness or depression in volunteers. Participation in clinical research includes the risks of loss of confidentiality and discomfort with personal nature of questions.

For sub-study participants, vaginal biopsy carries the risk of discomfort or pain during the procedure and for a few hours afterwards. Participants may have mild vaginal spotting (bleeding) for one or two days, and will be instructed to avoid sexual intercourse until bleeding stops. Some temporary discomfort with sexual intercourse may occur if the biopsy areas are still healing. There is a small risk of infection and heavier bleeding. Participants will be instructed to contact the clinic if symptoms are bothersome, if heavy bleeding is noted (soaking through a pad or tampon in an hour or less) or if the participant develops any abnormal odor or discharge from the vagina.

The most common side effects associated with oral TDF in patients with HIV infection are nausea, headache, diarrhea, vomiting, asthenia, flatulence, abdominal distension/pain and anorexia. Less common side effects of TDF include kidney toxicities and low blood phosphate. Other side effects reported in the post-marketing period include weakness, pancreatitis, low blood phosphate, dizziness, shortness of breath, and rash. In animal studies, tenofovir has been associated with decreased bone mineral density. These effects have not been seen in those taking tenofovir tablets for up to one year.

Redistribution/accumulation of body fat including central obesity, dorsocervical fat enlargement (buffalo hump), peripheral wasting, facial wasting, breast enlargement, and

"cushingoid appearance" have been observed in persons receiving antiretroviral drugs. The mechanism and long-term consequences of these events are currently unknown. A causal relationship has not been established. These effects are not expected in this study involving a brief exposure to antiretroviral drug.

Administration of tenofovir gel intravaginally at 0.3% and 1% concentrations in the HPTN 050 Phase1 study resulted in minimal local irritation and little or no systemic adverse effects were identified.[8] Although 92% of participants reported at least 1 AE, 87% of those reported AEs were mild, and 70% of the AEs were limited to the genitourinary tract. Four severe AEs were reported, with only one, lower abdominal pain, thought to be product-related. The risks associated with tenofovir gel are believed to be less than those identified for systemic use. In the HPTN 050 Phase1 study of tenofovir gel, serum PK analysis in a subset of participants demonstrated that there is no clinically significant systemic toxicity. Fourteen of 25 women with PK results had low, but detectable, serum tenofovir levels.

Given that Phase 1 data demonstrates measurable plasma concentrations of tenofovir in some participants, participants with hepatitis B infection might be at risk for development of tenofovir resistant hepatitis B. However, participants with known hepatitis B infection will not be eligible for enrollment. It is not known what effect tenofovir gel could have on the HIV virus or HIV disease progression in HIV infected participants or their partners. There is a theoretical risk that tenofovir absorbed systemically from tenofovir gel could result in mutations of the HIV virus in participants who become infected with HIV during the study, or their partner, if the partner is infected with HIV. Limited resistance data from HPTN 050 show no new resistance mutations in plasma or CVL specimens after 14 days of tenofovir gel use. No participant had high level tenofovir mutations (e.g. K65R). Some of the possible side effects of the study gel are dryness, itching, burning, or pain in the genital area.

13.3.2 Benefits

Participation in this study likely will have no direct benefit to volunteers other than access to screening for RTIs/STIs and appropriate referral if RTIs/STIs are diagnosed. Some volunteers may have the opportunity to access expedient treatment and decreased morbidity due to early diagnosis and treatment of abnormalities in serology, blood count, liver or kidney function tests. Pap smear may offer the opportunity for early detection of a cervical and/or vaginal abnormality with expedient referral if an abnormality is detected. Lastly, the participant may appreciate the opportunity to contribute to the body of knowledge in the field of microbicide research.

13.4 Informed Consent Process

Written informed consent will be obtained from all potential study participants prior to the initiation of any study-related procedures. In obtaining and documenting informed consent, the investigators and their designees will comply with applicable local and

country-specific regulatory requirements and will adhere to Good Clinical Practices (GCP) and to the ethical principles that have their origin in the Declaration of Helsinki. Study staff must document the informed consent process in accordance with the Requirements for Source Documentation in DAIDS Funded and/or Sponsored Clinical Trials. A comprehension checklist will be used to assess participants' comprehension of the enrollment informed consent document. Participants are provided with copies of the informed consent forms if they are willing to receive them. Each study site is responsible for developing study informed consent forms for local use, based on the templates in Appendices VI, VII, and VIII that describe the purpose of screening and of the study, the procedures to be followed, and the risks and benefits of participation, in accordance with all applicable regulations. The study site also is responsible for translating the template forms into local languages, and verifying the accuracy of the translation by performing an independent back-translation.

Prior to the beginning of the trial, site investigators will have IRB/EC written approval of the protocol, informed consent forms, and any other study-related information to be provided to participants.

The informed consent process will give individuals all of the relevant information they need to decide whether to participate, or to continue participation, in this study. Potential research participants will be permitted to ask questions and to exchange information freely with the study investigators. Listed study investigators or their designees will obtain informed consent from potential study participants. The investigators will keep research participants fully informed of any new information that could affect their willingness to continue study participation.

Community input has been sought for the development of the sample informed consent forms. The informed consent process covers all elements of informed consent required by research regulations. In addition, the process specifically addresses the following topics of import to this study:

- The importance of adherence to the study visit and procedures schedule.
- The potential risks of study participation (and what do if such risks are experienced).
- The potential social harms associated with study participation (and what do if such harms are experienced).
- The real yet limited benefits of study participation.
- The distinction between research and clinical care.
- The right to withdraw from the study at any time.

13.5 Participant Confidentiality

All study procedures will be conducted in private, and every effort will be made to protect participant privacy and confidentiality to the extent possible. Each study site will establish a standard operating procedure for confidentiality protection that reflects the local study implementation plan (e.g., whether community-based visits will be

conducted) and the input of study staff and community representatives to identify potential confidentiality issues and strategies to address them. In addition to local considerations, the protections described below will be implemented at all sites.

All study-related information will be stored securely at the study site. All participant information will be stored in locked file cabinets in areas with access limited to study staff. Data collection, process, and administrative forms, laboratory specimens, and other reports will be identified by a coded number only to maintain participant confidentiality. All local databases will be secured with password-protected access systems. Forms, lists, logbooks, appointment books, and any other listings that link participant ID numbers to other identifying information will be stored in a separate, locked file in an area with limited access. Participants' study information will not be released without their written permission, except as necessary for monitoring (see Section 12).

The MTN has obtained a Certificate of Confidentiality from the US Department of Health and Human Services that will be applicable for this study. This Certificate protects study staff from being compelled to disclose study-related information by any US Federal, State or local civil, criminal, administrative, legislative or other proceedings. It thus serves to protect the identity and privacy of study participants. Since the Certificate cannot be enforced outside of the US, it will apply only to US site staff and participants.

13.6 Special Populations

This section outlines considerations made for the inclusion or exclusion of special populations in this study.

13.6.1 Pregnant Women

Participants who test positive for pregnancy at screening or enrollment visits will not be eligible to participate in this study. A urine pregnancy test will be performed on all women at all clinic visits, and participants who test positive will be taken off product. During the informed consent process, women will be informed that oral tenofovir disoproxil fumarate and tenofovir gel are not methods of contraception and that the effects of oral tenofovir disoproxil fumarate and tenofovir gel on a developing human fetus are unknown.

Oral TDF is classified by the FDA as a Pregnancy Category B drug. Animal studies have failed to demonstrate a risk to the fetus, but there are no adequate and well-controlled studies in pregnant women that have been completed to date.

All potential participants will be required by the Eligibility Criteria for Screening and Enrollment to be currently using a reliable method of contraception, such as hormonal contraception (except vaginal ring), intrauterine device, or sterilization. Women who become pregnant during the study period following randomization and exposure to

study product will discontinue product use and the PK assessments but will not be excluded from analysis.

13.6.2 Children

The NIH has mandated that children be included in research trials when appropriate. This study meets “Justifications for Exclusion” criteria for younger children as set forth by the NIH. Specifically, “insufficient data are available in adults to judge potential risk in children” and “children should not be the initial group to be involved in research studies.” Oral TDF is not currently approved for children under 18 years old. This study does not plan to enroll children under 18 years old.

13.7 Incentives

Pending IRB/EC approval, participants will be compensated for their time and effort in this study, and/or be reimbursed for travel to study visits, child care, and time away from work.

13.8 Communicable Disease Reporting

Study staff will comply with all applicable local requirements to report communicable diseases including HIV identified among study participants to local health authorities. Participants will be made aware of all reporting requirements during the study informed consent process.

13.9 Access to HIV-related Care

13.9.1 HIV Counseling and Testing

HIV pretest and post-test counseling will be provided to all potential study participants who consent to undergo HIV screening to determine their eligibility for this study. Participants must receive their HIV test results to take part in this study. Participants who have positive or indeterminate results will have standard post-test counseling as well as limited follow-up confirmatory testing provided by the study. Referral for additional counseling related to testing or diagnosis will occur if needed or requested by the participant.

13.9.2 Care for Participants Identified as HIV-Infected

Study staff will provide participants with their HIV test results in the context of post-test counseling. According to site SOPs, study staff will refer participants found to be HIV-infected to available sources of medical and psychological care, social support, and local research studies for HIV-infected women. At applicable sites, participants may be offered participation in MTN-015, the MTN Seroconverter Study.

13.10 Study Discontinuation

This study may be discontinued at any time by NIAID, the MTN, CONRAD, Gilead Sciences, Inc., the US FDA, the OHRP, other government or regulatory authorities, or site IRBs/ECs.

14 PUBLICATION POLICY

DAIDS and MTN policies and a Clinical Trial Agreement (CTA) between CONRAD, Gilead Sciences, Inc. and NIAID will govern publication of the results of this study. Any presentation, abstract, or manuscript will be submitted by the Investigator to the MTN Manuscript Review Committee, DAIDS, CONRAD, and Gilead Sciences, Inc., for review prior to submission.

15 APPENDICES

APPENDIX I: SCHEDULE OF STUDY VISITS AND EVALUATIONS

		PERIOD 1			PERIOD 2			PERIOD 3				
	SCR	ENR	3W	6W	7W	10W	13W	14W	17W	20W	21W	INT.
Informed Consent	X	X										
PTID	X											
Demographics	X											
Locator Information	X	X	X	X	X	X	X	X	X	X	X	X
Behavioral Eligibility	X	X										
Reimbursement	X	X	X	X	X	X	X	X	X	X	X	
Randomization		X										
Schedule Next Visit	X	X	X	X	X	X	X	X	X	X	*	*
Med./Menstrual History	X	X	X	X	X	X	X	X	X	X	X	X
Con. Meds.	X	X	X	X	X	X	X	X	X	X	X	X
Adverse Events			X	X	X	X	X	X	X	X	X	X
Pre-existing Conditions		X										
Physical Exam	X	X	X	X	X	X	X	X	X	X	*	*
Pelvic Exam	X	X	X	X	X	X	X	X	X	X	*	*
Contraceptive Coun.	X	X	X	X	X	X	X	X	X	X	X	*
Pre-/Post-Test Coun.	X	*			X			X			X	*
Protocol Adherence Coun.		X	X	X	X	X	X	X	X	X		*
HIV/STI Risk Red. Coun.	X	X	X	X	X	X	X	X	X	X	X	*
Product Use/Adherence		X	X		X	X		X	X			*
Male Condom Coun.	X	X	X	X	X	X	X	X	X	X	X	*
Treat and/or Refer	*	*	*	*	*	*	*	*	*	*	*	*
Behav. Assessment		X	X	X		X	X		X	X		
In-Depth Interview (subset)											X	
Qualitative Urine hCG	X	X	X	X	X	X	X	X	X	X	X	X
Dipstick Urinalysis	X	*	*	*	*	*	*	*	*	*	*	*
Urine Culture	*	*	*	*	*	*	*	*	*	*	*	*
Urine SDA for CT/GC	X	*	*	*	*	*	*	*	*	*	*	*
CBC	X	X			X			X			X	*
Liver Function	X	X	X	X	X	X	X	X	X	X	X	*
Serum Chemistries	X	X	X	X	X	X	X	X	X	X	X	*
Syphilis Serology	X	*	*	*	*	*	*	*	*	*	*	*
Confirmation Syphilis	*	*	*	*	*	*	*	*	*	*	*	*
HIV-1 Test	X	*			X			X			X	*
Confirmation HIV-1	*	*			*			*			*	*
HBsAg	X		*	*	*	*	*	*	*	*	*	*
Plasma for Storage		X		X			X			X	X	
Tenofovir			X	X		X	X		X	X		*
Insert Lock/Remove Lock				*			*			*		
Flow Cytometry				++			++			++		
Intracel.Ten./Ten. Diphos.				++			++			++		
Vaginal pH	X	X	*	X	*	*	X	*	*	X	*	*
Wet Mount	X	X	*	X	*	*	X	*	*	X	*	*
Herpes Culture	*	*	*	*	*	*	*	*	*	*	*	*
Pap Smear (if >12 months)	*											
CVL		X		X			X			X		
Provide Male Condoms	X	X	X	X	X	X	X	X	X	X	X	*
Offer Panty Liners		X	X	X	X	X	X	X	X	X		*
Supply Study Products		X	X		X	X		X	X			*
PK Dose Dispensing				X			X			X		
Collect Unused Prod.			X	X	*	X	X	*	X	X	*	*
Watch Device		X	*			*			*			*
Cytology Brush/Biopsies				●			●			●		

X Protocol specified proc., *If indicated, ● Intensive PK participants only, ++ At sites with capacity, 7.8 for time points

APPENDIX II: HIV ANTIBODY TESTING ALGORITHMS

Algorithm for HIV antibody testing – Screening (and Enrollment if applicable)

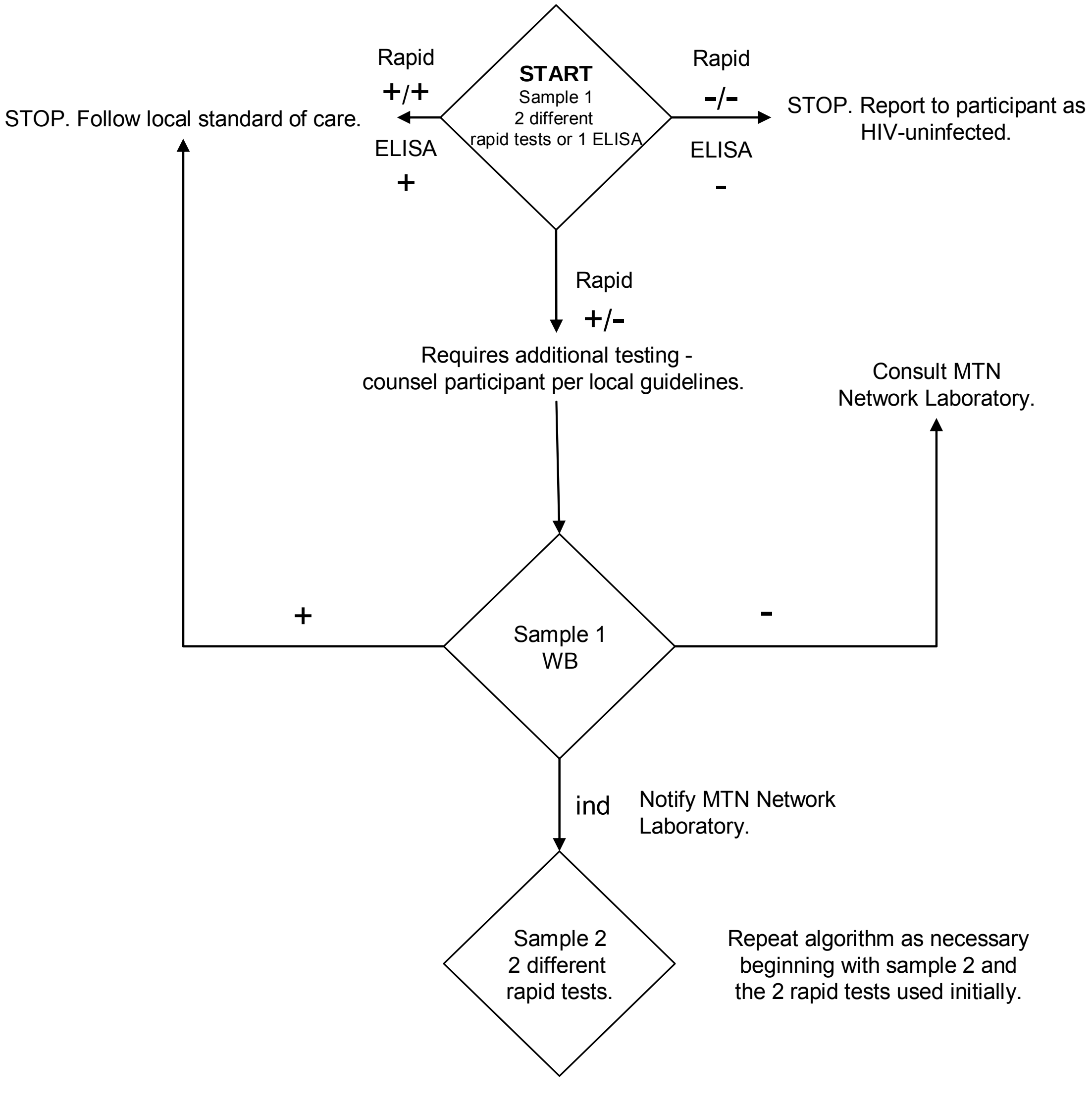

Algorithm for HIV antibody testing during follow-up

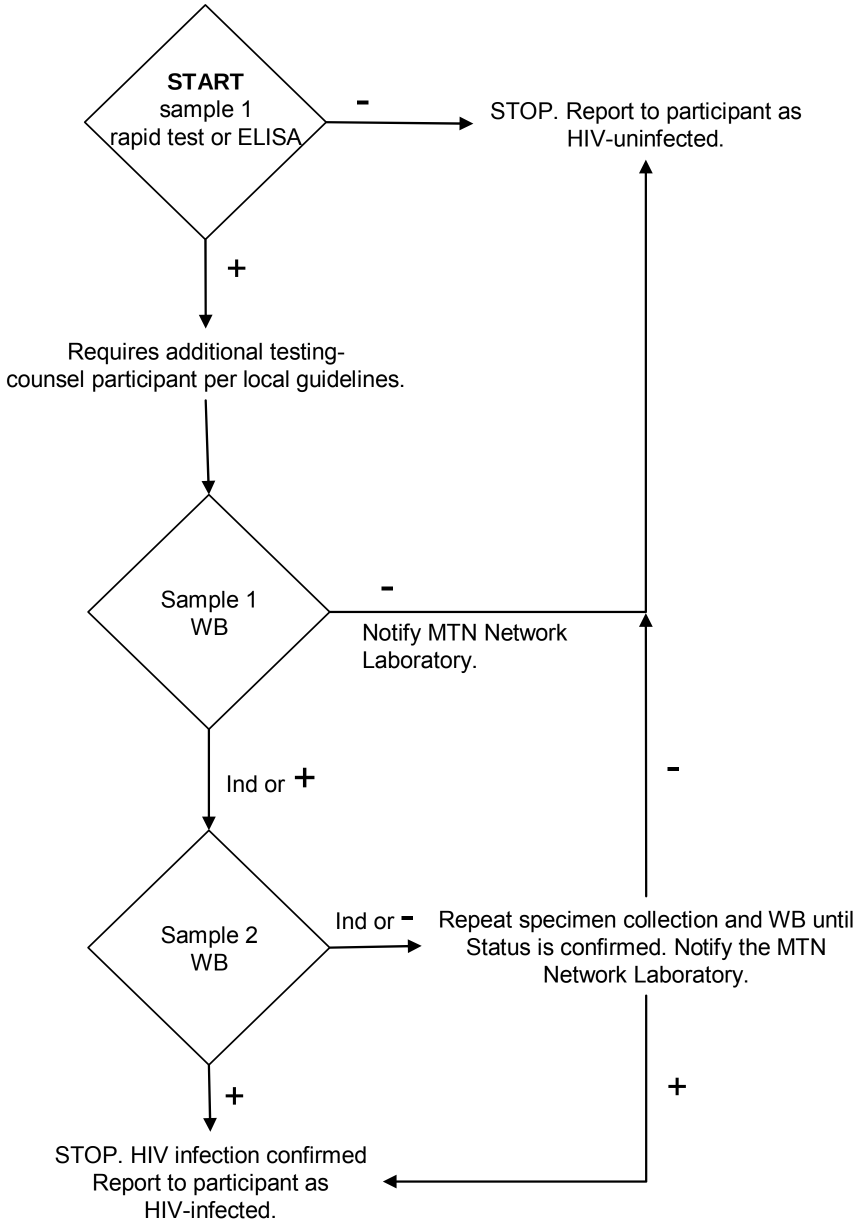

APPENDIX III: COMPONENTS OF EXAMINATIONS

Physical Exam

- Height (may be omitted after the Enrollment Visit)
- Weight (must be repeated with each Physical Exam)
- Vital signs
  - o Temperature
  - o Pulse
  - o Blood pressure
- General appearance
- Abdomen
- Other components as indicated by participant symptoms

Pelvic Exam

- Vulva
- Perianal area
- Speculum exam
  - o Vagina (including vaginal discharge)
  - o Cervix (including cervical discharge)
- Bimanual exam, if clinically indicated
  - o Cervix
  - o Uterus
  - o Adnexae

APPENDIX IV: TOXICITY TABLES

The Female Genital Grading Table for Use in Microbicide Studies will be the primary tool for grading adverse events for this protocol, with the exception of asymptomatic bacterial vaginosis which will not be a reportable AE. Adverse events not included in that table will be graded by the DAIDS AE Grading Table Version 1.0, December 2004. In cases where an AE is covered in both tables, the Female Genital Grading Table for Use in Microbicide Studies will be the grading scale utilized.

The Division of AIDS Table for Grading the Severity of Adult and Pediatric Adverse Events (DAIDS AE Grading Table), Version 1.0, December, 2004, is available on the RCC website at http://rcc.tech-res-intl.com/.

Female Genital Grading Table for Use in Microbicide Studies

INDIVIDUAL SIGNS/SYMPTOMS					
PARAMETER	GRADE 0 NORMAL	GRADE 1 MILD	GRADE 2 MODERATE	GRADE 3 SEVERE	GRADE 4 POTENTIALLY LIFE-THREATENING
**GENERAL**					
Odor	No complaint	Mild-moderate unpleasant odor	Severe unpleasant odor	NA	NA
**PAIN AND TENDERNESS (Specify Area: Vulvar/Perineum, Vagina, Cervix (including cervical motion tenderness), Uterus, Adnexae, Pelvic/Lower Abdominal, or Ovulatory)** *Note – if both pain and tenderness are present, only report the one with the most severe grade					
Pain* [1]	None	Pain causing no or minimal interference with usual social & functional activities	Pain causing greater than minimal interference with usual social & functional activities or the need for non-narcotic medication	Pain causing inability to perform usual social & functional activities or the need for narcotic medication	Disabling pain causing inability to perform basic self-care functions OR hospitalization (other than emergency room visit) indicated
Tenderness* [1]	None	Mild tenderness	Moderate tenderness	Severe tenderness	NA
Dyspareunia (pain with sexual activity)	None	Pain causing no or minimal interference with sexual function	Pain causing greater than minimal interference with sexual function	NA	NA
Dysmenorrhea/cramping with menses	None	Pain causing no or minimal interference with usual social & functional activities	Pain causing greater than minimal interference with usual social & functional activities or the need for non-narcotic medication	Pain causing inability to perform usual social or functional activities or the need for narcotic medication	NA

[1] If pain or tenderness is included in the grading of another category (e.g., PID), it should not be graded again in the pain or tenderness category.

Female Genital Grading Table for Use in Microbicide Studies

INDIVIDUAL SIGNS/SYMPTOMS					
**PARAMETER**	**GRADE 0 NORMAL**	**GRADE 1 MILD**	**GRADE 2 MODERATE**	**GRADE 3 SEVERE**	**GRADE 4 POTENTIALLY LIFE-THREATENING**
**GENITOURINARY SIGNS/SYMPTOMS – VULVA**					
Vulvar/vaginal itching	None	Itching causing no, mild, or moderate interference with usual social & functional activities	Itching causing inability to perform usual social & functional activities; may require intervention such as antihistamine or bathing to provide relief	NA	NA
Vulvar edema	None	Mild, non-pitting edema	Moderate, 1-2+ pitting edema	3+ pitting edema, severe enough to require urinary drainage, or weeping edema ± skin breakdown	NA
Vulvar erythema	None	Erythema covering < 50% of vulvar surface	Erythema covering ≥ 50% of vulvar surface	NA	NA
Vulvar lesions (findings seen only by colposcopy should not be included here)	Normal variants including skin tags, moles, scars, etc.	Blisters, ulcerations, or pustules - no treatment indicated	Blisters, ulcerations or pustules, with treatment indicated	Severe epithelial disruption with hospitalization indicated	NA
Vulvar rash	None	Rash covering < 50% of vulvar surface	Rash covering ≥ 50% of vulvar surface	Severe epithelial disruption with hospitalization indicated	NA
Bartholin's or Skene's gland	No findings	Cyst with no inflammation	Cyst or abscess with outpatient intervention indicated	Cyst or abscess with hospitalization indicated	Necrotizing fasciitis from Bartholin's abscess
**GENITOURINARY SIGNS/SYMPTOMS – VAGINA** ** Note – if vaginal discharge is present both by history and on examination, only report the one with the most severe grade					
Vaginal edema	None	Mild-moderate engorgement	Loss of ruggae and friability	NA	NA

Female Genital Grading Table for Use in Microbicide Studies

INDIVIDUAL SIGNS/SYMPTOMS					
**PARAMETER**	**GRADE 0 NORMAL**	**GRADE 1 MILD**	**GRADE 2 MODERATE**	**GRADE 3 SEVERE**	**GRADE 4 POTENTIALLY LIFE-THREATENING**
Vaginal erythema	None	Erythema covering < 50% of vaginal surface	Erythema covering ≥ 50% of vaginal surface	NA	NA
Vaginal dryness	No complaint	Dryness causing no or minimal interference with usual sexual, social, & functional activities	Dryness causing greater than minimal interference with usual sexual, social, & functional activities	NA	NA
Vaginal discharge by participant report **	Participant's usual amount of discharge, regardless of color or quantity	Mild-moderate increase in amount above participant baseline - no sanitary protection required	Profuse increase in discharge requiring pad use or other hygienic intervention	NA	NA
Vaginal discharge as observed by clinician ** (red or brown discharge should be reported under bleeding, not discharge)	Slight amount of discharge, any color	Mild-moderate increase in amount	Significant increase in amount with pooling in vagina on examination	NA	NA
Vaginal abrasions or lacerations (including probable applicator injuries)	None	Superficial disruptions and disruptions extending through the mucosa with minimal impact on life	Large disruptions extending through the mucosa or large superficial disruptions, hospitalization not indicated	Large disruptions extending through the mucosa or large superficial disruptions, hospitalization indicated	Lacerations extending into the peritoneal cavity, bladder, or rectum
Vaginal lesions (findings seen only by colposcopy should not be included here)	Normal variants including skin tags, moles, scars, etc.	Blisters, ulcerations, or pustules, no treatment indicated	Blisters, ulcerations, or pustules with treatment indicated	Severe epithelial disruption requiring hospitalization	NA
Vaginal and Cervical masses (polyps, myomas, or possible malignancy)	None or normal variants such as Nabothian cyst or Gartner duct cyst	Polyp or myoma or undiagnosed mass without symptoms	Polyp, myoma, or undiagnosed mass causing mild symptoms, e.g., bleeding/pain not requiring more than mild analgesia	Polyp, myoma, or undiagnosed mass causing severe symptoms, e.g., bleeding/pain affecting bladder and bowel function	Visible cervical cancer

Female Genital Grading Table for Use in Microbicide Studies

INDIVIDUAL SIGNS/SYMPTOMS					
**PARAMETER**	**GRADE 0 NORMAL**	**GRADE 1 MILD**	**GRADE 2 MODERATE**	**GRADE 3 SEVERE**	**GRADE 4 POTENTIALLY LIFE-THREATENING**
**GENITOURINARY SIGNS/SYMPTOMS – CERVIX**					
Cervical edema and friability	None	Edema without friability	Friable cervix	NA	NA
Cervical erythema	None	Erythema covering < 50% of cervix	Erythema covering ≥ 50% of cervix	NA	NA
Cervical discharge	White or clear discharge	Small amount of purulent discharge at os	Purulent discharge extending onto cervix or vagina	NA	NA
Visible cervical lesions (findings seen only by colposcopy should not be included here)	Normal variants including skin tags, moles, scars, etc.	Blisters, ulcerations, or pustules, no treatment indicated	Blisters, ulcerations, or pustules with treatment indicated	NA	NA
**GENITOURINARY SIGNS/SYMPTOMS – UTERUS**					
Uterine masses/enlargement based on bimanual examination	Normal to 8 week size, no palpable myomas	Enlarged uterus and mild symptoms, e.g., bleeding/pain requiring mild analgesics	Enlarged uterus/myoma with moderate pain or symptoms, e.g., bleeding	Mass causing severe bleeding/pain or with impact on bowel/bladder function	Uterine mass that requires transfusion or surgery
Polyp, submucosal fibroid, or thickened endometrium detected by transvaginal ultrasound (new or increasing in size from prior exam)	None or unchanged/reduced in size from prior exam	New myomas < 6 cm diameter (single or multiple) or diameter increased < 6 cm since prior exam	New myomas ≥ 6 cm diameter (single or multiple) or diameter increased ≥ 6 cm since prior exam	Hospitalization and/or surgery indicated	NA
**GENITOURINARY SIGNS/SYMPTOMS – ADNEXA**					
Not pregnancy- or infection-related adnexal masses based on bimanual exam (use if no ultrasound done; if ultrasound done, use ultrasound categories below)	None, ≤ 4 cm, normal size ovary	> 4 cm with minimal or no symptoms	> 4 cm with severe symptoms, e.g., pain, but hospitalization not indicated (see footnote #1)	> 4 cm with severe symptoms, e.g., pain and hospitalization indicated (see footnote #1)	NA

Female Genital Grading Table for Use in Microbicide Studies

INDIVIDUAL SIGNS/SYMPTOMS					
PARAMETER	GRADE 0 NORMAL	GRADE 1 MILD	GRADE 2 MODERATE	GRADE 3 SEVERE	GRADE 4 POTENTIALLY LIFE-THREATENING
Hydrosalpinx based on ultrasound	None	Asymptomatic, suspected hydrosalpinx	Hydrosalpinx with pain, but without evidence of infection or ectopic pregnancy	Signs/symptoms of infection with hospitalization and/or surgery indicated	NA
Adnexal mass based on ultrasound	None	Simple cyst, asymptomatic	Simple cyst, symptomatic	Mass suspicious for malignancy	Malignant mass
**GENITOURINARY SIGNS/SYMPTOMS – ABDOMEN**					
Abdominal mass not palpable on pelvic exam of unknown diagnosis	None or known (pre-existing) mass unchanged in size	New mass or increased size of known mass requiring mild analgesia with minimal impact	New mass or increased size of known mass with moderate symptoms	Mass causing severe bleeding/ pain with impact on bladder/bowel function or with hospitalization indicated	Malignancy
**GENITOURINARY SIGNS/SYMPTOMS – URINARY TRACT**					
Urinary frequency	None	Up to 2 times participant's normal frequency	> 2 times participant's normal frequency	NA	NA
Dysuria	None	Superficial only	Deep ± superficial	Inability to void due to pain	NA
Hematuria	None	Microscopic, no intervention indicated (beyond evaluation for infection)	Gross blood in urine or medical intervention/ evaluation indicated (beyond evaluation for infection)	Persistent bleeding with transfusion, hospitalization or intervention indicated to obtain hemostasis (endoscopy, interventional radiology, or operative)	Profuse hemorrhage with shock or orthostatic dizziness

Female Genital Grading Table for Use in Microbicide Studies

COMPOSITE SIGNS/SYMPTOMS (Use instead of individual categories if 2 or more signs/symptoms are present)					
PARAMETER	GRADE 0 NORMAL	GRADE 1 MILD (Use if all signs/ symptoms would individually be Grade 0 or 1)	GRADE 2 MODERATE (Use if one or more signs/symptoms would individually be Grade 2 and all others Grade 0 or 1)	GRADE 3 SEVERE (Use if one or more signs/symptoms would individually be Grade 3)	GRADE 4 POTENTIALLY LIFE-THREATENING
**NO ORGANISM IDENTIFIED BUT INADEQUATE TESTING PERFORMED**					
Vulvovaginitis (combinations of pain, itching, erythema, edema, rash, tenderness, or discharge)	None	Mild signs/ symptoms	Moderate signs/ symptoms	Severe signs/ symptoms	NA
Cervicitis (combinations of dyspareunia, erythema, edema, tenderness, and discharge)	None	Mild signs/ symptoms	Moderate signs/ symptoms	Severe signs/ symptoms	NA
PID (if Gonorrhea or Chlamydia identified use that category)	None	NA	Cervicitis with mild uterine tenderness, ± mild cervical motion tenderness, no signs of peritoneal irritation	More diffuse tenderness, any signs of peritoneal irritation, or indications for hospitalization	Tubo-ovarian abscess or surgery required for resolution
**NO ORGANISM IDENTIFIED AFTER APPROPRIATE TESTING PERFORMED**					
Vulvovaginitis (combinations of pain, itching, erythema, edema, rash, tenderness, or discharge)	None	Mild signs/ symptoms	Moderate signs/ symptoms	Severe signs/ symptoms	NA
Cervicitis (combinations of dyspareunia, erythema, edema, tenderness, and discharge)	None	Mild signs/ symptoms	Moderate signs/ symptoms	Severe signs/ symptoms	NA
PID (if Gonorrhea or Chlamydia identified use that category)	None	NA	Cervicitis with mild uterine tenderness, ± mild cervical motion tenderness, no signs of peritoneal irritation	More diffuse tenderness, any signs of peritoneal irritation, or indications for hospitalization	Tubo-ovarian abscess or surgery required for resolution

Female Genital Grading Table for Use in Microbicide Studies

INFECTIONS AND DYSPLASIA					
PARAMETER	GRADE 0 NORMAL	GRADE 1 MILD	GRADE 2 MODERATE	GRADE 3 SEVERE	GRADE 4 POTENTIALLY LIFE-THREATENING
**GENITOURINARY INFECTIONS**					
Genital herpes	No lesions	Characteristic ulcerative or vesicular lesions confirmed by culture, PCR, Tzanck prep or other diagnostic test of lesion or previous type-specific serology, covering < 25% of vulva, vagina, or cervix	Same criteria as mild but covering 25-50% of vulvar, vaginal, or cervical surface	Same criteria as mild but covering > 50% of vulvar, vaginal, or cervical surface	Symptoms of significant systemic involvement, e.g., encephalitis, hepatitis
Candida	Absence of symptoms regardless of candida test results	Positive culture, wet mount, or other laboratory test for yeast, with mild symptoms	Positive culture, wet mount, or other laboratory test for yeast, with moderate to severe symptoms	NA	NA
Trichomonas	Negative	NA	Positive wet mount, culture, PCR or other licensed test, excluding pap smear, showing T. vaginalis, regardless of symptoms	NA	NA
Bacterial Vaginosis (BV)	Negative	Asymptomatic BV diagnosed by Amsel criteria, wet mount, Gram stain, or licensed diagnostic test	Symptomatic confirmed by wet mount, Gram stain, or any licensed diagnostic test	NA	NA

Female Genital Grading Table for Use in Microbicide Studies

INFECTIONS AND DYSPLASIA					
PARAMETER	GRADE 0 NORMAL	GRADE 1 MILD	GRADE 2 MODERATE	GRADE 3 SEVERE	GRADE 4 POTENTIALLY LIFE-THREATENING
Chlamydia	Negative	NA	Positive culture or other diagnostic test for Chlamydia, asymptomatic or with mild uterine or cervical motion tenderness (no signs of peritoneal irritation)	Positive test for Chlamydia with abdominal or uterine or adnexal tenderness on examination, with or without adnexal mass, diffuse tenderness, any signs of peritoneal irritation, or indications for hospitalization	Tubo-ovarian abscess or surgery required for resolution
Gonorrhea	Negative	NA	Positive culture or other diagnostic test for Gonorrhea, asymptomatic or with mild uterine or cervical motion tenderness (no signs of peritoneal irritation)	Positive test for Gonorrhea with abdominal or uterine or adnexal tenderness on examination, with or without adnexal mass, diffuse tenderness, any signs of peritoneal irritation, or indications for hospitalization	Tubo-ovarian abscess or surgery required for resolution or disseminated gonococcal infection
Urinary tract infection (by urinalysis and urine culture)	Negative	5-10 WBC/hpf on urinalysis with a negative culture per protocol definition (with or without symptoms)	> 10 WBC/hpf on urinalysis OR a positive culture per protocol definition (with or without symptoms)	Pyelonephritis	Sepsis (septicemia) due to urinary tract infection

Female Genital Grading Table for Use in Microbicide Studies

INFECTIONS AND DYSPLASIA					
**PARAMETER**	**GRADE 0 NORMAL**	**GRADE 1 MILD**	**GRADE 2 MODERATE**	**GRADE 3 SEVERE**	**GRADE 4 POTENTIALLY LIFE-THREATENING**
Syphilis	Negative treponemal or non-treponemal test or both positive with known treatment and stable titers (< 4 fold increase)	NA	Syphilis diagnosed by a positive treponemal test along with a positive non-treponemal test and no previous treatment or a four-fold rise in titer on the non-treponemal test after previous treatment regardless of symptoms or non-oral lesions positive by darkfield exam for treponemes	Criteria for Grade 2 Syphilis in the presence of neurologic symptoms or a positive CSF VDRL or FTA-ABS	NA
**GENITAL DYSPLASIA**					
Condyloma (specify site: cervical, vaginal, vulvar, perianal)	None	Condylomata causing no or mild interference with daily function	Condylomata causing moderate interference with daily function	Condylomata causing severe interference with daily function, secondary infection, or hospitalization indicated	NA
Intraepithelial Neoplasia by biopsy (VIN, CIN, VAIN)	None	Intraepithelial Neoplasia 1 (IN1)	Intraepithelial Neoplasia 2 (IN2)	Carcinoma in situ (CIS)	Invasive carcinoma
Pap (use this category only if treatment performed without diagnostic testing, otherwise use biopsy category above)	nl PAP	ASCUS or LSIL	HSIL	Carcinoma in situ or Carcinoma	NA

Female Genital Grading Table for Use in Microbicide Studies

UTERINE BLEEDING AND PREGNANCY COMPLICATIONS					
**PARAMETER**	**GRADE 0 NORMAL**	**GRADE 1 MILD**	**GRADE 2 MODERATE**	**GRADE 3 SEVERE**	**GRADE 4 POTENTIALLY LIFE-THREATENING**
**ABNORMAL UTERINE BLEEDING UNRELATED TO PREGNANCY**					
Menorrhagia [2] (prolonged and/or heavy menstrual bleeding)	Participant report of normal bleeding relative to her baseline	Increase from usual with no or minimal interference with usual social & functional activities (including sexual functioning)	Increase from usual with moderate interference with usual social & functional activities (including sexual)	Incapacitating or severe interference with usual social & functional activities (including sexual functioning), transfusion indicated	Life threatening hemorrhage with or without shock
Metrorrhagia [2] (intermenstrual or frequent bleeding)	None or any expected nonmenstrual bleeding	Increase from usual with no or minimal interference with usual social & functional activities (including sexual functioning)	Increase from usual with moderate interference with usual social & functional activities (including sexual)	Incapacitating or severe interference with usual social & functional activities (including sexual functioning), transfusion indicated	Life threatening hemorrhage with or without shock
Unexplained infrequent bleeding (excludes expected absence of menses due to hormonal contraception or pregnancy/postpartum)	Participant report of normal or expected bleeding frequency	No menses for 1-3 months (missed menses)	No menses for > 3 months (oligomenorrhea/ amenorrhea)	NA	NA
Postcoital bleeding	None	Occasional (< 25% of coital acts) OR Increase from usual with no or minimal interference with usual social functioning (including sexual functioning)	Frequent (25-75% of coital acts) OR Increase from usual with moderate interference with usual social functioning (including sexual)	Consistent (> 75% of coital acts) OR Incapacitating or severe interference with usual social functioning (including sexual functioning), transfusion indicated	Life threatening hemorrhage with or without shock

[2] If both Menorrhagia and Metrorrhagia are present, a single adverse event should be reported as "Menometrorrhagia" and graded per the Menorrhagia grading scale.

Female Genital Grading Table for Use in Microbicide Studies

UTERINE BLEEDING AND PREGNANCY COMPLICATIONS					
PARAMETER	GRADE 0 NORMAL	GRADE 1 MILD	GRADE 2 MODERATE	GRADE 3 SEVERE	GRADE 4 POTENTIALLY LIFE-THREATENING
COMPLICATIONS OF PREGNANCY					
First trimester bleeding	None	Spotting or bleeding less than menses with continuation of pregnancy	Bleeding like menses or heavier with continuation of pregnancy	Spontaneous abortion, or profuse bleeding with dizziness or orthostatic hypotension, transfusion indicated	Spontaneous abortion with profuse bleeding and/or shock
Postabortal endometritis/salpingitis	None	Low grade fever and uterine tenderness, resolved with oral antibiotics	Moderate symptoms, requiring ≤ 3 days of parenteral antibiotics	Severe symptoms requiring > 3 days of IV antibiotics or development of tubo-ovarian abscess	Ruptured TOA or diffuse peritonitis or severe uterine infection for which operative intervention indicated
Postpartum hemorrhage	EBL < 500 cc for vaginal delivery or < 1000 cc after CS or reported as normal	EBL 500-1000 for vaginal delivery or 1000-1500 for CS or reported as slightly increased	EBL > 1000 for vaginal delivery or > 1500 for CS, with or without mild dizziness, no transfusion required	Hemorrhage at a level for which transfusion of 1-2 units of packed cells, but no other blood products indicated	Hemorrhage with shock or coagulopathy, for which transfusion of > 2 units of packed cells or any amount of other blood components is indicated
Postpartum endometritis	None	Low grade fever and uterine tenderness, resolved with oral antibiotics	Moderate symptoms, treated by ≤ 3 days of parenteral antibiotics	Severe symptoms treated with > 3 days of IV antibiotics or addition of heparin	Severe infection or infection for which operative intervention is indicated
Chorioamnionitis	None	Fever (38°C – 38.4°C or 100.4°F – 100.9°F) with two or more: FHR > 160 BPM, maternal HR > 120, uterine tenderness between contractions or purulent AF or preterm labor	Same as Grade 1 plus fever 38.5°C – 40°C or 101°F – 104°F	Criteria for Grade 2 plus fetal distress or fever > 40°C or 104°F	Criteria for Grade 3 plus either fetal demise or maternal symptoms of shock

Female Genital Grading Table for Use in Microbicide Studies

UTERINE BLEEDING AND PREGNANCY COMPLICATIONS					
**PARAMETER**	**GRADE 0 NORMAL**	**GRADE 1 MILD**	**GRADE 2 MODERATE**	**GRADE 3 SEVERE**	**GRADE 4 POTENTIALLY LIFE-THREATENING**
Episiotomy infection	None	Mild erythema, edema, and tenderness of wound	Fever > 38°C or 100.4°F with erythema, edema, and tenderness of wound	Fever with wound dehiscence or debridement required	Fever with signs of wound infection and shock or necrotizing fasciitis
Second/third trimester bleeding	None	Bleeding less than menses	Bleeding like menses or greater, but not requiring intervention	Bleeding requiring delivery or other intervention, e.g., transfusion	Bleeding with fetal demise or coagulopathy
Preterm rupture of membranes	None	NA	Preterm rupture with hospitalization but not resulting in delivery at less than 37 weeks' gestation	Delivery at 33-36 weeks' gestation or 1501-2500 grams birth weight	Delivery < 33 weeks' gestation or ≤ 1500 grams birth weight
Preterm contractions	None	Preterm contractions which resolve without medical intervention	Preterm contractions with cervical change which result in medical intervention but not resulting in preterm delivery	Delivery at 33-36 weeks' gestation or 1501-2500 grams birth weight	Delivery < 33 weeks' gestation or ≤ 1500 grams birth weight
Poor fetal growth	At or above 10th percentile	Fetal growth < 10th percentile but ≥ 3rd percentile for gestational age by ultrasound or newborn exam	NA	Fetal growth < 3rd percentile for gestational age by ultrasound or newborn exam	NA

APPENDIX V: MANUAL FOR EXPEDITED REPORTING OF ADVERSE EVENTS TO DAIDS

The Manual for Expedited Reporting of Adverse Events to DAIDS, Final 1.0, 6 May 2004 is available at: http://rcc.tech-res.com/eae.htm

APPENDIX VI: SAMPLE INFORMED CONSENT FORM (SCREENING)

Phase 2 Adherence and Pharmacokinetics Study of Oral and Vaginal Preparations of Tenofovir

Version 2.0
03 September 2008

**PRINCIPAL INVESTIGATOR:**	**[insert]**
**PHONE:**	**[insert]**
**Short Title for the Study:**	Adherence and Pharmacokinetics Study of Oral and Vaginal Tenofovir

INTRODUCTION

You are being asked to take part in these screening exams and tests because you are a woman between the ages of 18 and 45 years, and you may be able to join the research study named above. This Microbicide Trials Network (MTN) study is sponsored by the US National Institutes of Health (NIH). The person in charge of this study at this site is [INSERT NAME OF PRINCIPAL INVESTIGATOR]. The screening exams and tests include interview questions, urine and blood tests, a physical exam, and an examination of your vagina.

This is a screening consent form. It gives you information about the screening exams and tests. The study staff will explain the exams and tests to you and what is expected of you. You are free to ask questions about the screening exams and tests at any time. If you agree to have the screening exams and tests, you will be asked to sign or make your mark on this consent form in front of a witness. You will be given a copy of this form to keep.

Please note that your participation in this research is entirely voluntary. You may decide to withdraw from the study at any time without losing the benefits of your standard medical care.

WHY ARE THE SCREENING EXAMS AND TESTS BEING DONE?

These exams and tests are being done to see if you can be in this study. The exams and tests will check to see if you are eligible for the study.

WHAT IS THE PURPOSE OF THE STUDY?

This study is being done to understand women's experiences in taking a product called tenofovir several different ways; by experiences we mean safety and how women feel about taking the product. Women in this research study will take tenofovir tablets by mouth once a day for six weeks, tenofovir gel by vagina once a day for six weeks, and both products (oral plus gel) once a day for six weeks. The study will also do different tests on blood and genital specimens to see how much tenofovir goes to those areas of

the body. For example, we will measure how much tenofovir passes from the gel and goes into the blood. We are studying the "pharmacokinetics" of tenofovir; pharmacokinetics is what the body does to a drug, such as where the drug passes and in what amounts.

WHAT DO I HAVE TO DO IF I TAKE PART IN THE SCREENING EXAMS AND TESTS?

The Screening Visit will take about two hours. You will be asked to do these things if you decide you want to be in the study:

- Sign or make your mark on this form after you have read it or had it explained to you and had the chance to ask questions about the study
- Answer questions about yourself, such as where you live, your education, your behavior, including your sexual behavior, your medical history, menstrual period history, and any medicines that you may take and how we can contact you
- Have a physical exam and pelvic exam
- Hear about
    - different ways to avoid getting pregnant
    - how to avoid infections passed during sex
    - the meaning of your test results, including your HIV test results
    - how to use male condoms
- Get treatment for any infections passed during sex or urinary tract infection that you may have, or find out from the study staff where you can get care or treatment
- Provide a urine sample to get tested for pregnancy, urinary tract infection, and chlamydia and gonorrhea
- Provide a blood sample (about 11mL) [SITES TO INSERT LOCAL EQUIVALENT] to check these things:
    - The health of your blood, liver, and kidneys
    - HIV test, Hepatitis B test, and a syphilis test
- Provide a few drops of vaginal fluid to get tests for bacterial vaginosis, vaginal yeast infection, and Trichomonas
- [AT APPLICABLE SITES] Have a genital swab for herpes if you have signs of herpes
- Provide some cells from your cervix for a Pap smear if you do not have results with you today of a Pap smear that was done in the past 12 months (a Pap smear is a test for cervical cancer). If your Pap result is not normal, you might not be able to be in the study; the study staff can discuss this with you.
- Receive male condoms from the study staff
- It will take about [INSERT LENGTH OF TIME] to get the results of your screening tests. We will give you the results of these tests when they are available.

WHY WOULD THE DOCTOR STOP THE SCREENING PROCEDURES EARLY?

The study doctor may need to stop the screening exams and tests early without your permission if:

- The study is cancelled by the US Food and Drug Administration (FDA), US National Institutes of Health (NIH), the MTN, the drug company supporting this study, the Ethics Committees, the US Office for Human Research Protections, the local government or regulatory agency, or the Institutional Review Board (IRB). (An IRB is a committee that watches over the safety and rights of research participants).
- Your exams, tests and answers to the questions show you cannot join the study.
- The study staff feels that having the screening exams and tests would be harmful to you.
- You do not want to find out your HIV test result.
- You are not able to come to the visits or complete the screening exams and tests.
- Other reasons that may prevent you from completing the study.

WHAT ARE THE RISKS OF THE SCREENING VISIT TESTS?

Risk of Blood Draws:

- You may feel discomfort or pain when your blood is drawn.
- You may feel dizzy, faint or lightheaded.
- You may have a bruise, swelling, or infection where the needle goes into your arm.

Risk of Genital Exams:

- You may feel discomfort or pressure during the exam of your genital area and inside your vagina.
- You may have mild vaginal bleeding (spotting). The mild bleeding will stop shortly after the exam.

Other Possible Risks:

- You may become embarrassed, worried, or nervous when discussing personal questions about your sexual behavior, ways to protect against HIV and other infections passed during sex, and your test results.
- You may become worried or nervous while waiting for your test results.
- If you have HIV or other infections, knowing this could make you worried or nervous. A trained counselor will help you deal with any feelings or questions you have.

We will make every effort to protect your privacy while you are having the screening exams and tests. Your visits here will take place in private. However, it is possible that others may learn that you are taking part in the study here. Because of this, they may treat you unfairly. For example, you could have problems getting or keeping a job, or being accepted by your family or community.

ARE THERE BENEFITS TO TAKING PART IN THIS STUDY?

You may get no direct benefit from the screening exams and tests. However, you may benefit from the following:

- Physical exam and a pelvic exam

- Tests for sexually transmitted infections, other vaginal infections, and HIV (which may detect infections that have no symptoms). If you have any of these infections, you will be referred for treatment if needed. You can bring your male partner(s) here so that we can also provide them with referral for diagnosis and treatment for potential STIs.
- Tests to check your general health and the health of your liver, kidneys, and blood. This study cannot provide you with medical care, but study staff will refer you to other available sources of care.
- A Pap test if you have not had one in the past 12 months. If your Pap test result is not normal, you will be referred for treatment at the [INSERT NAME OF PROVIDER/CENTER].
- Safer sex counseling and free male condoms
- If your tests show that you are infected with HIV, you will be referred for medical care, counseling, and other services available to you. Medical care for HIV infection will not be part of this study. You will need to get medical care for your HIV infection from your own health care provider or we will provide you with a referral to a center where you can receive care. We will help you to access the right treatment for HIV infection if you need it.

WHAT OTHER CHOICES DO I HAVE BESIDES THIS STUDY?

You do not have to participate in this study, if you choose not to do so. [SITES TO INCLUDE/AMEND THE FOLLOWING IF APPLICABLE: There may be other studies going on here or in the community for which you may be eligible. If you wish, we will inform you about other studies that are being conducted locally. There also may be other places where you can go for HIV counseling and testing. We will tell you about those places if you wish.] Please talk to your doctor about these and other choices that may be available to you.

WHAT ABOUT CONFIDENTIALITY?

This study is being conducted according to ethical guidelines and efforts will be made to keep your personal information private. Your physical and vaginal exams will be done in private. We cannot guarantee absolute confidentiality. In some situations, including emergencies, legal and professional rules may force us to share confidential information about you. If this study is published, your name will not be used and you will not be personally identified. You are encouraged but not required to tell sexual partners about your being in this study.

Your records may be reviewed by:

- The US Food and Drug Administration (FDA)
- US National Institutes of Health (NIH)
- US Office for Human Research Protections (OHRP)
- Local regulatory authorities
- [INSERT NAME OF SITE] IRB
- Study staff
- Study monitors
- Ethics committees

- The companies that make the gel and the tablets

[SITES TO INCLUDE/AMEND THE FOLLOWING IF APPLICABLE:]
[LOCAL/STATE/NATIONAL] regulations require study staff to report the names of people who test positive for HIV and other infections passed during sex to the [LOCAL HEALTH AUTHORITY]. Outreach workers from the [HEALTH AUTHORITY] may then contact you about informing your partners, since they also should be tested. If you do not want to inform your partners yourself, the outreach workers will contact them, according to the confidentiality guidelines of the [HEALTH AUTHORITY].

[For US sites only:] In addition to the efforts made by the study staff to keep your personal information confidential, a Certificate of Confidentiality has been requested from the US Federal Government for this study. This Certificate protects study staff from being forced to tell people who are not connected with this study, such as the court system, about your participation or information you give for study purposes. Even with the Certificate of Confidentiality, however, if the study staff learns of possible child abuse and/or neglect or a risk of harm to you or others, they will be required to tell the proper authorities. Having a Certificate of Confidentiality does not prevent you from releasing information about yourself and your participation in the study.

WHAT ARE THE COSTS TO ME?
There is no cost to you for the screening exams and tests.

WILL I RECEIVE ANY PAYMENT?
You will be paid for your time and effort for each screening visit. You will receive [INSERT SITE - SPECIFIC AMOUNT OF MONEY] for each visit. You will also be paid for other costs to you for coming to the screening visits [SUCH AS CHILD CARE, TRAVEL, AND LOSS OF WORK TIME – SITES TO COMPLETE]. There may be one or more screening visits.

WHAT HAPPENS IF I AM INJURED (EXPERIENCE HARM)?
If you are injured as a result of being in this study, the [INSTITUTION] will give you immediate treatment for your injuries. You [will/will not] have to pay for this treatment. You will be told where you can get additional treatment for your injuries. The US National Institutes of Health (NIH) does not have a program to provide money or other forms of compensation for your injuries. Signing this consent form does not change your legal rights.

[SITES TO SPECIFY INSTITUTIONAL POLICY]

WHAT ARE MY RIGHTS AS A RESEARCH PARTICIPANT?
Taking part in the screening exams and tests is completely voluntary. You may choose not to have the screening exams and tests any time. You will be treated the same no matter what you decide. If you choose to not have the screening exams and tests, you will not lose the benefit of services to which you would normally have at this clinic.

We will tell you about new information from this or other studies that may affect your health, welfare or willingness to stay in this study. If you want the results of the study, let the study staff know.

WHAT DO I DO IF I HAVE PROBLEMS OR QUESTIONS?

For questions about the screening exams and tests or if you have a research-related injury, you should contact:

- [SITE INSERT NAME OF THE INVESTIGATOR OR OTHER STUDY STAFF]
- [SITE INSERT TELEPHONE NUMBER AND PHYSICAL ADDRESS OF ABOVE]

For questions about your rights as a research participant, contact:

- [SITE INSERT NAME OR TITLE OF PERSON ON THE INSTITUTIONAL REVIEW BOARD (IRB) OR OTHER ORGANIZATION APPROPRIATE FOR THE SITE]
- [SITE INSERT TELEPHONE NUMBER AND PHYSICAL ADDRESS OF ABOVE]

SIGNATURE
[INSERT SIGNATURE BLOCKS AS REQUIRED BY LOCAL IRB]

If you have read the informed consent (or had it read and explained to you), and all your questions have been answered and you agree to take part in this study, please sign your name or make your mark below.

__________________________<br>Participant's Name (print)	______________________________<br>Participant's Signature or Mark and Date
__________________________<br>Study Staff Conducting<br>Consent Discussion (print)	______________________________<br>Study Staff Signature and Date
__________________________<br>Witness' Name (print)<br>(As appropriate)	______________________________<br>Witness's Signature and Date

APPENDIX VII: SAMPLE INFORMED CONSENT DOCUMENT (ENROLLMENT)

Phase 2 Open Label Adherence and Pharmacokinetics Study of Oral and Vaginal Preparations of Tenofovir

Version 2.0
03 September 2008

**PRINCIPAL INVESTIGATOR:**	**[insert]**
**PHONE:**	**[insert]**
**Short Title for the Study:**	Adherence and Pharmacokinetics Study of Oral and Vaginal Tenofovir

INTRODUCTION

You are being asked to take part in this research study because you are a woman between the ages of 18 and 45 years and have passed the screening for this study. This Microbicide Trials Network (MTN) study is sponsored by the US National Institutes of Health (NIH). The person in charge of this study at this site is [INSERT NAME OF PRINCIPAL INVESTIGATOR]. Before you decide if you want to join this study, we want you to know about the study.

This is an enrollment consent form. It gives you information about this study. The study staff will talk with you about this information. You are free to ask questions about this study at any time. If you agree to take part in this study, you will be asked to sign or make your mark on this consent form in front of a witness. You will be given a copy of this form to keep. Please note that your participation in this research is entirely voluntary. You may decide to withdraw from the study at any time without losing the benefits of your standard medical care.

WHY IS THIS STUDY BEING DONE?

This study is being done to understand women's experiences in taking a product called tenofovir several different ways; by experiences we mean safety and how women feel about taking the product. Women in this research study will take tenofovir tablets by mouth once a day for six weeks, tenofovir gel by vagina once a day for six weeks, and both products (oral plus gel) once a day for six weeks. The study will also do different tests on blood and genital specimens to see what level of tenofovir goes to those areas of the body. For example, we will measure how much tenofovir passes from the gel and goes into the blood. We are studying the "pharmacokinetics" of tenofovir; pharmacokinetics is what the body does to a drug, such as where the drug passes and in what amounts.

The strength of the tenofovir **tablets** is the dose approved by the Food and Drug Administration (FDA) for the treatment of Human Immunodeficiency Virus Type 1 (HIV-1) infection in adults. The strength of the tenofovir **gel** has been previously tested and is currently being evaluated in other studies, but is not approved by the FDA for the

treatment or prevention of HIV. HIV is the virus that causes AIDS. This study is not testing to see if tenofovir prevents HIV infection.

WHAT DO I HAVE TO DO IF I AM IN THIS STUDY?
There are three different Study Periods in this study: the Gel Period, the Tablet Period, and the Gel Plus Tablet period. These study visits are part of this study: Enrollment, 3 Week, 6-Week, 7-Week, 10-Week, 13-Week, 14-Week, 17-Week, 20-Week, and 21-Week.

Enrollment Visit:
This visit will take about two hours. At this visit, we will ask you to:

- Sign or make your mark on this consent form after you have had the chance to have all of your questions answered, and if you agree to be in the study
- Answer questions, including questions about your sexual behavior and medical and menstrual history (some of these questions will be asked to make sure that you can be in the study)
- Let us know if there are any changes in how we may contact you
- Tell us about any changes in your medical and menstrual history
- Tell us about any medicines that you are taking now
- Have a brief physical exam
- Have a pelvic exam
- Get treatment for any infections passed during sex or urinary tract infection that you may have, or find out from the study staff where you can get care or treatment
- Hear about:
    - ways to avoid pregnancy
    - how to use male condoms
    - how to avoid infections that may be passed during sex, including HIV
    - the study and how to use the study products the right way
    - what your HIV test results mean (if you have an HIV test today)
- Have your urine tested for pregnancy (your urine may also be tested for urinary tract and/or chlamydia and gonorrhea infection if you are having symptoms)
- Provide blood (14mL) [SITES TO INSERT LOCAL EQUIVALENT] for:
    - tests to check the health of your blood, liver, and kidneys
    - an HIV test (if this is the policy of the study clinic, or the study doctor feels that it is important for your health)
    - a syphilis test (if you have signs of syphilis at this visit)
    - storage at the clinic (this blood may be sent to a laboratory in the US and used for tests on how the body responds to infection)
- Provide a few drops of vaginal discharge (collected by swabs) for testing for infections (only if you are having symptoms of an infection)
- Provide some vaginal and cervical fluid by cervicovaginal lavage (CVL); for CVL, a clinician rinses your vagina and cervix with about 2 teaspoons [SITES TO INSERT LOCAL EQUIVALENT] of sterile fluid and collects that fluid into a tube for testing. A laboratory in the United States will test the fluid to look for

protective proteins and cytokines. Cytokines are very small parts of the fluid that sometimes can be seen when there is inflammation. The protective proteins may help to protect women from infections. These tests do not say if a person has a certain disease or infection. In the future, these tests may help scientists learn more about the safety of different vaginal products. Because doctors do not yet understand enough about what these test results might mean, the results will only be seen by the researchers.

- [AT APPLICABLE SITES] Have a genital swab for herpes if you have signs of herpes
- Receive male condoms from the study staff
- Receive panty liners from the study staff if you use panty liners; a panty liner is a soft absorbent kind of paper that you can place in your panties to catch vaginal discharge or bleeding
- Receive study product(s)
- Receive a small watch and diary card to help you remember or write down what time it is when you take certain dose(s) of study product(s) (we will ask you to tell us the dates and times of your certain doses of study product(s) before coming to your Mid-Study-Period and End of Study Period Visits)

For your study product, you will receive ONE of these ways to take tenofovir today:

1. 28 doses of tenofovir 1% gel already in applicators (an applicator holds the gel and is made to be put in the vagina to insert the gel)

OR

2. 30 tenofovir 300 mg tablets in a bottle

OR

3. 28 doses of tenofovir 1% gel **PLUS** 30 tenofovir 300 mg tablets in a bottle (this means using the gel **AND** taking a tablet every day at about the same time)

By random chance (like flipping a coin [SITES TO INSERT LOCAL EQUIVALENT]) you will be put in a group that takes the study products in a certain order. There are six different groups. For example, one group will use gel first for six weeks, then take tablets for six weeks, and then take both gel and tablets for six weeks. The other groups will have the Gel Period, Tablet Period, and Gel Plus Tablet Period in a different order. All groups will have a week with no study gel or tablets between the study periods, and one week with no study gel or tablets at the end of the study. The study staff will tell you more about how to take care of your gel and tablets and how to use them.

You cannot pick which group you are in (what order you get the study products). The study staff cannot change your group or order of study products. You will only get some of your doses today because you will get more study products at other study visits. There are several times in this study where you will give blood or genital fluid to check for the level of tenofovir. You will not receive the results of these tests because there is not a way to use these results for your medical care.

Mid-Study-Period Visits (3–Week, 10-Week, and 17-Week):
These visits will take about an hour each. At these visits, we will ask you to:

- Tell us if your address has changed, or if there is a new way to reach you
- Tell us about anything new in your medical history, menstrual periods, medicines that you are taking
- Tell us if you are having any health problems or other problems having to do with being in the study
- Have a physical exam
- Have a pelvic exam
- Get treatment or find out where to get treatment for problems (including some infections passed during sex) that the study staff might find
- Hear about:
    - ways to avoid getting pregnant
    - how to use male condoms
- Answer questions about how often you took your study products
- Answer questions about your behavior, including sexual behavior
- Have your urine tested for pregnancy (and infection if you have signs or symptoms)
- Have your blood (6mL) [SITES TO INSERT LOCAL EQUIVALENT] tested for
    - the health of your liver and kidneys
    - tenofovir
    - syphilis (if you have signs of syphilis at this visit)
- Give a few drops of vaginal fluid to be tested for vaginal infections (only if you are having symptoms of vaginal infections, like vaginal itching, for example)
- [AT APPLICABLE SITES] Have a genital swab for herpes if you have signs of herpes
- Receive male condoms
- Receive panty liners, if you wish
- Bring back any study product that you did not use
- Receive more doses of study product

7-Week and 14-Week Visits
These visits will take about an hour and a half each. At these visits, you will have most of the same procedures as the Mid-Study-Period Visits. We will also ask you to:

- Have a test to check the health of your blood (7mL) [SITES TO INSERT LOCAL EQUIVALENT]
- Have an HIV test and hear about what the results mean
- Hear about the study product(s) you will be starting, and how to use them
- Get a new supply of study products

[ONLY **NON-US SITES** TO USE THE FOLLOWING TEXT ON END OF STUDY PERIOD VISITS FOR NON-INTENSIVE PK]

End of Study Period Visits for Non-Intensive PK (6-Week 13-Week, and 20-Week): These visits will take up to nine hours. These visits take longer because we will check your blood twice over a period of 8 hours. These visits will not be scheduled during your menstrual period.

At these visits, you will have many of the same procedures as the Mid-Study-Period Visits. We will also ask you to:

- Take one dose of study gel one tablet, or both in the clinic on the day of your visit, depending on what group you are in
- Give blood (54mL) [SITES TO INSERT LOCAL EQUIVALENT]) for:
    - the level of tenofovir before you take your dose
    - the level of tenofovir one time after your dose of study product(s) in the clinic, either between 1-3 hours, 3-5 hours, or 5-7 hours after your dose, depending on which group you are in. Your group will be decided by a process using random chance (like flipping a coin [SITES TO INSERT LOCAL EQUIVALENT]). You will be able to find out today how long you will have to stay at the clinic for these visits.
    - [AT SITES WITH CAPACITY] flow cytometry -- this test counts certain kinds of cells in your blood and helps us understand why some people's blood takes up more tenofovir than others.
    - tests to check the health of your liver and kidneys
    - storage at the clinic (this blood may be sent to a laboratory in the US and used for tests on how the body responds to infection)
    - [AT SITES WITH CAPACITY] a test to check how much tenofovir gets into different parts of your blood and tissue
- [SITES TO INSERT IF APPLICABLE] Before you give blood for these tests, the study staff may put a special kind of device under your skin in your arm vein called a lock that will let them take your blood without putting a needle into your skin every time. This lock would be removed today after we are done taking the blood samples.
- Provide some vaginal and cervical fluid by CVL. A laboratory in the United States will test the fluid to look for protective proteins and cytokines. We will also check the amount of tenofovir in the fluid.
- It will take about [INSERT TIME] to get the results of your tests. We will give you your results when they are ready. You will not receive the results of some tests (tenofovir levels, flow cytometry), because there is no way to use the results for your medical care. Because doctors do not yet understand enough about what these test results might mean, the results will only be seen by the researchers.

[US SITES ONLY TO INSERT THE FOLLOWING TEXT ON END OF STUDY PERIOD VISITS FOR INTENSIVE PK]

<u>End of Study Period Visits for Intensive PK (6-Week, 13-Week, and 20-Week)</u>
This visit will take up to nine hours. This visit takes longer because we will check your blood six times over a period of 8 hours. These visits will not be scheduled during your menstrual period.

At this visit you will have many of the same procedures as the Mid-Study-Period Visits. We will also ask you to:

- *Tell us when you took your last two doses of study product before coming to the clinic*
- *Take one dose of study gel one tablet, or both in the clinic on the day of your visit, depending on what group you are in*
- *Give blood (134 mL) [SITES TO INSERT LOCAL EQUIVALENT] for these tests:*
    - *flow cytometry--this test counts certain kinds of cells in your blood and helps us understand why some people's blood takes up more tenofovir than others.*
    - *the level of tenofovir before you take a dose of your study product in the clinic*
    - *the level of tenofovir at 1 hour, 2 hours, 4 hours, 6 hours, and 8 hours after your dose of study product(s)*
- *Before you give blood for these tests, the study staff may put a special kind of device under your skin in your arm vein called a lock that will let them take your blood without putting a needle into your skin every time. This lock would be removed today after we are done taking the blood samples.*
- *Give cells from your cervix to check the level of tenofovir there. The cells will be taken with a cytology brush, which is a small brush that is the same kind used to get cells for a Pap smear. (Although the cells will be collected the same way cells are collected for a Pap smear, they will be used for a different kind of test [tenofovir level] so you will not get the results for a Pap smear [a test for cervical cancer] from this test).*
- *Give tissue from two places in your vagina (each about 3 by 5 mm around, or as big as a grain of rice) to check for the level of tenofovir there. We will collect the tissue by taking a biopsy (a biopsy is a sample of tissue) with a special medical tool that is made for this purpose.*
- *Provide some vaginal and cervical fluid by cervicovaginal lavage (CVL); a laboratory in the United States will test the fluid to look for protective proteins and cytokines. We will also check the amount of tenofovir in the fluid*
- *The cervical cells, CVL, and vaginal biopsy samples will be taken one time at each intensive PK visit either before your dose of study product, or at 2, 4, or 6 hours after you receive your study product, depending on random chance (like flipping a coin [SITES TO INSERT LOCAL EQUIVALENT).*
- *It will take about [INSERT TIME] to get the results of your tests. We will give you your results when they are ready. You will not receive the results of some tests*

(tenofovir levels, flow cytometry), because there is no way to use the results for your medical care. Because doctors do not yet understand enough about what these test results might mean, the results will only be seen by the researchers.

Twenty-One-Week Visit
This visit will take about one and a half hours. At the 21-Week Visit, you will have most of the same procedures as the Mid-Study-Period Visits. You will also:

- Have an HIV test and hear about the meaning of the results.
- Give blood for a test to check the health of your blood. (7mL) [SITES TO INSERT LOCAL EQUIVALENT]

The study site staff will give you your test results as soon as they are available. We will ask you to come back to the clinic or, with your permission; we may visit you at your home or a place in your community.

In-Depth Interview
This interview will take about 30 minutes (in addition to the one and a half hours for the other parts of the 21-Week Visit). About eight to ten participants at each study site will be asked to answer extra questions at the 21-Week Visit about what they thought about the study products. The group of possible participants will be chosen by random chance, like flipping a coin [SITES TO INSERT LOCAL EQUIVALENT]. Being in this part of the study (like being in other parts of the study) is voluntary. The interviewers will ask questions about any problems you may have had using the study products, whether you liked using the products or not, any reaction your partner(s) may have had to the product(s), and whether anyone ever wanted you to share or sell them your study products. Since your response to these questions is so important, we will be recording the interview using a handheld digital voice recorder so we can make sure that all the information you provide is captured. Every effort will be made to ensure that all information provided during the interview remains confidential.

AFTER YOU FINISH USING THE GEL AND TABLETS:
During this study you may have a chance to take part in additional studies. If you choose not to take part in any of our additional studies, your participation in this study remains the same. If you have any problems or concerns regarding your health after using the study gel and tablets, let the study staff members know. You can contact the study site staff at any time after you have finished using the study gel and tablets. The study site staff will want to let the study sponsor (the company or organization who provides the study products) know about any serious problems you tell them about.

ANY TIME DURING THE STUDY:
If either you or the study staff members think you may have become pregnant, you will give urine for a pregnancy test. Also, if you are having health problems that may be caused by STIs, you will:

- Have an exam of your genital area and inside your vagina.

- Give blood or urine to test for STIs.
- Get referral for treatment for STIs if you need it.

You are asked to tell the study staff about any medical problems you have. You can contact the study staff between regular visits to report these problems. The study staff will examine you as necessary. They will refer you for medical care that you may need.

When possible, please tell the study site before starting any new medications, supplements, or herbal/traditional remedies.

If the staff members find that study gel and/or study tablets are causing you problems, they may ask you to stop using the study gel and/or study tablets, either for a short time or permanently. The study staff will ask you to stop using the study gel and/or tablets if you become pregnant or if you become infected with HIV or Hepatitis B. Even if you stop using the study gel and/or tablets, you will be asked to stay in the study and have your follow up visits. You will also have some or all of the originally planned exams and tests that the study staff would like you to have to check on your health.

If you have an STI that your partner also may have, you can bring him here for counseling and referral for testing and treatment. You can have extra counseling and testing for HIV at any time during the study. If you wish, your partner can have counseling with you. If you become infected with HIV, you can stay in the study but you cannot keep using the study gel or tablets and you should return any used and unused applicators or tablets to the study clinic. The study staff will give you counseling and refer you to available sources of medical care and other services you may need.

At each study visit, the study staff will update information on where you live and how to keep in contact with you. They will use this information to remind you of scheduled visits. If you miss a visit, the study staff will try to contact you by [SITE-SPECIFIC METHODS]. If you give your permission, they also may visit your home to find you. They will try to reach you through the contact people that you list. If they talk to these people, they will not tell them why they are trying to reach you.

[SITES TO INCLUDE/AMEND THE FOLLOWING IF APPLICABLE:]
[LOCAL/STATE/NATIONAL] regulations require study staff to report the names of people who test positive for HIV and other infections passed during sex to the [LOCAL HEALTH AUTHORITY]. Outreach workers from the [HEALTH AUTHORITY] may then contact you about informing your partners, since they also should be tested. If you do not want to inform your partners yourself, the outreach workers will contact them, according to the confidentiality guidelines of the [HEALTH AUTHORITY].

HOW MANY WOMEN WILL TAKE PART IN THIS STUDY?

Approximately 144 women will take part in this study*:* about 24 each from 5 of the sites (Botha's Hill, Durban, South Africa, Umkomaas, Durban, South Africa, Cleveland, USA, Kampala, Uganda, and Pittsburgh, USA), and 12 each from Birmingham, USA and

Bronx, USA. At US sites (Birmingham, Bronx, Cleveland and Pittsburgh), 72 women will participate in the Intensive PK sampling.

HOW LONG WILL I BE IN THIS STUDY?

You will be in this study about 21 weeks. The total time you will be in the study, including the time to complete the screening exams and tests and the main study is about 23 weeks.

WHY WOULD THE DOCTOR TAKE ME OFF THIS STUDY EARLY?

The study doctor may need to take you off the study early without your permission if:

- The study is cancelled by the US Food and Drug Administration (FDA), US National Institutes of Health (NIH), a drug company supporting this study, the Ethics Committee, the US Office for Human Research Protections (OHRP), the MTN, the local government or regulatory agency, or the Institutional Review Board (IRB). (An IRB is a committee that watches over the safety and rights of research participants).
- The Study Monitoring Committee (SMC) recommends that the study be stopped early (A SMC reviews the progress of the study and the kinds of effects that people report while they are participating in the study).
- You are not able to keep appointments.
- Other reasons that may prevent you from completing the study successfully.

The study doctor will ask you to stop using the study gel or tablets but continue to come in for your follow up visits and procedures if:

- You are pregnant.
- You are breastfeeding.
- You become infected with HIV.
- The study doctor decides that using the study gel or tablets would be harmful to you or your partner.
- You require a treatment that you may not take while using the study gel or tablets.
- You have a bad reaction to the study gel or tablets.

If the study doctor asks you to stop using the study gel or tablets, you will still be advised to come in for all of the scheduled follow-up visits that are described above, including things like the physical exam, vital signs, pelvic exam, blood tests, and questionnaires. You will stop using the study gel or tablets until the study doctor decides it is safe for you to start using the study gel or tablets again, if possible.

WHAT ARE THE RISKS OF THIS STUDY?

Risks of Blood Draws:

You may feel discomfort or pain when your blood is drawn and/or where a lock device for blood draws is inserted. You may feel dizzy, faint or lightheaded. You may have a bruise, swelling, or infection where the needle goes into your arm.

Risks of Genital Exams:
You may feel discomfort or pressure during the exam of your genital area and inside your vagina. You may have mild vaginal spotting (bleeding). The mild bleeding will stop shortly after the exam.

Risks of Cervicovaginal Lavage:
You may feel discomfort or pressure in your vagina and/or pelvis during the CVL.

[US SITES TO ADD THE FOLLOWING LANGUAGE RELATED TO VAGINAL BIOPSIES].

Risks of Vaginal Biopsies:
You may feel slight to moderate pain at the time of the biopsy (like being pinched) which usually resolves quickly but could last for a few hours. You may have spotting (small amounts of vaginal bleeding) for one or two days. You should not have vaginal intercourse until you stop bleeding. You may have a little discomfort (soreness) if you have vaginal intercourse during the time that the biopsy areas are still healing. There is a small risk of the biopsy area becoming infected or having bleeding that is heavier than spotting. Exposure to an STI during vaginal intercourse before complete healing of biopsies might increase your risk of getting an STI. If you have bleeding heavier than your usual menstrual period, a foul odor or a heavier vaginal discharge (more than usual), you should contact the study clinic right away.

Other Possible Risks:
You may become embarrassed, worried, or nervous when discussing sexual behaviors and HIV. You may become worried or nervous while waiting for your STI and HIV test results. If you have HIV, knowing your HIV status could make you worried or nervous. You will talk with a trained staff member who will help you deal with any feelings or questions you have.

Risks of the Study Gel:
It is very important to use the study gel as instructed by staff. The study gel used in this study may have side effects, some of which are listed below. Please note that these lists do not include all the side effects seen with these products. These lists include the more serious or common side effects with a known or possible relationship. If you have questions concerning additional study product side effects please ask the study staff.

Some of the effects of the tenofovir gel are still unknown. Some possible effects are dryness, itching, burning, or pain in the genital area. You may also have discharge if the study gel comes out of the vagina. In about half of the women tested before, there was a small amount of irritation in the genital area.

It is possible that tenofovir gel could be absorbed from the vagina into the blood. Based on an earlier study of tenofovir gel, a small amount of tenofovir gel from the vagina was absorbed into the blood in about half of the women tested (about 1% of the amount that

is absorbed when the oral pill is taken). If the tenofovir in the study gel is absorbed into the blood, it is not known whether this will cause any bad effects. If bad effects did occur, they might be like the ones listed below for the tenofovir tablet.

If you are HIV positive you should not receive the study gel. This is because it is not known what effect tenofovir gel could have on the HIV virus. There is a small possibility that tenofovir could change the virus. If the virus changes, normal treatment for HIV may not work on the virus.

If you have a positive test for hepatitis B you should not receive the study gel. It is not known what effect tenofovir gel could have on the hepatitis B virus. There may be a risk that tenofovir will change the hepatitis B virus. If the virus changes normal treatment for hepatitis B may not work on the virus. It is not known what effect tenofovir gel could have on the disease condition in people with hepatitis B virus.

Your male sexual partners will be protected from potential risks associated with exposure to tenofovir gel through:

- Consistent use of approved male condoms during penile-vaginal sex
- Avoidance of oral-vaginal sex

Risks of the Study Tablet (Tenofovir or TDF 300 mg Tablet)

General Disclaimer
The drugs used in this study may have side effects, some of which are listed below. Please note that these lists do not include all the side effects seen with these drugs. These lists include the more serious or common side effects with a known or possible relationship. If you have questions concerning additional study drug side effects please ask the medical staff at your site.

Use of Combination Antiretroviral Drugs
The use of potent antiretroviral drug combinations may be associated with an abnormal placement of body fat and wasting. Some of the body changes include:

- Increase in fat around the waist and stomach area
- Increase in fat on the back of the neck
- Thinning of the face, legs and arms
- Breast enlargement

Tenofovir itself is not a combination antiretroviral drug, but is one of the drugs that is used in some combination antiretroviral drugs.

Nucleotide Analogue
Lactic acidosis (elevated lactic acid levels in the blood) and severe hepatomegaly (enlarged liver) with steatosis (fatty liver) that may result in liver failure, other complications or death have been reported with the use of antiretroviral nucleoside analogues alone or in combination. The liver complications and death have been seen

more often in women on these drug regimens. Some nonspecific symptoms that might indicate lactic acidosis include: unexplained weight loss, stomach discomfort, nausea, vomiting, fatigue, cramps, muscle pain, weakness, dizziness and shortness of breath.

Tenofovir Disoproxil Fumarate (Tenofovir DF, TDF, Viread®)
Gilead Sciences, Inc.
The following side effects have been associated with the use of tenofovir:

- Upset stomach, vomiting, gas, loose or watery stools
- Dizziness
- Abdominal pain
- Lack of energy
- Kidney damage or failure
- Inflammation or swelling and possible damage to the pancreas
- Shortness of breath
- Rash
- Low phosphate, a chemical in the blood
- Increase of liver functions tests in children
- Allergic reaction, which may include fever, rash, upset stomach, vomiting, loose or watery stools, abdominal pain, achiness, shortness of breath or a general feeling of illness
- Changes in bone growth and strength were seen in study animals given tenofovir. Bone thinning has been seen in adults and children taking tenofovir.

NOTE: If you are infected with both Hepatitis B and HIV, you should be aware that your liver function tests may increase, and symptoms associated with hepatitis (an acute inflammation of the liver) may worsen if tenofovir is stopped. People with hepatitis B infection are not permitted to join this study. If you think you might have been exposed to hepatitis B infection, please tell the study site.

There is a risk that taking tenofovir tablets could change the type of HIV virus in you or your sexual partner's body, if either of you have an HIV infection. This kind of change can make an HIV infection more difficult to treat with HIV medicines. People with HIV infection are not permitted to join this study. If you think you might have been exposed to HIV infection, please tell the study site. If you have questions about side effects, ask a member of the study staff. You should report any new or continuing symptoms to the study staff right away.

Possible Risks to Your Privacy

We will make every effort to protect your privacy while you are in this study. However, it is possible that you could have problems if people learn that you are in this study. They might think that you are infected with HIV or at risk of HIV because of sexual behavior or illegal drug use. Because of this, others may treat you unfairly. For example, you could have problems getting or keeping a job. You also could have problems being accepted by your family or community. There also is a risk to your privacy if someone else taking part in this study knows you.

Are There Risks Related To Pregnancy?

Taking tenofovir gel and tenofovir tablets are not ways to prevent pregnancy. The study gel should not be used during pregnancy. You must agree to try to not become pregnant during the study. It is not known if the study gel used in this study harms unborn babies. You and your partner must be willing to use an effective method of birth control such as birth control pills or another hormonal based method (except for vaginal rings), an intrauterine device or IUD, already be sterilized, or have sex with a partner who is sterilized. You should discuss this with the study staff. Some of these services may be available at your study site.

The study staff will provide male condoms to you free of charge. You must have a pregnancy test before you enter this study. The test must show that you are not pregnant.

Oral tenofovir disoproxil fumarate is classified by the FDA as a pregnancy category B drug. This means that animal studies have not shown a risk to the fetus (unborn baby), but there are no adequate and well-controlled studies in pregnant women that have been completed to date.

NOTE: Because there is only a small amount of information on tenofovir in pregnant women, tenofovir should be used during pregnancy only if clearly needed.

Again, you must agree to try to not become pregnant in this study, and you should tell the study staff if you think you might be pregnant.

What If I Have A Positive Pregnancy Test During The Study?

If you think you may be pregnant at any time during the study, tell your study staff right away. The study staff will talk to you about your choices. If you have a positive pregnancy test while using the study gel or tablets, we will ask you to stop using the study gel or tablets and return all used and unused applicators and tablets, but will ask you to continue to be in the study and to come in for your follow up visits. There are no anticipated additional risks to you if you choose to continue to take part in this study.

If you are pregnant and choose to continue the pregnancy, this study will not provide care related to your pregnancy, the delivery of your baby, or the care of the baby. Your baby may have been exposed to tenofovir if you received tenofovir gel and/or tablets, and we do not know if this will affect unborn babies. The study staff will contact you to ask you a few questions about the outcome of your pregnancy. You must arrange for your care and your baby's care outside of this study. This study cannot provide care related to termination of pregnancy, though study staff can provide you with information regarding your access to termination of pregnancy as part of counseling you about your pregnancy test results.

Breastfeeding

It is unknown if there are any effects of tenofovir on breast milk. It is not known if tenofovir tablets or gel will pass through breast milk and cause harm to your infant. You

must agree to not breastfeed during this study. Women who are currently breastfeeding may not enroll in this study.

ARE THERE BENEFITS TO TAKING PART IN THIS STUDY?

No one knows if the study gel or tablets will prevent HIV infection. Information learned from this study may help in the development of ways to prevent the spread of HIV in the future. You will receive pelvic exams and counseling and testing for HIV and STIs. You will also have tests to check the overall health of your liver, kidneys, and blood cells. This study cannot provide you with medical care, but study staff will refer you to other available sources of care. If your Pap test result shows anything that is not normal, you will be referred for advice and/or treatment.

You will get counseling and testing for HIV. You will get free male condoms. If you are infected with HIV, you will be referred for medical care, counseling, and other services available to you. Medical care for HIV infection will not be part of this study. You will need to get medical care for your HIV infection from your own health care provider or we will provide you with referral to a center that can provide you with appropriate care. We will help you to access the right treatment for HIV infection if you need it. You will get counseling and testing for STIs. If you have an STI diagnosed, you will get medicine to treat it, if needed. You can bring your partner here for counseling and referral for testing and treatment for STIs if this is needed.

WHAT OTHER CHOICES DO I HAVE BESIDES THIS STUDY?

You do not have to participate in this study, if you choose not to. [SITES TO INCLUDE/AMEND THE FOLLOWING IF APPLICABLE: There may be other studies going on here or in the community that you may be eligible for. If you wish, we will tell you about other studies that we know about. There also may be other places where you can go for HIV counseling and testing. We will tell you about those places if you wish.] Please talk to your health provider about these and other choices that may be available to you.

WHAT ABOUT CONFIDENTIALITY?

Efforts will be made to keep your personal information confidential. We cannot guarantee absolute confidentiality. In some situations, including emergencies, legal and professional rules may force us to share confidential information about you. Any publication of this study will not use your name or identify you personally.

Your records may be reviewed by:

- The US Food and Drug Administration (FDA)
- US National Institutes of Health (NIH)
- US Office for Human Research Protections (OHRP)
- Local regulatory authorities
- [INSERT NAME OF SITE] IRB
- Study staff
- Study monitors
- Ethics committees

- The companies that make the gel and the tablets

[For US sites only:] In addition to the efforts made by the study staff to keep your personal information confidential, a Certificate of Confidentiality has been requested from the US Federal Government for this study. This Certificate protects study staff from being forced to tell people who are not connected with this study, such as the court system, about your participation or information you give for study purposes. Even with the Certificate of Confidentiality, however, if the study staff learns of possible child abuse and/or neglect or a risk of harm to you or others, they will be required to tell the proper authorities. Having a Certificate of Confidentiality does not prevent you from releasing information about yourself and your participation in the study.

You are encouraged but not required to tell sexual partners about your being in this study.

WHAT ARE THE COSTS TO ME?

There is no cost to you for study related visits, study products, physical examinations, laboratory tests or other procedures.

WILL I RECEIVE ANY PAYMENT?

You will receive payment for your time and effort in this study. You will receive [INSERT SITE-SPECIFIC AMOUNT OF MONEY] per visit. You will also receive payment for activities affected by your participation in this study [SUCH AS CHILD CARE, TRAVEL, LOSS OF WORK TIME – SITES TO COMPLETE].

WHAT HAPPENS IF I AM INJURED (EXPERIENCE HARM)?

If you are injured as a result of being in this study, the [INSTITUTION] will give you immediate treatment for your injuries. You [will/will not] have to pay for this treatment. You will be told where you can get additional treatment for your injuries. The US National Institutes of Health (NIH) does not have a program to provide money or other forms of compensation for your injuries. Signing this consent form does not change your legal rights.

[SITES TO SPECIFY INSTITUTIONAL POLICY]

WHAT ARE MY RIGHTS AS A RESEARCH PARTICIPANT/VOLUNTEER?

Taking part in this study is completely voluntary. You may choose not to take part in this study or leave this study at any time. You will be treated the same no matter what you decide. If you choose not to participate or to leave the study, you will not lose the benefit of services to which you would otherwise be entitled at this clinic. We will tell you about new information from this or other studies that may affect your health, welfare or willingness to stay in this study. If you want the results of the study, let the study staff members know.

WHAT DO I DO IF I HAVE PROBLEMS OR QUESTIONS?

For questions about this study or a research-related injury, contact:

- [SITE INSERT NAME OF THE INVESTIGATOR OR OTHER STUDY STAFF]

- [SITE INSERT TELEPHONE NUMBER AND PHYSICAL ADDRESS OF ABOVE]

For questions about your rights as a research participant, contact:
- [SITE INSERT NAME OR TITLE OF PERSON ON THE INSTITUTIONAL REVIEW BOARD (IRB) OR OTHER ORGANIZATION APPROPRIATE FOR THE SITE]
- [SITE INSERT TELEPHONE NUMBER AND PHYSICAL ADDRESS OF ABOVE]

SIGNATURE
[INSERT SIGNATURE BLOCKS AS REQUIRED BY LOCAL IRB]

If you have read the informed consent (or had it read and explained to you), and all your questions have been answered and you agree to take part in this study, please sign your name or make your mark below.

Please mark one of the following boxes if you are chosen to participate in the in-depth interview:

☐ I agree to participate in the in-depth interview

☐ I do not agree to participate in the in-depth interview but would still like to participate in this study

______________________________	____________________________________
Participant’s Name (print)	Participant’s Signature or Mark and Date
______________________________	____________________________________
Study Staff Conducting Consent Discussion (print)	Study Staff Signature and Date
______________________________	____________________________________
Witness’ Name (print) (As appropriate)	Witness’s Signature and Date

APPENDIX VIII: SAMPLE INFORMED CONSENT (STORAGE AND FUTURE TESTING OF SPECIMENS)

Phase 2 Open Label Adherence and Pharmacokinetics Study of Oral and Vaginal Preparations of Tenofovir

Version 2.0
03 September 2008

**PRINCIPAL INVESTIGATOR:**	**[insert]**
**PHONE:**	**[insert]**
**Short Title for the Study:**	Adherence and Pharmacokinetics Study of Oral and Vaginal Tenofovir

INTRODUCTION

You have decided to take part in a Division of AIDS research study. While you are in this research study there may be some samples of tissue, blood and/or fluid from your cervix and vagina taken from you that might be useful for future research. You are being asked to agree to the storage of these samples. This consent form gives you information about the collection, storage and use of your samples. The study staff will talk with you about this information. Please ask any questions, if you have some. If you agree to the storage of your samples, you will be asked to sign or make your mark on this consent form. You will be given a copy of this form copy to keep.

HOW WILL YOU GET THE SAMPLES FROM ME?

The research doctors want to save any extra tissue, blood and cervical and vaginal fluid leftover from your tests during the study. This leftover blood and cervical and vaginal fluid will be kept and used for future research.

HOW WILL YOU USE MY SAMPLES?

Your samples will be used to look for ways that your body responds to infection (such as cells, proteins, and other chemicals in your body). Tests may also include checking your genes (material passed from parent to child that determines the make-up of the body and mind), since they might affect how your body responds to disease. Your genes might make you more or less likely to get an infection, affect your responses to infection, or make your responses to treatment stronger or weaker. No other kinds of genetic test will be done on your stored samples without first explaining the test to you and getting your permission. The researchers do not plan to contact you or your regular doctor with any results from tests done on your stored samples. This is because research tests are often done with experimental procedures, so the results from one research study are generally not useful for your medical care. If a rare situation came up where the researchers decided that a test result would provide important information for your health, the researchers would tell your study doctor and your study doctor would try to contact you. If you wish to be contacted with this type of test result, you must give the study doctor or nurse any change to your address and/or phone number. If you want

your regular doctor to be told about this type of test result, you must provide the study doctor or nurse with your regular doctor's name, address and phone number. Your samples will not be sold or used directly to produce products that can be sold for profit.

Research studies using your samples will be reviewed by the National Institutes of Health and a special committee at the researcher's institution (an Institutional Review Board) whose purpose is to protect you as a research participant.

HOW LONG WILL YOU KEEP MY SAMPLES?
There is no time limit on how long your samples will be stored.

HOW WILL MY SAMPLES BE STORED?
Your samples will be stored at special facilities at your study site and/or in the United States that are designed to store samples securely. The storage facilities are made so that only approved researchers will have access to the samples. An Institutional Review Board will oversee the storage facilities to protect you and other research volunteers from harm.

DOES STORAGE OF MY SAMPLES BENEFIT ME?
There are no direct benefits to you.

WHAT ARE THE RISKS?
There are few risks related to storing your samples. When tests are done on the stored samples there is a small but possible risk to your privacy. It is possible that if others found out information about you from tests (such as information about your genes) it could cause you problems with your family (having a family member learn about a disease that may be passed on in families or learning who is the biological parent of a child) or problems getting a job or insurance.

WHAT ABOUT CONFIDENTIALITY?
To keep your information private, your samples will be labeled with a code that can only be traced back to your research clinic. Your personal information (name, address, phone number) will be protected by the research clinic. When researchers are given your stored samples to study they will not be given your personal information. The results of future tests will not be included in your health records.

[For US sites only:] We will do everything we can to protect your privacy. In addition to the efforts of the study staff to help keep your personal information private, we have applied for a Certificate of Confidentiality from the US Federal Government. This certificate means that researchers cannot be forced to tell people who are not connected with the research, such as the court system, about your participation. Also, any publication of the research will not use your name or identify you personally.

People who may review your records include: [INSERT NAME OF SITE] IRB, National Institutes of Health (NIH), US Office for Human Research Protections, US Food and Drug Administration, study staff, study monitors, and their designees. Having a

Certificate of Confidentiality does not prevent you from giving information about yourself and your participation in the study. Even with the Certificate of Confidentiality, if the study staff learns of possible child abuse and/or neglect or a risk of harm to you or others, we will tell the proper authorities.

WHAT ARE MY RIGHTS?

Allowing your samples to be stored is completely voluntary. You may decide not to have any samples stored other than what is needed to complete this study and still be in this research study or any future study. If you decide now that your samples can be stored for future research, you may change your mind at any time. You must contact your study doctor or nurse and let them know that you do not want your samples used for future research. Your samples will then not be used and will be destroyed.

WHAT DO I DO IF I HAVE QUESTIONS?

For questions about the storage of your samples, contact (*insert the name of the investigator*) at *(insert telephone number)*.

For questions about your rights related to the storage of your samples for research, contact (*insert the name or title of person on the Institutional Review Board) at (insert telephone number)*.

SIGNATURE PAGE
[INSERT SIGNATURE BLOCKS AS REQUIRED BY LOCAL IRB]

If you have read the informed consent (or had it read and explained to you), and all your questions have been answered and you agree to this specimen storage and future testing, please sign your name or make your mark below.

______________________________	______________________________
Participant's Name (print)	Participant's Signature or Mark and Date
______________________________	______________________________
Study Staff Conducting Consent Discussion (print)	Study Staff Signature and Date
______________________________	______________________________
Witness' Name (print) (As appropriate)	Witness's Signature and Date

REFERENCES

1 Gilead Sciences. Viread (tenofovir disoproxil fumarate) tablets. Package Insert, May 2008.

2 CONRAD. Investigator's Brochure: Tenofovir Gel (GS-1278). Third Edition, 11 January 2008.

3 Tarantal AF, Castillo A, Ekert JE et al. Fetal and maternal outcome after administration of tenofovir to gravid rhesus monkeys (Macaca mulatta). JAIDS 2002; 29: 207-220.

4 Lund B, Weber S. Pharmacokinetics and distribution of intravaginal [^{14}C] PMPA (GS-1278) following single dose administration to rats. Gilead Sciences Report No. 96-DDM-1278-004. October 1, 1996.

5 Creek MR. Pharmacokinetics and tissue distribution study of PMPA (GS-1278) following intravaginal administration in female New Zealand White Rabbits. SRI Report No. B010-97. November 6, 1997.

6 Fairchild D. 14-day vaginal tissue irritation and toxicity study of PMPA in rats. Report No. M016-97. June 30, 1997.

7 Peterson L, Taylor D, Roddy R, et al. Tenofovir disoproxil fumarate for prevention of HIV infection in women: a phase 2 double-blind randomized placebo-controlled trial. PLoS Clin Trials 2(5): e27.doi:10.1371/journal.pctr.0020027

8 Mayer K, Maslankowski L, Gai F, et al. Safety and tolerability of tenofovir vaginal gel in abstinent and sexually active HIV-infected and uninfected women. AIDS 2006;20:543-551.

9 CONRAD Protocol #A04-099 (Study Report), Male Tolerance Study, July 19, 2007.

10 Hillier SL. Safety and acceptability of daily and coitally dependent use of 1% tenofovir over six months of use. In: Microbicides 2008; New Delhi.

11 NIAID Study 97-101. Pharmacokinetic Study in Three Monkeys.

12 Ezzet F, Whitehead J. A Random Effects Model for Binary Data from Crossover Clinical Trials. Applied Statistics 1992;41(1):117-126.

[13] Koch GG, Stokes ME. Chi-Square Tests: Numerical Examples. In Kotz S and Johnson N, eds. Encyclopedia of Statistical Sciences, Volume 1. New York, NY:John Wiley & Sons, 1982:457-472.

[14] McCullagh P. Regression models for ordinal data. Journal of the Royal Statistical Society, Series B, 1980;42:109-142.
